# MeCP2 regulates cell-type-specific functions of depressive-like symptoms in the nucleus accumbens

**DOI:** 10.1038/s12276-026-01721-3

**Published:** 2026-05-12

**Authors:** Jinhee Bae, Sung Hoon Kim, Sun A Jung, Nazarii Frankiv, Hyunjeong Jin, Eun Mi Hwang, Young-Min Kim, Sangjoon Lee, Heh-In Im

**Affiliations:** 1 Center for Brain Disorders, Brain Science Institute, Korea Institute of Science and Technology, Seoul, Republic of Korea; 2 Biomaterials Research Center, Korea Institute of Science and Technology, Seoul, Republic of Korea; 3 Division of Bio-Medical Science and Technology, KIST School, Korea University of Science and Technology, Seoul, Republic of Korea

**Keywords:** Depression, Striatum

## Abstract

Methyl-CpG binding protein 2 (MeCP2) is a chromatin-associated transcriptional regulator that modulates neuronal gene programs in response to environmental stimuli. Although MeCP2 has been implicated in stress responses and depression, its cell-type-specific functions within defined limbic circuits remain incompletely understood. Here, using a chronic restraint stress (CRS) model, we show that CRS selectively reduces MeCP2 protein in dopamine D2 receptor (D2R)-expressing medium spiny neurons in the nucleus accumbens (NAc). D2R-restricted MeCP2 knockdown was sufficient to increase immobility in the forced swim test, whereas Cre-dependent restoration of MeCP2 in NAc D2R neurons attenuated CRS-associated behavioral alterations across affective coping, anxiety-like behavior and reward sensitivity. Ex vivo multielectrode array recordings combined with optogenetic stimulation revealed that CRS-associated suppression of NAc activity was normalized toward control levels by MeCP2 restoration. To profile molecular correlates, we performed cell-type-resolved GeoMx digital spatial transcriptomics in virally labeled NAc D2R neurons and found that MeCP2 overexpression was associated with attenuation of CRS-linked transcriptional perturbations, prominently involving synaptic and neuronal communication-related programs. In parallel, we detected CRS-responsive molecular signatures in the ventral pallidum that shifted with NAc D2R-restricted MeCP2 restoration, although these downstream profiles are not projection-resolved. Collectively, our findings identify a D2R neuron-biased role for MeCP2 in the NAc and support the view that restoring MeCP2 in this cell population is associated with mitigation of CRS-induced depression-like phenotypes and accompanying circuit/transcriptomic signatures.

**Figure:**
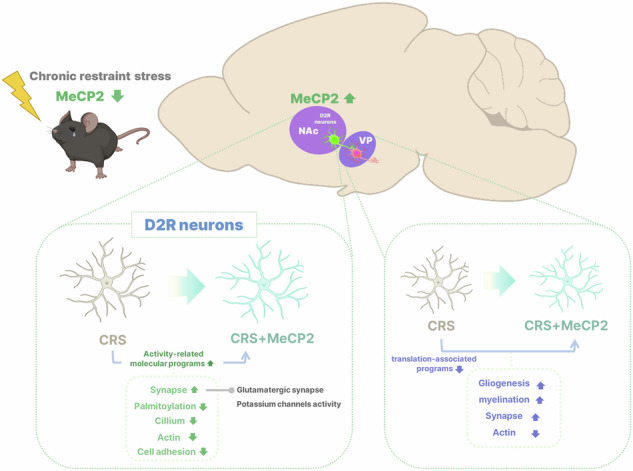
Chronic restraint stress (CRS) reduces MeCP2 protein in nucleus accumbens (NAc) D2 receptor (D2R)-expressing neurons, suppressing activity- and synapse-related programs and promoting depressive-like behaviors. Cell-type-specific restoration of MeCP2 in NAc D2R neurons normalizes neuronal activity and attenuates behavioral deficits, accompanied by coordinated transcriptional shifts involving synaptic organization, glutamatergic signaling, potassium channel activity and cytoskeletal regulation. CRS-responsive molecular signatures in the ventral pallidum (VP) show partial normalization in association with MeCP2 upregulation (VP bulk ROIs). Together, these findings implicate MeCP2-dependent regulation of NAc D2R neuron state in stress-related outcomes.

Chronic restraint stress (CRS) reduces MeCP2 protein in nucleus accumbens (NAc) D2 receptor (D2R)-expressing neurons, suppressing activity- and synapse-related programs and promoting depressive-like behaviors. Cell-type-specific restoration of MeCP2 in NAc D2R neurons normalizes neuronal activity and attenuates behavioral deficits, accompanied by coordinated transcriptional shifts involving synaptic organization, glutamatergic signaling, potassium channel activity and cytoskeletal regulation. CRS-responsive molecular signatures in the ventral pallidum (VP) show partial normalization in association with MeCP2 upregulation (VP bulk ROIs). Together, these findings implicate MeCP2-dependent regulation of NAc D2R neuron state in stress-related outcomes.

## MeCP2 restoration in D2R neurons alleviates stress effects

This study explores the role of MeCP2, a transcriptional regulator, in stress-related behaviors and neural adaptations, focusing on its cell-type-specific effects in the nucleus accumbens (NAc). Researchers used a chronic restraint stress (CRS) model to show that stress reduces MeCP2 levels specifically in dopamine D2 receptor (D2R) neurons, leading to depressive-like behaviors. By restoring MeCP2 expression in these neurons, they reversed stress-induced behavioral changes, such as increased immobility and reduced sucrose preference, indicating improved stress resilience. The study employed techniques like multielectrode array recordings and spatial transcriptomics to reveal that MeCP2 restoration normalizes neural activity and transcriptional profiles in D2R neurons and the ventral pallidum, a downstream target. These findings highlight MeCP2’s potential as a therapeutic target for stress-related disorders, suggesting future research should explore projection-specific mechanisms and broader circuit-level impacts.

This summary was initially drafted using artificial intelligence, then revised and fact-checked by the author.

## Introduction

Major depressive disorder is associated with persistent alterations in stress reactivity, affective behavior and neural circuit dynamics. Identifying the molecular regulators that determine stress susceptibility versus resilience remains a critical gap in the field. Methyl-CpG binding protein 2 (MeCP2) is a chromatin-associated transcriptional regulator that integrates neuronal activity with gene expression programs^1–6^. Although MeCP2 is best known for its developmental role and association with Rett syndrome, emerging evidence indicates that it also modulates adult stress adaptation and emotional regulation^4,6,7^. For example, changes in MeCP2 expression and phosphorylation have been reported following chronic stress, antidepressant treatment and ketamine exposure^7,8^, and peripheral MeCP2 reductions have been observed in individuals experiencing depressive symptoms^4^. However, despite these observations, the mechanisms by which MeCP2 contributes to affective behavioral regulation—particularly under chronic stress conditions—remain poorly understood. Notably, whether MeCP2 acts in a cell-type-specific manner within defined limbic circuits to shape stress susceptibility versus resilience has not been directly addressed.

The nucleus accumbens (NAc) is a key node in limbic circuitry that integrates motivational, emotional and stress-related signals^9–11^. Neurons in the NAc are predominantly composed of dopamine D1 receptor-expressing (D1R) and dopamine D2 receptor-expressing (D2R) populations, which exert dissociable and often opposing influences on behavior^12,13^. D1R MSNs are generally linked to behavioral activation and positive valence, whereas D2R MSNs are associated with aversion, withdrawal states and stress-induced behavioral change. Dysregulated signaling within these neuronal populations has been repeatedly implicated in stress-related behavioral dysfunction and circuit maladaptation^9,14,15^.

Here, we used a chronic restraint stress (CRS) model to examine whether MeCP2 is associated with stress-related behavioral and circuit changes in a cell-type-specific manner. We found that CRS was accompanied by reduced MeCP2 protein levels in D2R neurons, but not D1R neurons, in the NAc. Viral restoration of *MeCP2* expression specifically in D2R neurons attenuated CRS-associated behavioral alterations, including increased immobility in the forced swim test (FST) and elevated anxiety-like behavior in the elevated plus maze (EPM). In addition, we included a reward-related readout (sucrose preference test, SPT) in the *MeCP2* restoration experiment to assess reward sensitivity (anhedonia-related behavior). To further characterize the neurobiological correlates of these effects, we performed multielectrode array (MEA) recordings and spatial transcriptomics, which revealed activity patterns and transcriptional profiles that shifted toward baseline in the recovery condition and were consistent with attenuation of CRS-associated transcriptional changes in NAc D2R neurons. Parallel transcriptional changes were also observed in the ventral pallidum (VP).

Together, these findings indicate that MeCP2 in NAc D2R neurons plays a central role in stress-related behavioral and molecular adaptations, and that cell-type-targeted restoration of *MeCP2* is sufficient to attenuate multiple CRS-associated phenotypes. In parallel, MeCP2 restoration is accompanied by coordinated molecular changes in downstream regions, including the VP. While the present study does not resolve projection-specific mechanisms or a single causal pathway linking these effects, the data support a model in which MeCP2-dependent regulation in NAc D2R neurons is contributes to broader circuit-level and transcriptomic adaptations under chronic stress.

## Materials and methods

### Animals

We used 7–9-week-old male C57BL/6J (DBL), B6.FVB (Cg)-Tg (*Drd2*-cre) ER44Gsat/Mmcd (#032108-UCD, MMRRC) or B6. Cg-Tg (*Drd1*-cre) 262Gsat/Mmcd (#030989-UCD, MMRRC) mice for experimental procedures. All transgenic mice for experiments were backcrossed to wild-type C57BL/6J mice for several generations. All mice were housed under standard conditions at 21–23 °C, 50–55% humidity and a 12-h light/dark cycle. All behavioral procedures were performed during the dark cycle. All experimental procedures were approved by the Animal Care and Use Committee of the Brain Science Institute of the Korea Institute of Science and Technology (KIST).

### CRS model

Mice were restrained in a square restraint device with an internal area of 3 (width) × 3 (height) × 17 (length) cm. They were secured inside the restraint device by a movable fixation wall of approximately 4.0 cm so that they could not move freely. In the space inside the restraint device, the mice could slightly change their body position but could not move forward or backward. The mice were held simultaneously for 6 h per day for 21 days. The control group received no disturbance except for 3 min of handling per day during the same period. Both groups received regular cage maintenance and body weight measurements during the CRS exposure period.

### Behavioral tests

#### Open field test

The locomotor activity of mice was analyzed using an open field test. The behavioral box of the open field test consisted of a white acrylic square arena (40 cm × 40 cm) and a 40-cm white wall. On the day after the CRS session, both the CRS and control (CTR) groups were allowed to freely explore the box for 30 min, and their movements were recorded with an infrared camera. The analysis was performed using EthoVision software (Noldus). The center zone was assigned to the central square area (20 cm × 20 cm). Locomotor activity was measured as the total distance traveled and the distance traveled in the center zone as dependent variables.

#### EPM test

An EPM test was conducted to determine the anxiety level of mice. The EPM box consisted of two white open arms and two black closed arms. The closed arms had a 15-cm-high wall, and the open arms had no wall. The height of the box was 44 cm. The mice were placed in the central area of the EPM box, and the movements of the mice were recorded for 10 min using a video camera. The time spent in the open and closed arms was measured manually, and time in the arms was recorded only when all four paws of the mice entered the arms. The time spent in the central area was excluded from the measurement.

#### FST

A FST to measure depressive symptoms in mice was performed in a cylinder 20 cm high and 15 cm in diameter for 6 min. The water temperature was 24 ± 1 °C, and fresh water was replaced immediately after each trial. Immobility was recorded during the last 4 min of the 6-min period and analyzed manually. Mobile time was measured when the animals swam or moved by moving at least three limbs, including both forelimbs.

#### SPT

The SPT was performed to assess anhedonia after completion of the CRS procedure. On the final day of CRS, mice were transferred to individual SPT cages for habituation with ad libitum access to two bottles of tap water and their regular standard chow (seven full pellets, identical to their home-cage diet) for 24 h. The SPT began at 10:00 on the following day. At the start of the test, each cage was provided with one bottle containing 1% (w/v) sucrose solution and one bottle containing tap water, with each bottle containing approximately 85 g of liquid. The test lasted for 48 h. After 24 h, the positions of the sucrose and water bottles were switched to prevent side bias, without changing the contents. At 48 h, both bottles were reweighed to determine total fluid intake over the 48-h period. All tests were conducted under a 12-h light/dark cycle controlled by an automatic timer. No food or water deprivation was applied at any stage of the procedure to ensure that the SPT selectively reflected hedonic drive rather than deprivation-induced drinking. Sucrose preference (%) was calculated as: Sucrose preference (%) = [sucrose intake (g over 48 h)/(sucrose intake (g over 48 h) + water intake (g over 48 h))] × 100. SPT was conducted only for the MeCP2 restoration (OE) experiment (NC/CC/CM), and not for the CRS validation or shMeCP2 experiments.

#### Novel object recognition test

A novel object recognition test was used to measure cognitive function. In the training trial (familiarization phase), mice were allowed to explore two identical objects placed at regular intervals in a white square box (40 × 40 × 40 cm, center: ~5 lx; periphery: ~4 lx) for 10 min. The test trial (recognition phase) was performed 24 h after the training trial for 10 min. Mice explored two different objects (familiar and novel objects). The behavior of mice was video recorded, and the smell time of the familiar or novel object was manually analyzed and compared during the test phase.

#### Stereotaxic injection and AAVs

Under ketamine–xylazine anesthesia, stereotaxic injections of virus into the NAc were performed using a stereotaxic device (David Kopf Instruments). First, 500 nl of concentrated virus solution was injected bilaterally into the NAc (anteroposterior (AP) +1.4 mm; mediolateral (ML) ±1.15 mm; ventral–posterior −4.1 mm) using a syringe pump (Harvard Apparatus) at a slow rate (100 nl/min). The needles were slowly withdrawn 10 min after the injection. In experiments requiring insertion of an optogenetic cannula, the needles were removed and the optogenetic cannula was implanted bilaterally into the NAc (AP + 1.4 mm; ML ± 1.15 mm; ventral–posterior −3.8 mm). Mice were used 2–3 weeks after adeno-associated virus (AAV) injection. We constructed a Cre-dependent AAV vector that we previously developed by inserting a scrambled or mouse *MeCP2* shRNA sequence (5′-GTCAGAAGACCAGGATCTC-3′) for knockout of *MeCP2* (AAV-GFP-creon-mMECP2 shRNA)^16,17^. To increase the expression of MeCP2, the full-length sequence of mouse *MECP2* was inserted into the pAAV-Ef1a-DIO-EGFP-WPRE-pA (Plasmid #37084, Addgene) vector. All vectors were packaged as serotype DJ at the KIST Virus Facility (http://virus.kist.re.kr).

#### Western blot

To analyze proteins in each sample, 40 μg of protein was added to 5× sodium dodecyl sulfate (SDS)–polyacrylamide gel electrophoresis loading buffer and loaded onto 12% Tris-glycine SDS–polyacrylamide gel. The gel was transferred to an immobilon-P transfer membrane (IPVH 00010, Millipore), and the membrane was blocked with 5% skim milk in 0.03% Tris-Bisol-Tris (Tween-20, P2287, Sigma). The membrane was incubated overnight at 4 °C with shaking in blocking buffer containing primary antibodies (rabbit anti-MeCP2 polyclonal antibody, 1:500, 07-013, Millipore; mouse anti-GAPDH, 1:5,000, sc-47724, Santa Cruz; mouse anti-β actin, 1:5,000, sc-47778, Santa Cruz). Membranes were washed three times in TBS-T with shaking for 10 min each and then incubated with secondary antibodies (goat anti-rabbit IgG-HRP, 1:5,000, ab97051, Abcam, or goat anti-mouse IgG-HRP, 1:5,000, sc-2005, Santa Cruz) with shaking at room temperature for 2 h. Membranes were rinsed again and visualized using SuperSignal West Pico Chemiluminescent Substrate (Thermo Scientific) according to the manufacturer’s instructions. Immunoblots were detected with a luminescent image analyzer ImageQuant LAS4000 (GE Healthcare Life Sciences, BUX). Densitometric quantification was performed, and blots were analyzed with ImageJ software.

#### Formalin-fixed paraffin-embedded tissue preparation

Mouse brains were collected and fixed in 4% paraformaldehyde for 24 h at 4 °C. For paraffin embedding, brains were sequentially dehydrated using 40% ethanol, 70% ethanol and a second 70% ethanol wash, each for 24 h. Whole brains were then transferred to embedding cassettes and processed using a Tissue-Tek VIP Tissue Processor (Sakura Finetek). The 12-h processing cycle included one 30-min ethanol wash, five additional 1-h ethanol incubations, three 45-min CitriSolv washes (Decon Labs) and three 1-h paraffin wax infiltrations. For the GeoMx Digital Spatial Profiler (DSP), tissues were paraffin-embedded, and 2-mm-diameter circles of thalamic brain regions, including the NAc and VP, were punched out from individual paraffin blocks and embedded into tissue microarray blocks to facilitate simultaneous processing and analysis.

#### Immunohistochemistry

Mice were deeply anesthetized with Avertin (2,2,2-tribromoethanol; Sigma) 2 h or 3 days after the final CRS session, transcardially perfused with ice-cold 1× phosphate-buffered saline (PBS) and fixed with ice-cold 10% formalin (Sigma). Brains were postfixed overnight at 4 °C, cryoprotected in 30% sucrose and sectioned at 40 μm using a microtome (Leica) at −20 °C. Free-floating sections were incubated overnight at 4 °C with primary antibodies (rabbit anti-MeCP2, 1:250, Millipore, 07-013; mouse anti-MeCP2, 1:250, Abcam, ab50005; rabbit anti-D1DR, 1:50, Abcam, ab20066; rabbit anti-D2DR, 1:50, Santa Cruz, sc-5303; chicken anti-GFP, 1:400, Abcam, ab13970), followed by Alexa Fluor-conjugated secondary antibodies for 2 h at room temperature (Alexa Fluor 488 goat anti-rabbit IgG, 1:400, Life Technologies, A-11008; Alexa Fluor 594 goat anti-rabbit IgG, 1:400, Life Technologies, A-11012; Alexa Fluor 488 goat anti-mouse IgG, 1:400, Life Technologies, A-11034; Alexa Fluor 594 goat anti-mouse IgG, 1:400, Life Technologies, A-11005; goat anti-chicken IgG, 1:400, Abcam, ab150169). Sections were mounted with 4′,6-diamidino-2-phenylindole (DAPI)-containing mounting medium (Vector, H-1500) and imaged using a Zeiss LSM800 confocal microscope.

For paraffin-based immunostaining, formalin-fixed paraffin-embedded brains were sectioned at 5 μm. Sections were deparaffinized in xylene and graded ethanol, followed by antigen retrieval in sodium citrate buffer (pH 8.5) at 80 °C for 30 min. After washing in PBS with Tween-20 (PBST) containing 0.3% Triton X-100, sections were blocked and permeabilized in PBST with 5% donkey or goat serum for 2 h at room temperature, then incubated overnight at 4 °C with primary antibodies rabbit-anti-BDNF, 1:400, Abcam, ab108319; anti-GFP (1:1,000); anti-MeCP2 (1:250); anti-phospho-MeCP2 (Ser421), 1:200, Phospho solutions, p1205-421; anti-CREB, 1:400, Cell Signaling, D76D11; anti-phospho-CREB (Ser133), 1:400, Cell Signaling, 9198. After PBST washes, sections were incubated with Alexa Fluor 488- or 594-conjugated donkey or goat secondary antibodies (Alexa 488 donkey anti-chicken IgG, 1:1,000, Jackson ImmunoResearch, 703-545-155; Alexa 594 donkey anti-rabbit IgG, 1:400, Invitrogen, A-21207) together with DAPI (1:1,000), (Sigma-Aldrich, D9542), mounted with mounting medium (Dako, S3023) and imaged on a Zeiss LSM800 confocal microscope.

Fluorescence quantification was performed in ImageJ by an experimenter blinded to experimental conditions. Confocal images were split into individual channels, and regions of interest (ROIs) were manually defined to separate GFP-positive regions from surrounding non-GFP tissue. Mean fluorescence intensity was measured within ROIs for the target channel, and the GFP-positive/non-GFP ratio was calculated. Data were normalized to the NC group and reported as fold change.

#### BDNF enzyme-linked immunosorbent assay (ELISA)

BDNF levels were measured using a commercial ELISA kit (R&D Systems; Total BDNF). All reagents and samples were equilibrated to room temperature and assayed in duplicate according to the manufacturer’s instructions. In brief, 50 µl of Assay Diluent RD1-123 and 50 µl of standards, controls or samples were added per well and incubated for 2 h at room temperature on an orbital shaker (0.12″ orbit, 500 ± 50 rpm). Plates were washed four times (400 µl per well), followed by incubation with 200 µl of Total BDNF Conjugate for 1 h and washing as above. Substrate solution (200 µl per well) was added for 30 min in the dark, and the reaction was stopped with 50 µl per well Stop Solution. Absorbance was read at 450 nm within 30 min, with 540/570 nm wavelength correction when available (or subtracted from 450 nm).

#### Reverse-transcription qPCR

Total RNA was extracted from homogenized brain tissues using TRIzol reagent (Life Technologies), according to the manufacturer’s instructions. Complementary DNA was synthesized from the extracted RNA using the ReverTra Ace qPCR RT Master Mix (TOYOBO). Real-time quantitative polymerase chain reaction (qPCR) was performed using THUNDERBIRD SYBR qPCR Master Mix (TOYOBO) on a CFX Connect Real-Time PCR Detection System (Bio-Rad). The following primer pairs were used: *MeCP2* (forward: 5′-GAGAGAGCAGAAACCACCTA-3′; reverse: 5′-TCTGATGCTGCTGCCTTT-3′), *Drd2* (forward: 5′-ACTTGTGTGCCATCAGCATC-3′; reverse: 5′-AAGGACAGGACCCAGACGAT-3′) and *Gapdh* (forward: 5′-GACATCAAGAAGGTGGTGAAGC-3′; reverse: 5′-ACCACCCTGTTGCTGTAGCC-3′). Relative mRNA expression levels were calculated using the ∆∆Ct method, with *Gapdh* serving as the internal reference gene.

#### FACS cell sorting

For fluorescence-based cell isolation, eGFP expression was achieved via stereotaxic delivery of Cre-dependent AAV vectors into *Drd1*-Cre or *Drd2*-Cre mice, and GFP-positive (GFP⁺) cells were isolated by fluorescence-activated cell sorting (FACS). Animals were euthanized in accordance with institutional animal care guidelines, and brain tissues were collected immediately post-mortem under sterile conditions. Tissues were mechanically dissociated using fine scissors or razor blades, followed by enzymatic digestion in RPMI 1640 or DMEM supplemented with 1–2 mg/ml collagenase D and 100 μg/ml DNase I at 37 °C for 30–45 min with gentle agitation. The resulting cell suspensions were filtered through a 70-μm cell strainer to obtain single-cell suspensions and centrifuged at 300–400*g* for 5 min. When required, erythrocyte lysis was performed using ACK lysis buffer for 1–3 min at room temperature. Cells were washed and resuspended in FACS buffer (PBS containing 2% fetal bovine serum (FBS) and 2 mM EDTA) and then passed through a 35-μm mesh to remove aggregates prior to sorting. Fluorescence-based cell sorting was performed on a MA900 Cell Sorter (SONY Biotechnology) equipped with appropriate lasers and filters for detection of the specific fluorescent reporter. Gating strategies were established using tissues from nonfluorescent wild-type mice to define thresholds for fluorescence-positive populations. Doublets and dead cells were excluded on the basis of forward and side scatter parameters and viability dye staining. Sorted fluorescent-positive cells were collected into tubes containing a collection buffer (for example, FBS or culture media supplemented with 10–20% FBS) and were either processed immediately for downstream applications or stored at −80 °C or in liquid nitrogen depending on the experimental requirements.

#### GeoMx DSP

RNAscope staining was performed using a RNAscope Multiplex Fluorescent Reagent Kit v2 (Advanced Cell Diagnostics), following the manufacturer’s protocol. Formalin-fixed paraffin-embedded tissue sections were dried at 60 °C for 1 h, deparaffinized and subjected to a series of pretreatment steps including hydrogen peroxide incubation, heat-mediated antigen retrieval and protease digestion. Specific probes targeting *eGFP* (ACD, 538851-C3), *Drd1* (ACD, 461901) and *Drd2* (ACD, 406501-C2) were hybridized at 40 °C for 2 h. Signal amplification was carried out using sequential AMP reagents, and fluorescent signals were visualized with Opal dyes—Opal 520 (Akoya Biosciences, FP1487001KT), Opal 620 (FP1495004KT) and Opal 690 (FP1497001KT). Following hybridization, slides were postfixed and processed according to NanoString’s standard protocol for the GeoMx DSP.

For a spatial transcriptomic analysis, tissue sections were hybridized overnight at 37 °C with probes from the GeoMx Mouse Whole Transcriptome Atlas (NanoString). After hybridization, samples were blocked with Buffer W (NanoString) for 30 min and stained with a visualization cocktail containing a nuclear dye (SYTO 83, NanoString) for 2 h at room temperature in a humidified chamber.

Slides were then imaged on the GeoMx DSP system to generate high-resolution fluorescence maps. ROIs were manually selected based on probe signal intensity and tissue architecture, and molecular compartments were defined using fluorescent markers.

Barcoded oligonucleotides from each ROI were photocleaved using ultraviolet (UV) light and collected via microcapillary aspiration into a 96-well plate. These oligonucleotides were then used for library preparation and sequenced on the Illumina NovaSeq 6000 platform (Theragen Bio) following the manufacturer’s instructions.

Raw barcode counts corresponding to RNA targets were analyzed using the GeoMx software suite. Expression values were normalized using the third quartile (Q3) normalization method to account for variability in hybridization efficiency and technical bias across samples.

#### Sequencing data—processing and QC

Following spatial transcriptomic profiling with the GeoMx DSP, raw sequencing data (FASTQ format) were processed using the GeoMx NGS analysis pipeline. Reads were first assessed for quality (Q30), adapter sequences were trimmed, and paired-end reads were merged to produce stitched reads. These were aligned to probe-specific barcode sequences, and PCR duplicates were removed using unique molecular identifiers, resulting in deduplicated read counts. The processed data were then imported into the GeoMx DSP Data Analysis Suite for quality control (QC) and downstream interpretation.

QC involved two stages: segment-level QC and biological probe QC. For segment QC, the following thresholds were applied: ≥1000 raw reads, ≥50% alignment rate, ≥80% stitched and trimmed reads, ≥50% sequencing saturation, geometric mean of negative probe counts ≥1, no-template control counts <2000, tissue segment area ≥1000 µm², and ≥50 nuclei per segment. Biological probe QC was performed to exclude underperforming probes. Probes were excluded if their mean count was ≤10% of the total probes for a given gene. Likewise, genes were removed from further analysis if their expression levels fell below the limit of quantitation (LOQ) in more than 20% of segments.

The LOQ was calculated as follows: LOQ = GeoMean (NegProbes) × [GeoStdev (NegProbes)]².

This threshold ensured reliable detection of gene expression above background noise. Finally, normalization was conducted across samples using the Q3 normalization method. For each segment, the third quartile of expression values was calculated. The geometric mean of Q3 values across all segments was used to derive a normalization factor for each sample. Each expression value was then scaled by this factor, minimizing the influence of outliers and variability in cell density or RNA capture efficiency across segments.

#### MEA

Artificial cerebrospinal fluid (ACSF) was prepared with the following components: 92 mM N-methyl-D-glucamine (NMDG), 25 mM glucose, 5 mM sodium L-ascorbate, 2.5 mM KCl, 1.25 mM NaH₂PO₄, 2 mM thiourea, 10 mM MgCl₂, 30 mM NaHCO₃, 20 mM HEPES, 3 mM sodium pyruvate and 0.5 mM CaCl₂ (pH adjusted to 7.4). For tissue dissection, a calcium-free variant of ACSF was used by omitting CaCl₂.

Mice were deeply anesthetized and transcardially perfused with ice-cold NMDG-based ACSF. Brains were rapidly removed and transferred to chilled, oxygenated NMDG-ACSF. Using a vibratome (VT1000S; Leica Biosystems), sagittal brain slices were cut at a thickness of 280 μm. The slices were then incubated in warmed (35 °C), carbogen-bubbled (95% O₂/5% CO₂) NMDG-ACSF for 30 min to allow recovery.

MEA plates (M384-tMEA-6W WHITE; Axion Biosystems) were pretreated by incubating them in standard ACSF—composed of 92 mM NaCl, 25 mM glucose, 5 mM sodium L-ascorbate, 2.5 mM KCl, 1.25 mM NaH₂PO₄, 2 mM thiourea, 2 mM MgCl₂, 30 mM NaHCO₃, 20 mM HEPES, 3 mM sodium pyruvate and 2 mM CaCl₂—at room temperature for at least 1 h.

For recording, individual brain slices were carefully transferred onto the MEA surface and overlaid with recording ACSF containing 124 mM NaCl, 12.5 mM glucose, 2.5 mM KCl, 1.25 mM NaH₂PO₄, 2 mM MgCl₂, 2 mM CaCl₂, 26 mM NaHCO₃ and 20 mM HEPES. The MEA plates were then placed into the Maestro Edge system (Axion Biosystems), and spontaneous or optogenetically evoked neural activity was recorded. Electrophysiological data were analyzed using Offline Sorter V4 software (Plexon).

#### Optogenetic stimulation

Optogenetic stimulation was presented using a blue light-emitting diode plate (170 mm × 130 mm, Scitech Korea) on a MEA plate (Fig. 4b). The optogenetic stimulation was delivered to the sagittal brain slices within the plate through the transparent window of the MEA system where the MEA plate was placed (473 nm, 20 Hz, 50% duty cycle, 10 mW/Cm^2^ when measured at the bottom of the plate within the MEA system). The optogenetic stimulation consisted of eight cycles of 10 s blue light presentation followed by 30-s rest periods. The expression of channelrhodopsin in the MEA NAc was confirmed previously using a confocal microscope (FluoView FV1000, Olympus).

#### Statistical analysis

Two-tailed and unpaired or paired *t*-tests were used in experiments comparing two groups. One-way analysis of variance (ANOVA) was used for comparing more than two groups and two-way mixed factorial ANOVA was used in experiments requiring between and within group analysis with GraphPad Prism 10. Transcriptome analysis was carried out using Gene Ontology (GO) analysis in ToppGene (https://toppgene.cchmc.org/), functional annotation clustering analysis in DAVID (https://davidbioinformatics.nih.gov/) and analysis of protein–protein interaction in STRING (https://string-db.org/). Graphs of the transcriptome analysis results were produced using Science and Research Online Plots (https://www.bioinformatics.com.cn/srplot). *P* values less than or equal to 0.05 were considered statistically significant between conditions. All data are expressed as mean ± standard error of the mean (s.e.m.).

## Results

### CRS induces depressive-like behavior and reduces MeCP2 only in D2R neurons in the NAc

**Fig. 1:**
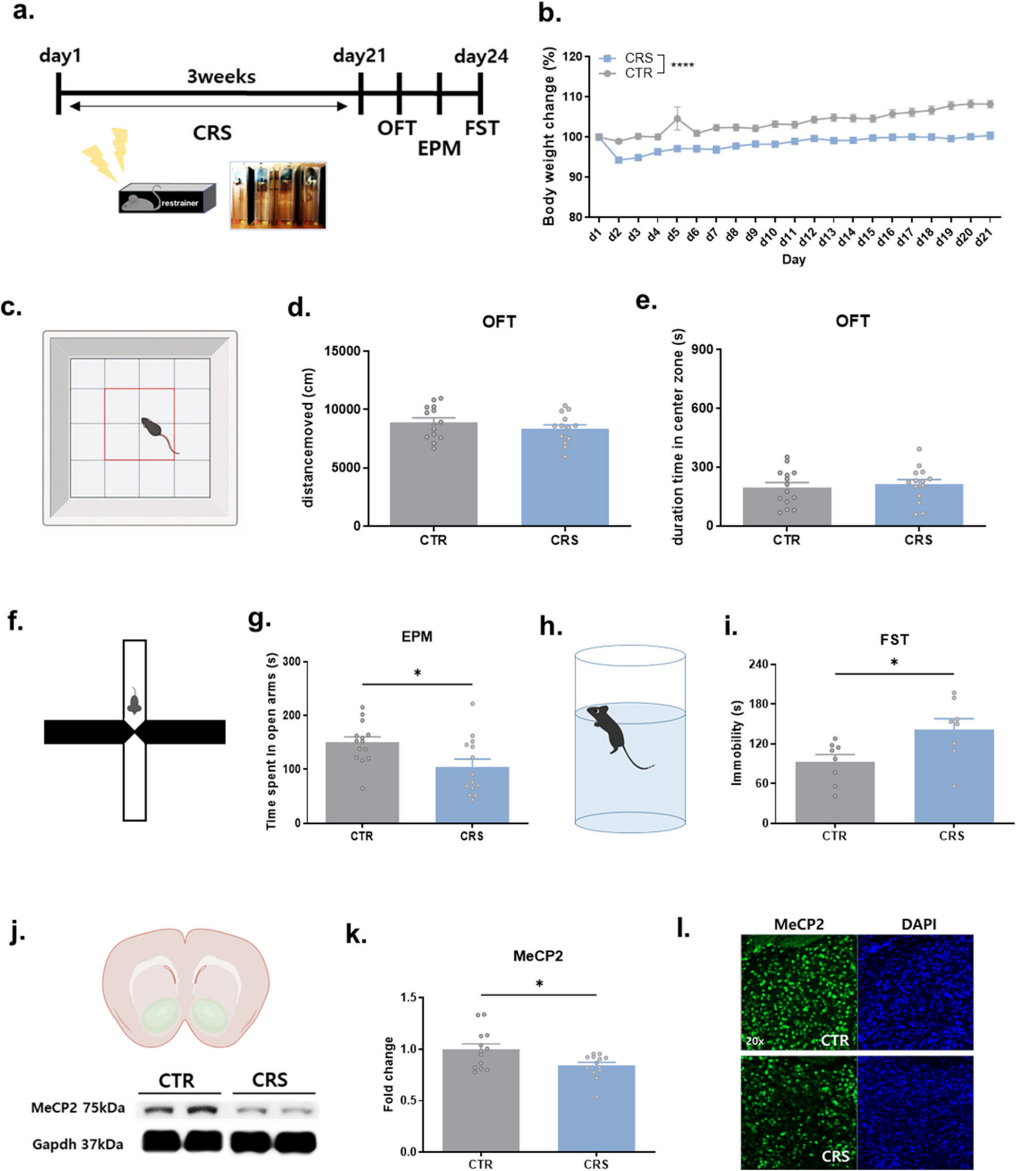
CRS induces depression-like behaviors and reduces MeCP2 expression selectively in NAc D2R neurons. **a** Schematic timeline of behavioral testing following 3-week CRS exposure. OFT, open field test. **b** CRS progressively reduced body weight during the exposure period (two-way mixed ANOVA, *F*(20,600) = 4.330, *P* < 0.0001; *n* = 16 per group). **c**–**e** Locomotor activity and exploratory behavior in the OFT were not altered by CRS **c** Schematic of the OFT arena and center zone used for analysis. **d** Total distance moved and **e** time spent in the center zone (distance moved: *t* = 1.111, *P* = 0.2767; center time: *t* = 0.4432, *P* = 0.6613; *n* = 14 per group). **f**, **g** CRS increased anxiety-like behavior in the EPM. **f** Schematic of the elevated plus maze (EPM) used for analysis. **g** Time spent in the open arms was reduced by CRS (*t* = 2.561, *P* = 0.0166; *n* = 14 per group). **h**, **i** CRS increased immobility in the FST, indicating depressive-like behavior (*t* = 2.498, *P* = 0.0256; *n* = 8 per group). **j**, **k** Western blot analysis revealed decreased MeCP2 protein levels in the NAc following CRS. **j** Representative immunoblots of MeCP2 and GAPDH (loading control) from control (CTR) and CRS groups. **k** Densitometric quantification of MeCP2 normalized to GAPDH (fold change relative to CTR) (*t* = 2.537, *P* = 0.0181; *n* = 13 per group). **l** Representative immunofluorescence images confirm reduced MeCP2 expression in the NAc after CRS (20×).

To confirm the association between depressive-like behaviors and MeCP2 in the NAc, we first exposed C57BL/6J male mice to CRS (Fig. 1a). Except for immediately before CRS exposure (day 1), CRS group mice showed decreases in body weight compared to the control group on all other days (Fig. 1b). CRS did not change the distance traveled by mice in an open field test (Fig. 1c, d), indicating that it did not impair the general motor function of mice. CRS also did not decrease the time spent in the center zone of an open field box, a measure of anxiety level (Fig. 1e), but decreased the time spent in the open arm of an EPM (Fig. 1f, g). In addition, it increased immobility in a FST (Fig. 1h, i), indicating that CRS induced anxiety-like and depressive-like behaviors.

We investigated whether CRS exposure changes the expression level of MeCP2 protein in the NAc. We observed a marked decrease in the expression level of MeCP2 protein in the NAc of mice collected 3 days after the last CRS exposure compared with the control group (Fig. 1j–l). The expression of MeCP2 was not changed in the dorsal striatum, medial prefrontal cortex or hippocampus, which are involved in motivational and emotional regulation (Supplementary Fig. 1). The same decrease in MeCP2 was observed at 2 h after the last CRS (Supplementary Fig. 2).

**Fig. 2:**
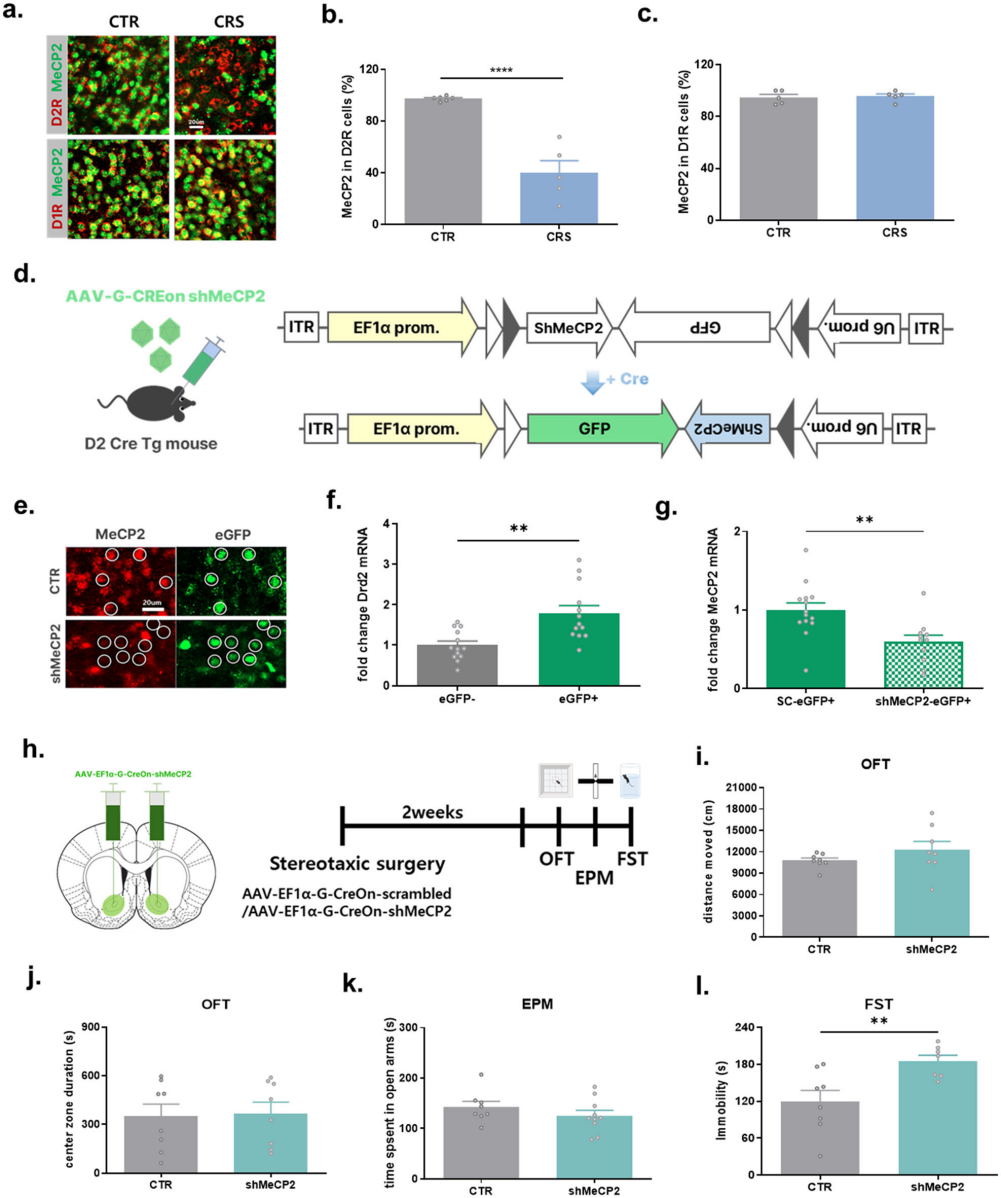
MeCP2 reduction selectively in NAc D2R neurons mediates CRS-induced depressive behavior. **a**–**c** CRS selectively reduces MeCP2 expression in D2R neurons: representative immunofluorescence images of MeCP2 with D1R or D2R markers in NAc collected 3 days after CRS (scale bar, 20 µm) (**a**); quantification shows a reduction of MeCP2-positive D2R neurons after CRS (*t* = 6.741, *P* < 0.0001; *n* = 6 (NC), 5 (CRS)) (**b**); MeCP2 expression in D1R neurons was unchanged (*t* = 0.3253, *P* = 0.7533; *n* = 5 per group) (**c**). **d**–**g** AAV-G-CREon-shMeCP2 selectively suppresses MeCP2 in D2R neurons: schematic of Cre-dependent shMeCP2 viral construct (**d**); representative MeCP2 immunostaining confirming MeCP2 reduction in GFP-positive neurons (scrambled-shRNA control versus shMeCP2) (**e**); GFP-positive cells showed enriched *Drd2* expression (paired *t*-test, *t* = 3.781, *P* = 0.0026; *n* = 13) (**f**); *MeCP2* mRNA was decreased in GFP-positive neurons expressing shMeCP2 compared with scrambled controls (*t* = 3.136, *P* = 0.0046; *n* = 14, 11) (**g**). **h**–**l** Cell-type-specific MeCP2 knockdown in D2R neurons increases depressive-like behavior: timeline of stereotaxic viral injection and behavioral testing (**h**); shMeCP2 did not affect locomotion (**i**, *t* = 1.219, *P* = 0.2429), exploratory behavior (**j** *t* = 0.1585, *P* = 0.8763) or anxiety-like behavior in EPM (**k** *t* = 1.131, *P* = 0.2747); shMeCP2 increased immobility in the FST (*t* = 3.073, *P* = 0.0089; *n* = 8, 7), indicating enhanced depressive-like behavior (**l**). Data shown as mean ± s.e.m. *P < 0.05, **P < 0.01, ***P < 0.001, ****P < 0.0001.

Recent studies have reported that D1R and D2R neurons, two types of cells that make up the majority of NAc neurons, regulate depressive symptoms differently and that related genes are changed differently in each cell type in depression models. Therefore, we examined whether the expression level of MeCP2 in the NAc by CRS shows different patterns depending on cell type. We first distinguished the two cells using antibodies reactive to D1 and D2 receptors (Supplementary Fig. 3a,b) and confirmed the expression changes in each cell type in the NAc of CRS-exposed mice. Interestingly, CRS exposure reduced MeCP2 in D2R neurons but not in D1R neurons (Fig. 2a–c and Supplementary Fig. 4a, b). This difference in expression was the same 2 h after the end of CRS (Supplementary Fig. 5a–e).

### MeCP2 regulates depressive phenotypes in a cell-type-specific manner in the NAc

To investigate the association between the reduction of MeCP2 in D2R neurons in the NAc and the induction of depressive symptoms, we constructed cell-type-specific AAV vectors expressing shMeCP2 only in D2 neurons (Fig. 2d–g). AAV-G-CREon-scrambled or AAV-G-CREon-shMeCP2 was injected bilaterally into the NAc of D2Cre mice. The reduction of MeCP2 protein and mRNA levels was confirmed by immunohistochemistry and qPCR from the cells isolated by FACS (Fig. 2e–g and Supplementary Fig. 6).

We confirmed that MeCP2 knockdown in D2R neurons without exposure to CRS could induce depressive-like symptoms (Fig. 2h–k). shMeCP2 mice showed increased immobility in a FST compared with the control group. These data indicate that reducing MeCP2 in NAc D2R neurons is sufficient to increase immobility in the FST, consistent with a contributory role of MeCP2 loss to stress-related behavioral outcomes (Fig. 2l). By contrast, MeCP2 knockdown in D2R neurons did not affect locomotion or anxiety levels (Fig. 2i–k).

**Fig. 3:**
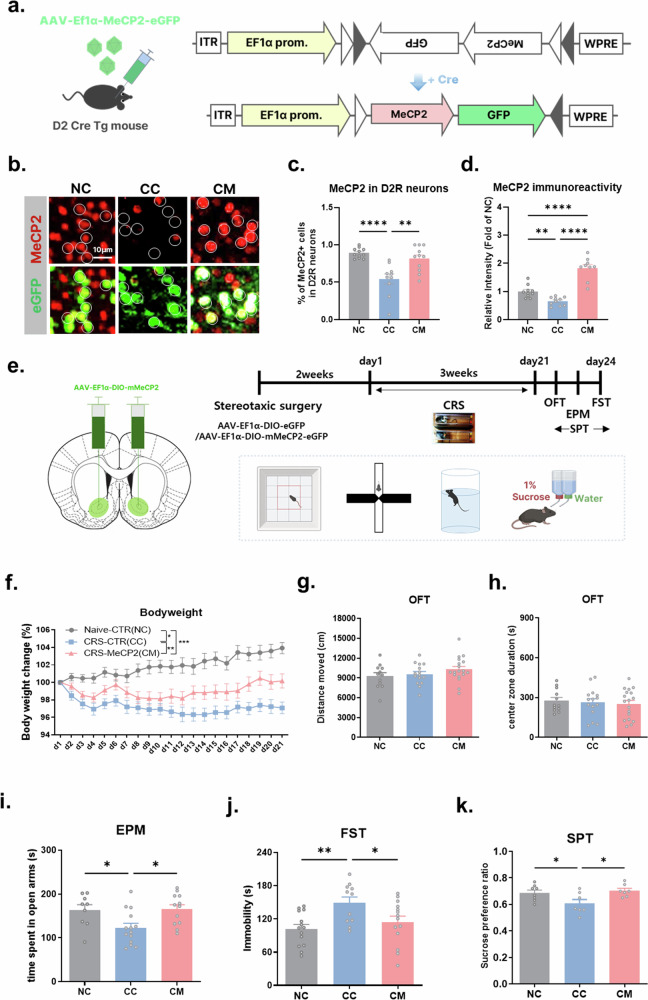
Genetic enhancement of MeCP2 in NAc D2R neurons reverses CRS-induced depressive-like behavior. **a**–**d** Cre-dependent viral strategy and validation of MeCP2 upregulation in NAc D2R neurons: schematic of the AAV-Ef1α-DIO-MeCP2-eGFP construct (**a**); representative immunofluorescence images showing MeCP2 immunoreactivity (red) in eGFP-labeled D2R neurons (green) across NC, CC and CM groups (scale bar, 20 µm) (**b**); quantification of the proportion of MeCP2-positive cells among eGFP-labeled D2R neurons (one-way ANOVA: *F* = 13.48, *P* < 0.0001; Holm–Šídák post-hoc: NC versus CC *P* < 0.0001; CC versus CM *P* = 0.0011) (**c**); quantification of MeCP2 immunoreactivity intensity within eGFP-labeled D2R neurons, expressed as fold change relative to NC (one-way ANOVA with Holm–Šídák post-hoc: NC versus CC *P* = 0.0090; NC versus CM *P* < 0.0001; CC versus CM *P* < 0.0001) (**d**). **e** Experimental timeline for CRS and behavioral testing. **f** Body-weight tracking during CRS. CRS reduced body weight in the CC group, and MeCP2 overexpression partially rescued this reduction (two-way mixed ANOVA; *n* = 13/19/19 for NC/CC/CM; post-hoc Holm–Šídák: NC versus CC *P* < 0.001, NC versus CM *P* = 0.003, CC versus CM *P* = 0.019). **g**–**k** Behavioral outcomes following MeCP2 upregulation: no differences were observed in the OFT, including total distance moved (locomotor activity) (**g**) and time spent in the center zone (center exploration) (**h**) (one-way ANOVA, ns; *n* = 12/15/18 for NC/CC/CM); anxiety-like behavior was elevated by CRS and normalized by MeCP2 overexpression in the EPM (one-way ANOVA; *n* = 9/14/12 for NC/CC/CM; *F*(2,32) = 5.241, *P* = 0.0107; post-hoc: NC versus CC *P* = 0.0320, NC versus CM *P* = 0.9036, CC versus CM *P* = 0.0201) (**i**); MeCP2 overexpression reduced CRS-induced immobility in the FST (one-way ANOVA; *n* = 14/11/14 for NC/CC/CM; *F*(2,36) = 5.570, *P* = 0.0078; post-hoc: NC versus CC *P* = 0.0072, CC versus CM *P* = 0.0434) (**j**); sucrose preference ratio in the SPT (one-way ANOVA; *n* = 8/7/7 for NC/CC/CM; *F*(2,19) = 4.767, *P* = 0.0210; post-hoc: NC versus CC *P* = 0.0473, NC versus CM *P* = 0.6141, CC versus CM *P* = 0.0290) (**k**). Data represent mean ± s.e.m. *P < 0.05, **P < 0.01, ***P < 0.001, ****P < 0.0001.

Next, we asked whether restoring MeCP2 selectively in NAc D2R neurons is sufficient to mitigate CRS-induced behavioral alterations. To this end, we generated a Cre-dependent AAV vector enabling D2R neuron-restricted MeCP2 expression (Fig. 3a). Immunohistochemical analyses confirmed successful MeCP2 restoration in GFP-labeled D2 neurons, showing an increased proportion of MeCP2-positive cells and elevated nuclear MeCP2 intensity compared with CRS controls (Fig. 3b–d and Supplementary Fig. 7).

AAV-EF1α-DIO-eGFP (control) or AAV-EF1α-DIO-mMeCP2-eGFP was bilaterally injected into the NAc of D2-Cre mice, followed by a three-week CRS paradigm initiated 2 weeks after surgery (Fig. 3e). Compared with CRS control mice, MeCP2 overexpression was associated with a partial normalization of body weight during the stress period, although values did not fully return to naive control levels (Fig. 3f). Importantly, MeCP2 restoration did not alter baseline locomotor activity or anxiety-like behavior in the open field test (Fig. 3g, h), indicating that subsequent behavioral effects were not attributable to nonspecific changes in activity.

In behavioral assays probing stress-related affective responses, MeCP2 overexpression attenuated CRS-induced alterations in the EPM and reduced immobility in the FST (Fig. 3i,j). In addition, to assess reward-related behavioral dimensions, we measured sucrose preference as an index of anhedonia. Consistent with prior studies, CRS reduced sucrose preference^18–20^, and this deficit was restored by MeCP2 overexpression in D2 neurons in the NAc (Fig. 3k). Together, these findings indicate that cell-type-specific restoration of MeCP2 in NAc D2 neurons is sufficient to ameliorate multiple CRS-associated behavioral phenotypes spanning affective coping and reward-related domains.

Upregulation of MeCP2 in D2R neurons did not affect locomotion or center-zone time in the open field test in CRS-exposed mice (Fig. 3g, h). In addition, MeCP2 overexpression in NAc D2R neurons under nonstressed conditions did not produce overt cognitive or motor impairments in the assays performed (Supplementary Fig. 8), supporting that D2R-restricted MeCP2 upregulation does not grossly disrupt general function under these conditions.

We also tested whether MeCP2 overexpression in NAc D1R neurons modulates CRS-induced behavioral alterations. In our dataset, MeCP2 upregulation in D1R neurons did not attenuate CRS-associated depressive-like phenotypes (Supplementary Fig. 9), consistent with a D2R neuron-biased effect in this paradigm. Together, these results support that repeated and persistent stress reduces MeCP2 protein selectively in NAc D2R neurons and that restoring MeCP2 in this cell population is sufficient to ameliorate multiple CRS-associated behavioral phenotypes.

### MeCP2 overexpression in D2R neurons restores normal neural activity suppressed by CRS

**Fig. 4:**
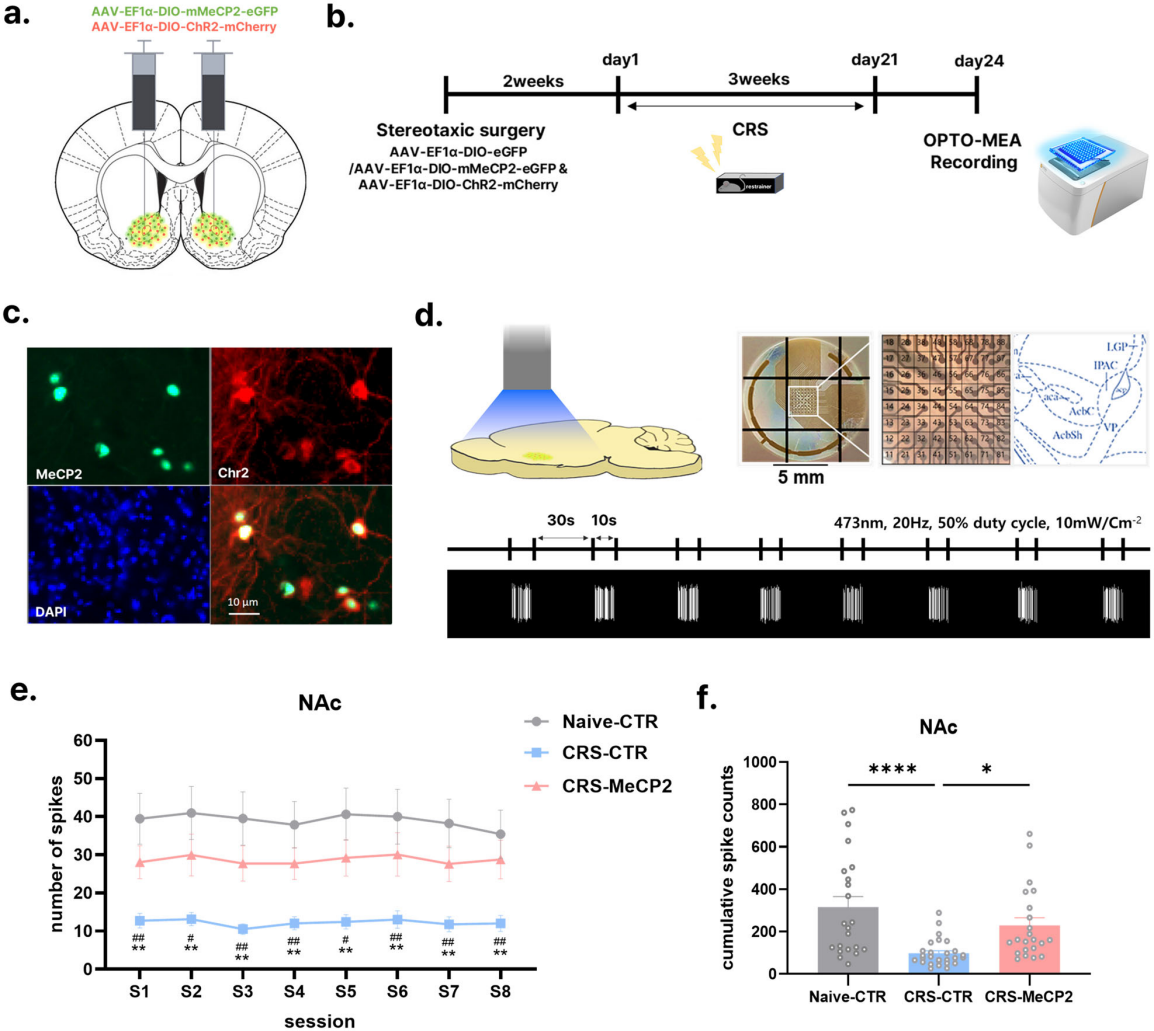
Genetic enhancement of MeCP2 restores neural activity patterns in NAc D2R neurons under stress. **a**–**c** Viral approach and validation: schematic of Cre-dependent coexpression of MeCP2 and ChR2 in NAc D2R neurons (**a**); experimental timeline for optogenetic stimulation and slice electrophysiology in the CRS model (**b**); representative immunofluorescence images confirming coexpression of MeCP2 (green) and ChR2 (red) in D2R neurons (scale bar, 10 µm) (**c**). **d**–**f** Optogenetically evoked firing responses recorded using a MEA: sagittal NAc slices were positioned on a 64-channel MEA, and blue-light pulses (473 nm; stimulation paradigm repeated across 8 cycles/sessions; ‘Materials and methods’) were delivered to activate ChR2-expressing neurons (**d**); CRS reduced optogenetically evoked firing, while MeCP2 augmentation partially restored activity (that is, shifted responses toward NC levels) across stimulation sessions (two-way mixed ANOVA, *F*(2, 68) = 10.47, *P* = 0.0001; Holm–Šídák post-hoc tests: NC versus CC *P* < 0.01, CC versus CM *P* < 0.05-0.01 across sessions) (**e**); cumulative spike counts across all sessions revealed a persistent reduction in neural activity in CRS mice, which was attenuated by MeCP2 overexpression (shifted toward NC levels) (one-way ANOVA, *F*(2, 68) = 10.56, *P* = 0.0001; post-hoc: NC versus CC ****P < 0.0001, CC versus CM *P = 0.0156) (**f**). Data are shown as mean ± s.e.m. *P < 0.05, **P < 0.01, ****P < 0.0001.

Our findings—particularly the marked reduction in MeCP2 in D2R neurons of the nucleus accumbens following CRS exposure and the changes in depressive-like behaviors observed when MeCP2 expression was manipulated—demonstrate the functional importance of MeCP2 in depression. Therefore, we investigated the neurophysiological mechanism by which MeCP2 in D2R neurons exerts therapeutic efficacy. To elucidate the neurological mechanism underlying MeCP2 regulation of depression in D2R neurons of the NAc, we first investigated whether genetic manipulation of MeCP2 induces changes in neuronal activity in depression. Changes of neuronal activity in the brain are known to be a representative symptom of depression^21,22^. Based on recent reports on the function of MeCP2 in regulating neuronal activity^8,23^, we hypothesized that neuronal activity in the NAc would be dysregulated in the CRS model, in which MeCP2 levels are decreased. We also examined whether MeCP2 upregulation could normalize CRS-induced dysregulation of neural activity. To confirm this, we measured electrophysiological responses in ex vivo NAc slices using a MEA recording system and optogenetics. We co-expressed MeCP2 and channelrhodopsin in D2R neurons of the NAc by injecting AAV viruses to enable Cre-dependent coexpression of MeCP2 and ChR2 into D2R Cre mice (Fig. 4a–c and Supplementary Fig. 10a). In response to optical stimulation (blue-light stimulation repeated across eight cycles/sessions; see Fig. 4d, e and the ‘Materials and methods’ for details), the CRS group showed reduced neural activity in the NAc compared with the control group. This suggests that CRS exposure changed the neural activity level of D2R neurons (Fig. 4b, d–f and Supplementary Fig. 10b). Across eight repeated sessions, the neuronal responses to optical stimulation remained stable without any systematic increase or decrease (Fig. 4e). Overexpression of MeCP2 attenuated the CRS-induced dysregulation of neural activity in the NAc.

### Spatial transcriptomic profiling of NAc D2R neurons and the downstream VP circuit

**Fig. 5:**
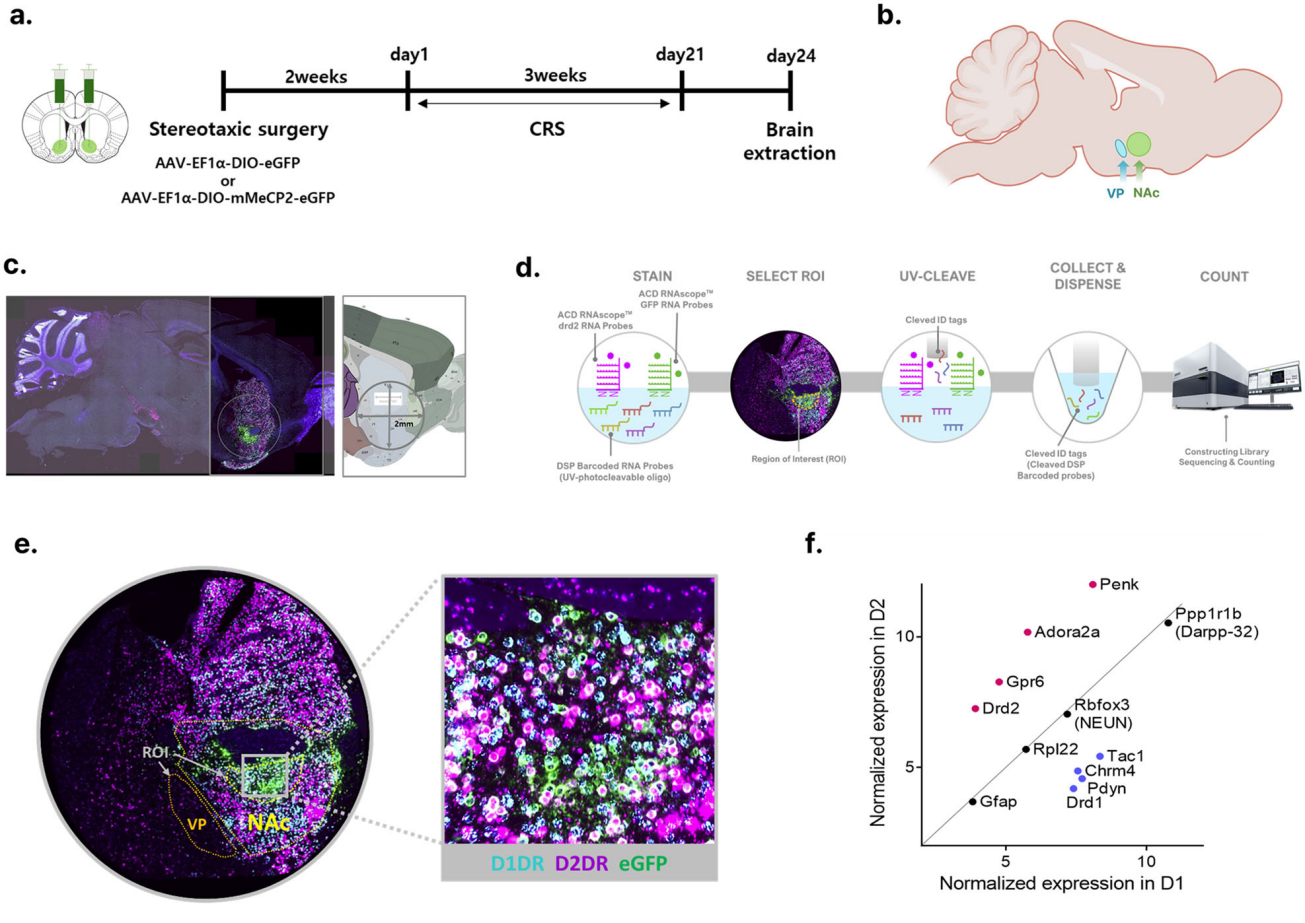
Workflow and validation of cell-type-specific spatial transcriptomics using GeoMx DSP. **a** Schematic of the experimental design. Cre-dependent AAV expressing MeCP2 or *eGFP* was bilaterally delivered into the NAc of D2R-Cre mice. Mice underwent CRS for 3 weeks before tissue collection. Naive controls (NC) received the same viral delivery but were not exposed to CRS. **b** Sagittal brain section illustrating sampled regions for GeoMx DSP, including the NAc and the VP, a major downstream target of D2R neurons. **c** Representative FISH images showing D1R-, D2R-expressing neurons and virally labeled cells (*eGFP*). Only *Drd2*^+^/*eGFP*^+^ cells within the defined 2-mm ROI were selected for transcriptomic profiling. **d** Workflow of GeoMx DSP. Cell identity was determined using FISH probes targeting *Drd1*, *Drd2* and *eGFP*. UV-cleavable molecular barcodes corresponding to target transcripts were released selectively from the defined ROI and quantified by next-generation sequencing. **e** Higher-magnification FISH images demonstrating ROI placement and cellular resolution of analyzed regions. **f** Validation of cell-type assignment. Normalized expression of canonical D1R and D2R markers confirmed accurate molecular discrimination of neuronal subpopulations in the NAc (D2R ROIs from *n* = 9 mice; D1R ROIs from *n* = 8 mice).

To investigate how MeCP2 in NAc D2R neurons regulates the molecular pathways underlying depressive behavior, we performed spatial transcriptomic profiling using the GeoMx DSP platform (Fig. 5a, d). D2R-expressing neurons in the NAc were selectively transduced with AAV vectors expressing MeCP2 together with eGFP or eGFP alone, establishing three experimental groups: naive control (NC; eGFP injected without CRS exposure), CRS-exposed control (CC; eGFP injected with CRS) and CRS-exposed MeCP2-overexpression (CM; MeCP2-eGFP injected with CRS). Brain tissues were collected 3 days following the final stress session to capture stable transcriptional adaptations (Fig. 5a).

To ensure precise cell-type specificity, fluorescence in situ hybridization (FISH) targeting *Drd1*, *Drd2* and *eGFP* mRNAs was used to define ROIs within sagittal sections (Fig. 5c, e). The explicit co-localization of *eGFP* with *Drd2* confirmed that viral expression was restricted to D2R neurons, validating our cell-type-specific targeting strategy (Supplementary Fig. 11). Before differential expression analysis, the accuracy of ROI segmentation was verified by evaluating canonical subtype markers, which demonstrated a robust separation between D1R and D2R neuronal populations^24,25^ (Fig. 5f, Supplementary Fig. 12 and Supplementary Table 1). Consistent with canonical signaling pathways of striatal MSNs^26,27^, D1R-enriched transcripts were associated with G-protein coupled receptor signaling and protein kinase activity, reflecting the Gs/olf-cAMP-PKA signaling cascade characteristic of D1 receptors (Supplementary Tables 2–4). Conversely, D2R-enriched transcripts were enriched in adenosine metabolic processes, aligning with the established exclusive expression of adenosine A2A receptors (*Adora2a*) in D2R-expressing neurons^28,29^. This functional segregation validates the biological accuracy of our cell-type specific profiling (Supplementary Fig. 13).

Given that the VP serves as the primary downstream projection target of NAc D2R neurons and receives substantial inhibitory input from this pathway (Fig. 5b), we hypothesized that the transcriptional consequences of CRS and MeCP2 regulation in the NAc would be accompanied by parallel molecular signatures in this circuit node. Consequently, we extended our analysis to the VP to examine whether CRS-responsive molecular signatures in this region are associated with NAc D2R-restricted MeCP2 upregulation.

### MeCP2 attenuates CRS-induced transcriptional dysregulation in NAc D2R neurons

To determine how MeCP2 modulates stress-responsive transcriptional programs, we performed spatial transcriptomic profiling of eGFP-labeled D2R neurons using GeoMx DSP across naive controls (NC), CRS-exposed controls (CC) and CRS-exposed mice overexpressing MeCP2 (CM). Consistent with successful viral transduction, *MeCP2* transcripts were elevated in the CM group (Supplementary Fig. 14). By contrast, NC and CC groups displayed comparable *MeCP2* mRNA expression levels despite the marked reduction in MeCP2 protein observed in the CC group. This dissociation is consistent with regulation beyond transcription (for example, post-transcriptional and/or post-translational processes) rather than transcriptional repression, although the present data do not distinguish among altered translation, protein stability or degradation mechanisms.

**Fig. 6:**
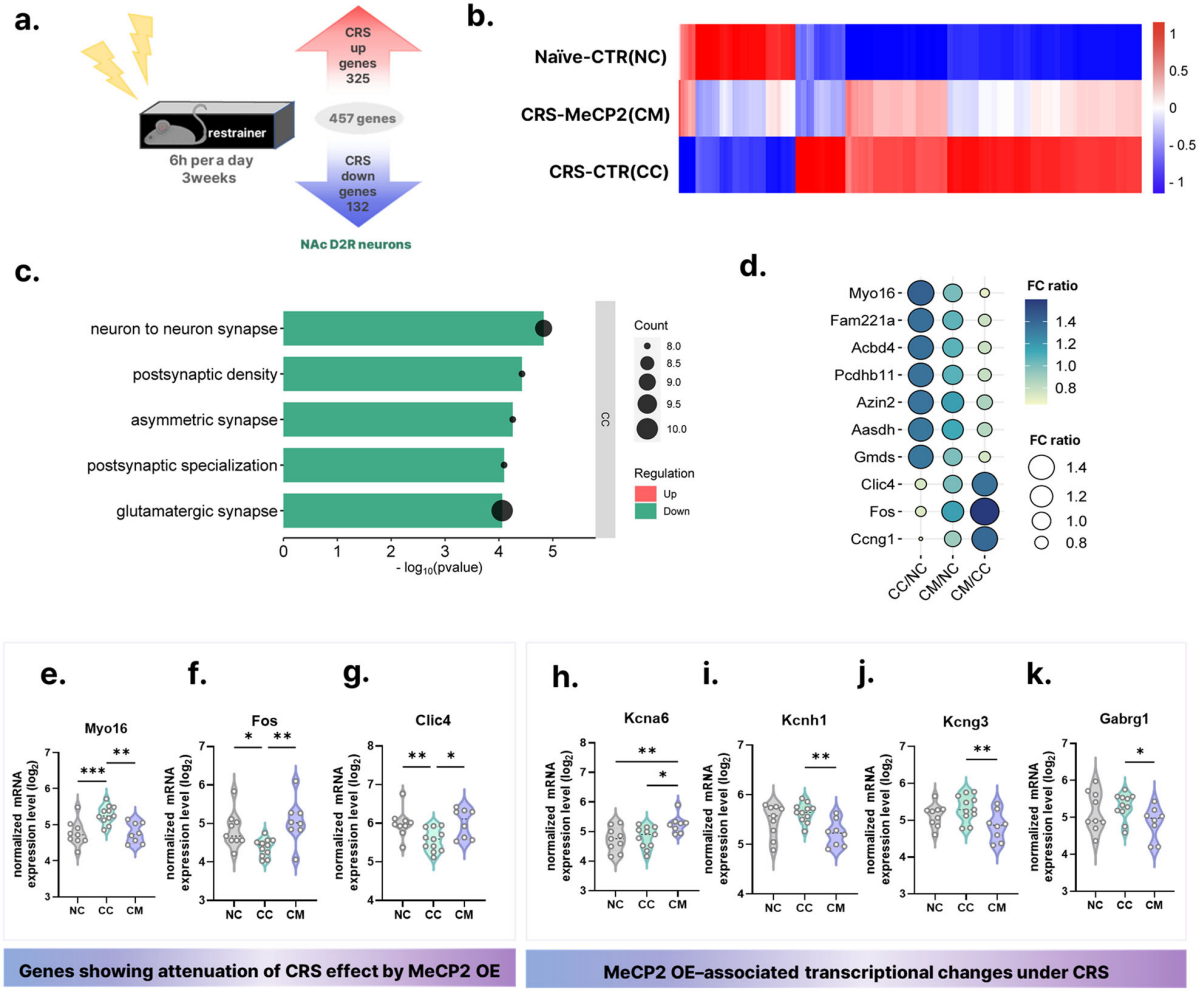
Differential gene expression and MeCP2-associated transcriptional modulation in NAc D2R neurons revealed by GeoMx DSP. Comparison groups: NC (control AAV), CC (CRS + control AAV), CM (CRS + AAV-MeCP2). Sample sizes: *n* = 9 (NC), 11 (CC), 8 (CM). **a** A total of 457 transcripts in NAc D2R neurons were identified as CRS-responsive (|FC| >1.5 (|log₂FC| >0.58), nominal *P* < 0.05). **b** Heatmap visualizing relative expression patterns across the three groups (*Z*-scored normalized expression) **c** GO enrichment (Cellular Component) among CRS-responsive transcripts whose CRS-associated group differences (CC versus NC) were attenuated in CM (false discovery rate (FDR) *q* < 0.05 for GO terms). Direction of CRS effect was defined as CC versus NC (Up/Down). Enriched terms meeting the stated FDR threshold were detected only in the CRS-downregulated subset under the stated criteria (FDR-adjusted *P* < 0.05), whereas no terms met the same threshold for the CRS-upregulated subset. The *x* axis represents fold enrichment. **d** Bubble plot highlighting the top ten transcripts exhibiting the largest CRS-associated expression shifts and reduced group-level differences following MeCP2 overexpression (CC/NC|FC| >1.3, nominal *P* < 0.05; CM/NC nominal *P* > 0.30; CM/CC *P* < 0.05). For visualization, both bubble color and bubble size encode FC (ratio); FC <1 indicates decreased expression and FC >1 indicates increased expression. **e**–**g** Violin plots showing three neuronal activity- and plasticity-related transcripts (*Myo16*, *Fos* and *Clic4*) from the top-10 subset, illustrating cases where CRS-associated expression differences were reduced following MeCP2 overexpression. Statistical analysis: one-way ANOVA with Fisher’s post-hoc test (**e**: *Myo16*, *F*(2,25) = 10.06, *P* = 0.0006; NC versus CC *P* = 0.0007, NC versus CM *P* = 0.9612, CC versus CM *P* = 0.0010; **f** *Fos*, *F*(2,25) = 6.297, *P* = 0.0061; NC versus CC *P* = 0.0253, NC versus CM *P* = 0.3005, CC versus CM *P* = 0.0513. **g** *Clic4*, *F*(2,25) = 5.356, *P* = 0.0116; NC versus CC *P* = 0.0099, NC versus CM *P* = 0.9632, CC versus CM *P* = 0.0109). Data shown as mean ± s.e.m. **h**–**k** Violin plots showing representative potassium channel-related transcripts selected from the subset of 100 genes demonstrating significant MeCP2-dependent modulation under CRS. Selection was based on functional enrichment analysis (DAVID clustering) identifying ion-channel-associated gene categories. Statistical analysis: one-way ANOVA with Fisher’s post-hoc test (**h**: *Kcna6*, *F*(2,25) = 4.935, *P* = 0.0156; NC versus CC *P* = 0.6784, NC versus CM *P* = 0.0078, CC versus CM *P* = 0.0147. **i**: *Kcnh1*, *F*(2,25) = 5.799, *P* = 0.0085; NC versus CC *P* = 0.1818, NC versus CM *P* = 0.0583, CC versus CM *P* = 0.0022. **j**: *Kcng3*, *F*(2,25) = 4.432, *P* = 0.0225; NC versus CC *P* = 0.2355, NC versus CM *P* = 0.0975, CC versus CM *P* = 0.0064. **k** *Gabrg1*, *F*(2,25) = 2.294, *P* = 0.1217; NC versus CC *P* = 0.5166, NC versus CM *P* = 0.1678, CC versus CM *P* = 0.0439). Data presented as mean ± s.e.m. *P < 0.05, **P < 0.01, ***P < 0.001, ****P < 0.0001.

CRS induced broad transcriptional remodeling in D2R neurons. To comprehensively capture the landscape of stress-induced molecular changes in this specific cell type, we identified 457 CRS-responsive genes (325 upregulated, 132 downregulated) using a nominal *P*-value threshold (|FC| > 1.5, nominal *P* < 0.05; Fig. 6a, Supplementary Table 5), where |FC| denotes fold-change magnitude (|FC| = max(FC, 1/FC); equivalently |log_2_FC| >0.58). Functional annotation revealed that these genes were strongly enriched for synaptic structure and glutamatergic signaling components, including ‘postsynaptic density’ and ‘neuron-to-neuron synapse’ (Supplementary Fig. 15a,b and Supplementary Tables 6–8). Complementary annotation-based enrichment analyses suggested that the CRS-responsive gene set was also associated with cytoskeletal organization and metabolic processes, including palmitoylation and peroxisome-related terms (Supplementary Fig. 15c–e and Supplementary Tables 9–11).

We next examined whether MeCP2 overexpression modulated CRS-induced transcriptional profiles in NAc D2R neurons. We identified 250 genes for which MeCP2 overexpression attenuated CRS-associated expression shifts, defined by a CRS-associated change under stress (CC versus NC: |FC| >1.3, nominal *P* < 0.05) followed by a return to levels statistically indistinguishable from baseline controls in the MeCP2 overexpression group (CM versus NC: *P* > 0.10; Fig. 6b and Supplementary Table 12). While this criterion does not imply equivalence, it indicates a substantial shift of expression profiles toward baseline.

In GO enrichment analysis of the Cellular Component (CC) category, functional terms meeting the stated FDR threshold were detected for the CRS-downregulated attenuated subset (Direction of CRS effect, CC versus NC: down), with top categories including ‘postsynaptic membrane’, ‘synapse organization’ and ‘glutamatergic synapse’ (Fig. 6c and Supplementary Tables 13–15). By contrast, no terms met the same threshold for the CRS-upregulated attenuated subset under the same criteria. In parallel, complementary functional annotation clustering analyses (DAVID) suggested that CRS-upregulated attenuated genes were associated with categories linked to neuronal structure and signaling (for example, protein palmitoylation, cilium-related annotations and actin filament binding), whereas CRS-downregulated attenuated genes showed stronger convergence on synaptic and glutamatergic programs; these broader annotation patterns are provided in the supplementary analyses (Supplementary Fig. 16 and Supplementary Tables 16–18). Together, these enrichment and annotation analyses highlight synaptic and neuronal signaling programs as prominent features of the attenuation-defined gene set and guided our selection of representative examples presented in the main figure.

To identify representative recovery-associated candidates within the attenuation-defined set, we prioritized genes showing robust restoration patterns (CC versus NC: |FC| >1.3, nominal *P* < 0.05; CM versus CC: *P* < 0.05) and selected the top ten with the greatest convergence toward baseline levels (CM versus NC) (Fig. 6d). *Myo16*, *Fos* and *Clic4* were highlighted as representative examples spanning synaptic structural regulation, activity-dependent transcription and ion/stress homeostasis, respectively (Fig. 6e–g). Notably, restoration of *Fos* expression (Fig. 6f) is consistent with the optogenetic spike recording results (Fig. 4), providing a transcript-level correlate of restored neuronal activity.

As complementary analyses, we summarized MeCP2-associated changes in gene families related to neuronal communication^30^ (ion channels and neurotransmitter receptors; Supplementary Fig. 17 and Supplementary Tables 19–25), and also examined MeCP2-associated differences under CRS using a direct comparison between CM and CC groups (CM versus CC; Fig. 6h–k, Supplementary Fig. 18 and 19 and Supplementary Tables 26–29). Together, these focused transcript-level signatures provide molecular context for the electrophysiological normalization observed in CM mice (Fig. 4), by highlighting coordinated changes in gene programs annotated to neuronal communication under MeCP2 overexpression.

Although *Bdnf* transcripts were not detected in our GeoMx DSP ROIs from virally labeled NAc D2R neurons, extensive prior studies have established BDNF as a key regulator of depression-related phenotypes and neuronal plasticity^7,8,31,32^; therefore, we performed complementary protein-level analyses. Bulk NAc ELISA revealed a CRS-associated increase in BDNF protein that was attenuated by NAc D2R-restricted MeCP2 overexpression (Supplementary Fig. 20a), although the cellular source of this signal cannot be resolved by bulk measurements. In parallel, BDNF immunoreactivity within eGFP-labeled D2R neuronal somata was low/sparse and did not show an apparent group difference across conditions (Supplementary Fig. 20b–e). Given that BDNF-mediated plasticity is closely linked to activity-dependent signaling pathways, we additionally assessed CREB/pCREB (Supplementary Fig. 21) and phospho-MeCP2 (pMeCP2; Supplementary Fig. 22) as complementary signaling correlates.

Collectively, these analyses indicate that CRS disrupts transcriptional programs in NAc D2R neurons and that MeCP2 overexpression is associated with attenuation of stress-linked perturbations, particularly within gene categories related to synaptic organization and neuronal communication.

### MeCP2 modulation in NAc D2R neurons is associated with CRS-responsive molecular signatures in the downstream VP

**Fig. 7:**
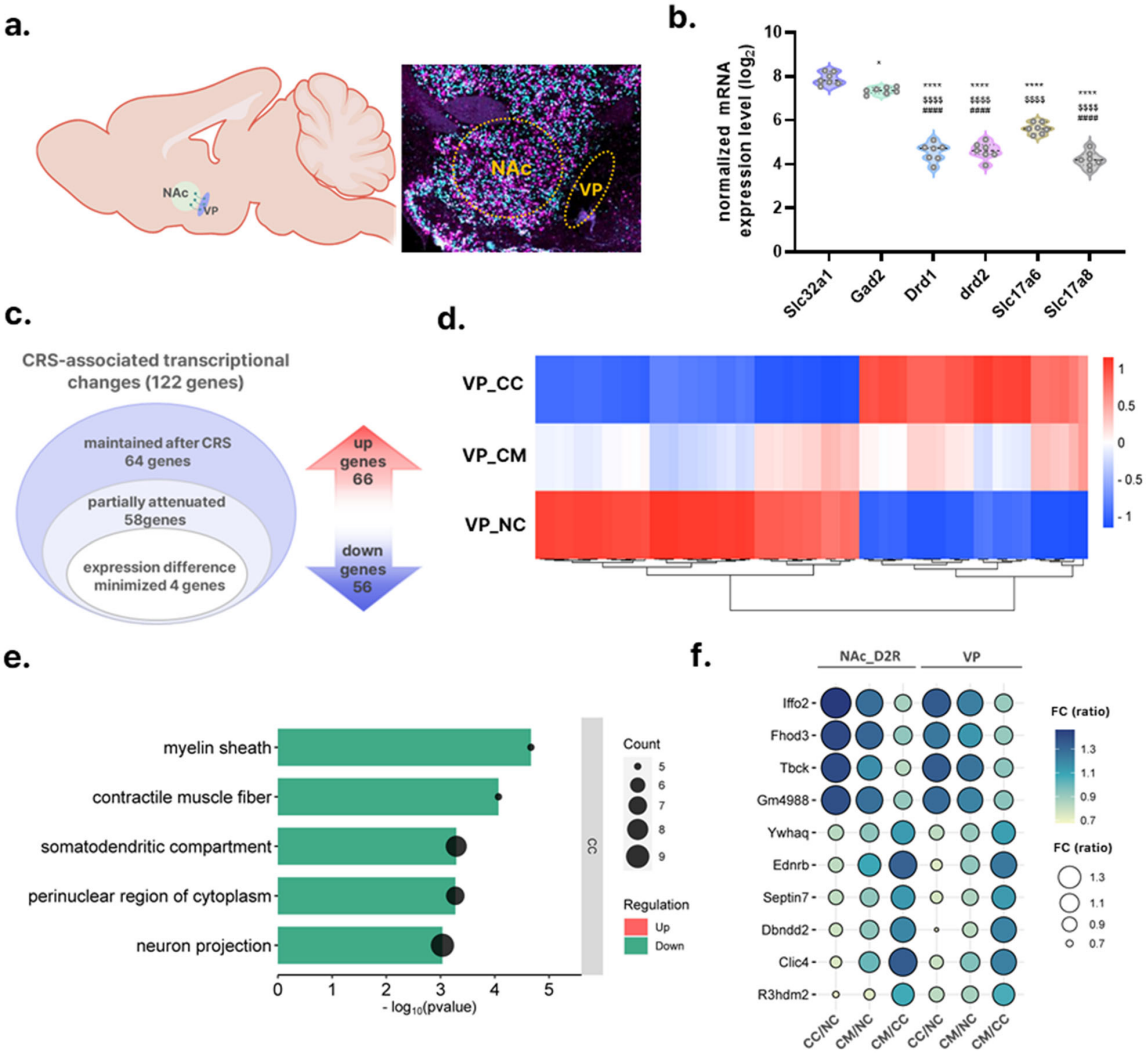
Spatial transcriptomic profiling identifies CRS-responsive molecular signatures in the VP and MeCP2-associated modulation following manipulation in NAc D2R neurons. Sample sizes: *n* = 7 (NC), *n* = 6 (CC) and *n* = 5 (CM). **a** Schematic of the anatomical relationship between the NAc and VP (left) and representative RNAscope images depicting the VP region selected for GeoMx DSP analysis (right). **b** Expression levels of canonical VP cell-type marker transcripts in the NC group, confirming appropriate cellular identity. One-way ANOVA with Holm–Šídák correction: *Slc32a1* versus *Gad2* *P* = 0.0107; *Slc32a1* versus *Drd1*, *Drd2*, *Slc17a6*, *Slc17a8* *P* < 0.0001; *Gad2* versus *Drd1*, *Drd2*, *Slc17a6*, *Slc17a8* *P* < 0.0001; *Drd1* versus *Drd2* *P* = 0.7242; *Drd1* versus *Slc17a6* *P* < 0.0001; *Drd1* versus *Slc17a8* *P* = 0.1172; *Drd2* versus *Slc17a6* *P* < 0.0001; *Drd2* versus *Slc17a8* *P* = 0.0808; *Slc17a6* versus *Slc17a8* *P* < 0.0001 (*P < 0.05, ****P < 0.0001 versus *Slc32a1*; $$$$*P* < 0.0001 versus *Gad2*; ####*P* < 0.001 versus *Slc17a6*.) **c** CRS exposure altered 122 VP transcripts (66 increased, 56 decreased; CC versus NC, |FC| >1.3, nominal *P* < 0.05). Among these, 58 transcripts showed reduced group-level differences in the MeCP2-manipulated condition (CM versus NC, nominal *P* > 0.10). **d** Heatmap showing the 58 CRS-responsive transcripts with attenuated differences under MeCP2 overexpression (*Z*-scored normalized expression). **e** GO (Cellular Component) enrichment of the 58 attenuated transcripts (FDR *q* < 0.05). **f** Bubble plot of 10 transcripts exhibiting parallel CRS- and MeCP2-associated expression patterns in both NAc D2R ROIs and VP ROIs. Candidate transcripts were required to meet the same predefined ‘attenuation’ criteria in each region (CC versus NC: |FC| >1.2 and nominal *P* < 0.05; CM versus NC: nominal *P* > 0.10; CM versus CC: nominal *P* < 0.05) and were then summarized as a cross-region panel. Because VP ROIs are not projection-resolved and reflect bulk tissue composition, these results are presented as associative downstream signatures rather than evidence for a direct NAc→VP projection-specific mechanism. For visualization, both bubble color and bubble size encode FC (ratio); FC <1 indicates decreased expression, and FC >1 indicates increased expression.

Given that the VP serves as the primary downstream projection target of NAc D2R neurons^33,34^ (Fig. 7a), we hypothesized that the transcriptional consequences of MeCP2 regulation in the NAc might extend to this circuit node. To test this, we analyzed the transcriptomic profile of the VP using GeoMx DSP. We first validated the cellular composition of our ROIs, confirming that the sampled VP tissues were predominantly composed of GABAergic neurons (*Slc32a1* and *Gad2*), consistent with the known cytoarchitecture of the VP^35,36^ (Fig. 7b).

CRS exposure induced transcriptional remodeling in the VP, identified as 122 CRS-responsive genes (66 upregulated, 56 downregulated) based on a nominal *P*-value threshold (|FC| >1.3, nominal *P* < 0.05; Fig. 7c and Supplementary Tables 30–34). Notably, MeCP2 overexpression in the NAc was associated with a partial attenuation of these stress-induced expression shifts in a subset of 58 genes. These genes were characterized by a CRS-associated deviation in the CRS group followed by a return to levels that were statistically indistinguishable from baseline controls in the MeCP2 overexpression group (nominal *P* > 0.10 versus NC; Fig. 7d and Supplementary Tables 35–38). GO enrichment analysis revealed that these MeCP2-associated genes were linked to myelination (‘myelin sheath’) and cytoskeletal organization (‘contractile muscle fiber’) (Fig. 7e, Supplementary Fig. 23 and Supplementary Tables 31–33), indicating that VP transcriptomic signatures related to these categories shifted in parallel with NAc D2R-targeted MeCP2 upregulation under CRS.

We further examined individual molecular targets in the VP that showed pronounced MeCP2-associated changes. Using a refined criterion for candidate selection (CC versus NC: |FC| >1.2, nominal *P* < 0.05; CM versus CC: *P* < 0.05), we identified 23 genes, including *Sertad4*, *Fxyd6* and *Gja1*, which have been implicated in immune response regulation and ion homeostasis^37^ (Supplementary Fig. 24 and Supplementary Table 39). MeCP2 overexpression was also associated with a return of several plasticity-related genes toward baseline expression levels in the VP, such as the GABA receptor subunit *Gabra2* and the potassium channel modulator *Kctd18* (Supplementary Fig. 25). Given the inhibitory projection from NAc D2R neurons to the VP, these changes are compatible with circuit-level coupling, but the present data do not resolve whether they reflect direct projection-mediated effects or indirect network adaptations.

Finally, to explore coordinated molecular adaptations across the circuit, we screened for genes showing similar expression trends in both NAc D2R neurons and the VP. This cross-regional screen was performed independently in each region across the measured transcript set (that is, not restricted to the NAc top-candidate list), and we then examined the overlap of genes showing concordant CRS-associated changes together with MeCP2-associated attenuation in both regions. We identified 10 candidates, including *Clic4*, that exhibited concordant CRS-associated expression shifts and MeCP2-associated shifts toward baseline in both regions (Fig. 7f, Supplementary Fig. 26 and Supplementary Table 40). This cross-regional concordance indicates coordinated attenuation of CRS-associated transcriptional signatures in both the NAc and VP following MeCP2 restoration in NAc D2R neurons, without implying projection-defined causality.

## Discussion

In this study, we identified a cell-type-specific reduction of MeCP2 protein in D2R neurons of the NAc following CRS and demonstrated that restoring MeCP2 expression in this neuronal population was sufficient to reverse stress-induced behavioral abnormalities. CRS decreased MeCP2 levels selectively in NAc D2 neurons (Fig. 1) and induced depressive-like behaviors across multiple dimensions, including increased immobility in the FST, reflecting passive coping or behavioral despair, and reduced sucrose preference in the SPT, indicating impaired reward sensitivity and anhedonia (Fig. 3j, k). Notably, MeCP2 overexpression in NAc D2 neurons normalized both behavioral phenotypes, reducing immobility and restoring sucrose preference to control levels. Together, these results support the view that MeCP2 in NAc D2R neurons is an important modulator of stress-associated dysregulation across affective coping and reward-related behaviors, and that its restoration attenuates CRS-induced depressive-like phenotypes.

We next sought to identify neurophysiological correlates of this behavioral rescue. Using optogenetic activation together with MEA recordings, we found that CRS reduced activity in NAc D2R neurons, and that this reduction was attenuated by MeCP2 overexpression. This is broadly consistent with prior work showing that MeCP2 deficiency can reduce excitatory synaptic function and plasticity, whereas MeCP2 overexpression can enhance these processes in other brain regions^23,38,39^. For example, MeCP2 deficiency has been associated with reduced miniature excitatory postsynaptic current (mEPSC) frequency and impaired plasticity in hippocampal neurons.

At the same time, MeCP2 effects on neuronal function are strongly context dependent and can diverge across brain regions and cell types^40–45^. Consistent with this complexity, our prior work showed that pan-neuronal MeCP2 knockdown in the dorsal striatum increases overall activity levels^46^. Together, these studies underscore the importance of defining MeCP2 function within specific circuits and cellular populations.

To elucidate the molecular basis of MeCP2’s effects, we conducted spatial transcriptomic profiling of D2R neurons from naive controls (NC), CRS-exposed controls (CC) and MeCP2-overexpressing mice (CM). MeCP2 overexpression was associated with partial attenuation of numerous genes dysregulated by CRS, with prominent representation of synaptic function-related programs, including annotations linked to glutamatergic synaptic signaling. Given that glutamatergic transmission is a well-established target for antidepressant interventions^47,48^, these results are consistent with the possibility that MeCP2 engages stress-sensitive synaptic programs relevant to depressive-like phenotypes. Future studies will be required to determine how MeCP2-dependent transcriptional states relate to functional changes in glutamatergic transmission and synaptic plasticity in this cell population.

We also observed MeCP2-associated shifts in ion channel-related transcripts, including potassium channel-associated genes, in the CM versus CC comparison. Potassium channels have been implicated as potential therapeutic targets for depression^49,50^, and in our dataset MeCP2 manipulation was associated with both increased and decreased expression of specific channel-related transcripts, suggesting a nuanced relationship with ion channel homeostasis. Because we did not directly measure potassium currents or perform gene-specific perturbations, these channel-related signatures should be interpreted as transcript-level correlates accompanying activity-state normalization rather than evidence of a causal mechanism.

In addition, MeCP2 overexpression was associated with attenuation of CRS-linked transcript sets annotated to palmitoylation^51,52^, cilium structure and function^53,54^, and actin filament binding^52,55^ as inferred from GO/annotation analyses. These enrichment results should be interpreted as hypothesis-generating functional summaries rather than mechanistic evidence. Notably, MeCP2 overexpression in NAc D2R neurons was sufficient to ameliorate depressive-like behaviors and was accompanied by changes in the expression of multiple synaptic function-related genes. Supporting this, transcriptomic analysis revealed that the expression of *Fos*—a well-established marker of neuronal activity—was downregulated in the CC group but shifted toward control levels in the CM group. These molecular patterns parallel our electrophysiological recordings and are consistent with an association between MeCP2 restoration and a shift toward baseline activity states within this cell population. Overall, these findings provide a hypothesis-generating framework to interpret how MeCP2-dependent transcriptional programs may interface with stress-associated cellular processes in NAc D2R neurons. At the same time, stress-related signaling within the NAc is shaped not only by cell-intrinsic transcriptional states but also by the broader local milieu and afferent inputs.

In this context, our protein-level measurements indicate that CRS elevates bulk NAc BDNF and that this elevation is attenuated by NAc D2R-restricted MeCP2 overexpression (Supplementary Fig. 20a). Notably, *Bdnf* transcripts were not detected in our GeoMx DSP ROIs from virally labeled D2R neurons, aligning with prior work reporting low somatic Bdnf mRNA in striatal neurons and emphasizing that BDNF abundance in striatal targets can be strongly influenced by afferent inputs and anterograde transport^56–59^. Consistent with this view, BDNF immunoreactivity quantified within eGFP-labeled D2R neuronal somata did not show clear group differences (Supplementary Fig. 20b, c). Together, these data suggest that stress- and MeCP2-associated differences in bulk NAc BDNF probably reflect changes in the broader local BDNF milieu, although the present study does not resolve the cellular sources or subcellular compartments underlying the ELISA signal. As a complementary, exploratory signaling readout, we assessed CREB and MeCP2 phosphorylation in our model (Supplementary Figs. 21 and 22). While we observed a phosphorylation-state shift in the MeCP2 restoration condition under CRS (CM), these measures were not designed to establish mechanistic coupling between CREB and MeCP2 or a defined CREB → BDNF transcriptional pathway in the NAc. Accordingly, we present these data as supplementary correlates accompanying behavioral and physiological attenuation, and note that targeted perturbation and pathway-/cell-type-resolved experiments will be required to determine causal relationships.

In an extended analysis, we examined whether NAc D2R-restricted MeCP2 overexpression was accompanied by CRS-responsive molecular signatures in the VP, a major downstream target of NAc D2R neurons. Because behavioral phenotypes probably emerge from coordinated activity across interconnected regions, we asked whether MeCP2-associated molecular shifts observed in the NAc might be accompanied by parallel signatures in anatomically connected regions, including the VP, which has been implicated in mood regulation and motivational processing^34,60^. Notably, VP gene sets showing CRS responsiveness also exhibited MeCP2-associated shifts, and GO/annotation analyses highlighted several enriched categories in these VP signatures, including myelination-related terms. However, because VP ROIs were not projection-resolved and reflect bulk tissue composition, these findings should be interpreted as associative downstream signatures rather than evidence for a direct NAc → VP projection-specific mechanism. Accordingly, future studies using pathway-resolved circuit approaches—such as projection-targeted chemogenetics or optogenetics (for example, DREADDs)—will be required to test whether specific NAc input/output routes contribute to the behavioral effects observed in this model. In addition, given prior reports linking chronic stress to alterations in oligodendrocyte function and myelin integrity in depression-relevant circuits^61^, future work should directly assess oligodendrocyte/myelin measures in the VP and NAc-centered circuits to determine whether myelin-related processes contribute to stress-associated phenotypes and their attenuation following MeCP2 restoration.

The NAc serves as an integrative hub within depression-relevant circuitry, receiving convergent stress-, emotion- and reward-related signals from multiple limbic regions. Prior work indicates that afferent pathways to the NAc—including inputs from the ventral hippocampus and medial prefrontal cortex^62,63^—can modulate depression-related behaviors in a context- and pathway-dependent manner, underscoring the complexity of NAc circuit function. Within this framework, our findings identify NAc D2R neurons as a key cellular substrate in which chronic stress-associated molecular, physiological and behavioral alterations converge, adding a molecular dimension to existing circuit models of depression. In the present study, MeCP2 manipulation was restricted to this population; accordingly, our results are best interpreted as modulation of NAc D2R neuronal state, which may influence how these neurons integrate and respond to convergent afferent activity under chronic stress.

Interestingly, while CRS reduced MeCP2 protein levels in NAc D2R neurons, *MeCP2* mRNA levels remained unchanged. This dissociation suggests that CRS impacts MeCP2 abundance through mechanisms beyond changes in transcript levels, potentially involving altered translation and/or post-translational regulation^64,65^; however, the present data do not distinguish among altered translation, protein stability or degradation mechanisms.

Several boundaries of the present study should be highlighted. First, all experiments were performed in male mice, and future work will be needed to determine whether MeCP2-dependent phenotypes generalize across sex. Second, while MeCP2 manipulation was restricted to NAc D2R neurons, the present study was not designed to resolve pathway-specific circuit mechanisms; thus, we did not directly test whether particular NAc input/output routes—including NAc–VP circuitry—are necessary or sufficient for the behavioral effects observed. Third, transcriptomic and GO/annotation signatures (including ion channel-related categories) were used to contextualize physiological normalization, but we did not perform functional validation at the level of ionic currents, synaptic transmission, or gene-specific perturbations. Finally, although CRS reduced MeCP2 protein despite unchanged *MeCP2* mRNA, our data do not identify the upstream mechanisms governing MeCP2 abundance (for example, translational control or post-translational regulation).

Building on the present findings, future studies should use pathway- and projection-resolved circuit approaches to define how NAc D2R neurons influence stress-related behaviors through downstream targets (including the VP). In parallel, complementary electrophysiological analyses will be important to determine how MeCP2-dependent transcriptional states relate to functional changes in synaptic transmission and neuronal activity, and targeted experiments will be needed to functionally validate key candidate genes emerging from our transcriptomic profiling.

This study demonstrates that cell-type-restricted MeCP2 overexpression in NAc D2R neurons attenuates CRS-induced depressive-like behaviors and reveals accompanying transcriptomic and physiological signatures within this defined neuronal population. Collectively, our findings position MeCP2 as an important molecular regulator of stress-associated adaptations in NAc D2R neurons and support a framework for linking cell-type-specific chromatin regulation to circuit-level and behavioral outcomes.

## Supplementary information

Supplementary Information

Supplementary Tables (Tables S1-S40)

## Data Availability

Processed results of the transcriptomic analysis are available in the Supplementary Information. Due to the size and nature of the raw sequencing data, they are available from the corresponding author upon reasonable request.
