# Supplementary material for: MeCP2 regulates cell-type-specific functions of depressive-like symptoms in the nucleus accumbens
Source: Exp Mol Med. 2026 May 12;58(5):1657–73. doi: 10.1038/s12276-026-01721-3 (PMC13234275; doi:10.1038/s12276-026-01721-3)
Supplement: Supplementary file 1 — Supplementary Information [file 12276_2026_1721_MOESM1_ESM.pdf]

MeCP2 regulates cell type-specific functions of depressive-like symptoms in the nucleus accumbens

Bae et al.

*Corresponding author: H.-I. Im (E- mail: him@kist.re.kr)

This file includes:

Supplementary Figures. 1-26

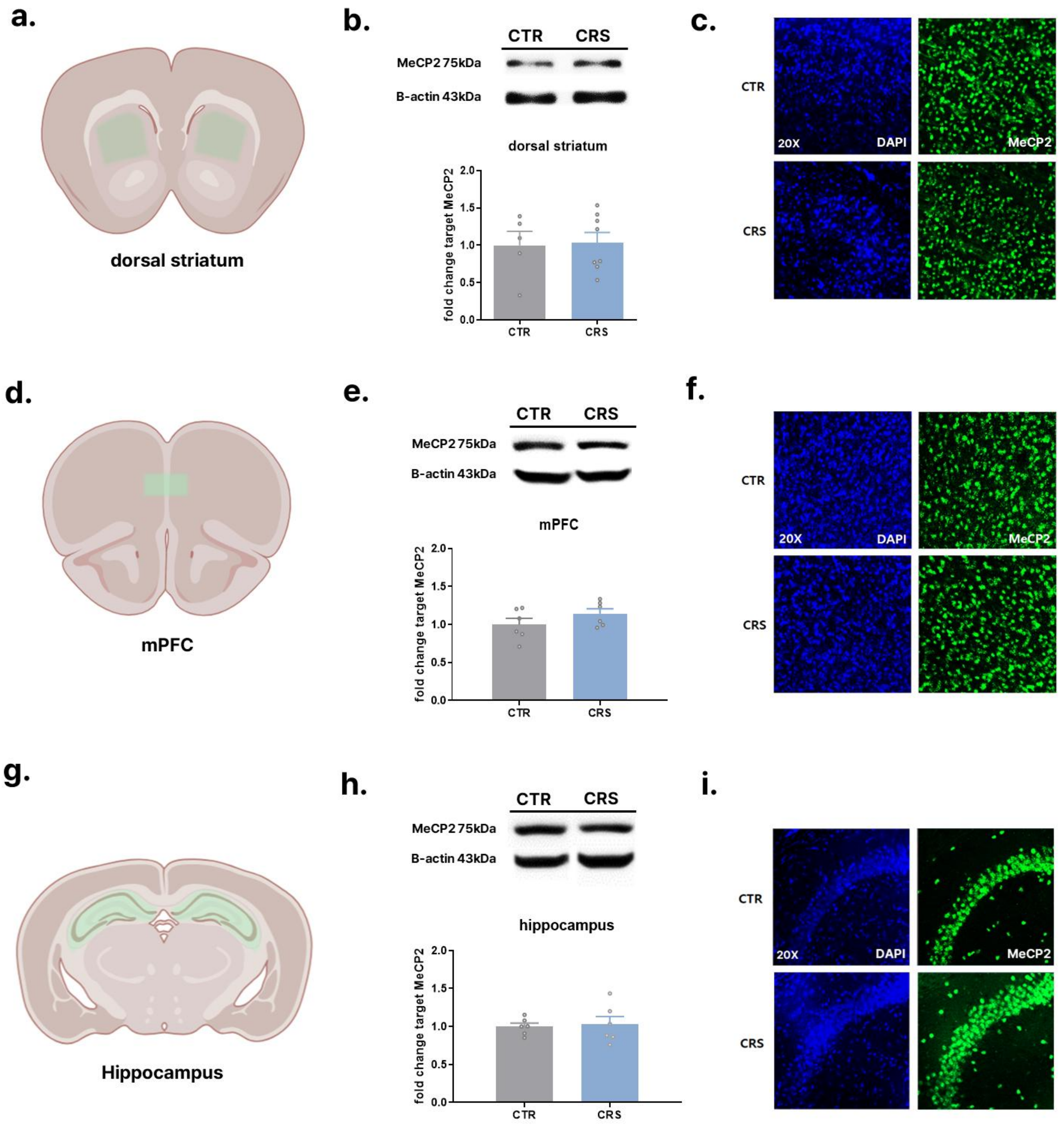

Supplementary Fig. 1 MeCP2 protein levels in non-target brain regions remain unchanged following chronic restraint stress (CRS). **a–c** Dorsal striatum: a Schematic of the sampled region. **b** Representative Western blot images (top) and quantification (bottom) showing no significant difference in MeCP2 expression between control and CRS groups (unpaired two-tailed t-test: $t = 0.1685$, $p = 0.8692$, $df = 11$; $n = 5–8$/group). **c** Representative immunofluorescence images of MeCP2 (green) and DAPI (blue) confirming comparable expression across groups (20×). **d–f** Medial prefrontal cortex (mPFC): **d** Schematic of the sampled region. **e** Western blot analysis demonstrating no CRS-associated change in MeCP2 levels (unpaired two-tailed t-test: $t = 1.343$, $p = 0.2090$, $df = 10$; $n = 6$/group). **f** Representative immunofluorescence images showing consistent MeCP2 labeling between groups (20×). **g–i** Hippocampus: **g** Schematic of the sampled region. **h** Western blot quantification showing no significant MeCP2 alteration after CRS (unpaired two-tailed t-test: $t = 0.2692$, $p =$

0.7933, df = 10; n = 6/group). **i** Representative immunofluorescence images confirming stable MeCP2 expression in the hippocampus (20×). Data in panels (b, e, h) are presented as mean ± SEM.

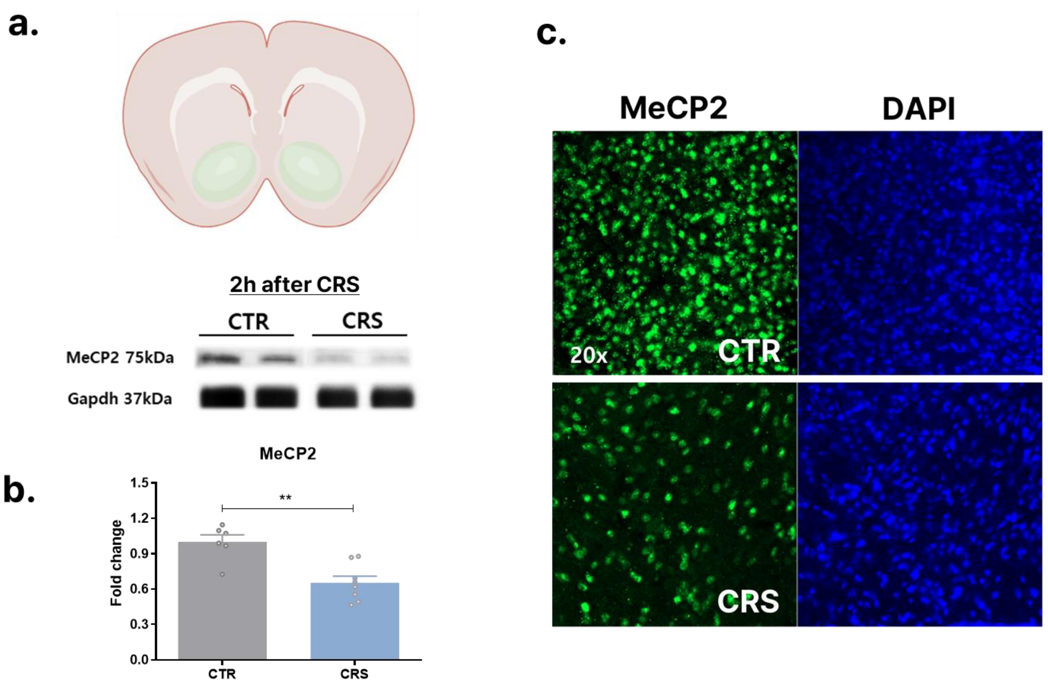

Supplementary Fig. 2 CRS induces a reduction of MeCP2 protein expression in the NAc. **a** Schematic and representative Western blot images showing MeCP2 expression in the nucleus accumbens (NAc) 2 h after the final CRS session. **b** Quantification of MeCP2 protein levels normalized to β-actin (two-tailed unpaired t-test: $t = 4.166$, $p = 0.0013$, df = 12; CTR: $n = 6$, CRS: $n = 8$). Data are presented as mean ± SEM (**p < 0.01). **c** Representative immunofluorescence images showing reduced MeCP2 signal in CRS mice (MeCP2: green; DAPI: blue; 20×).

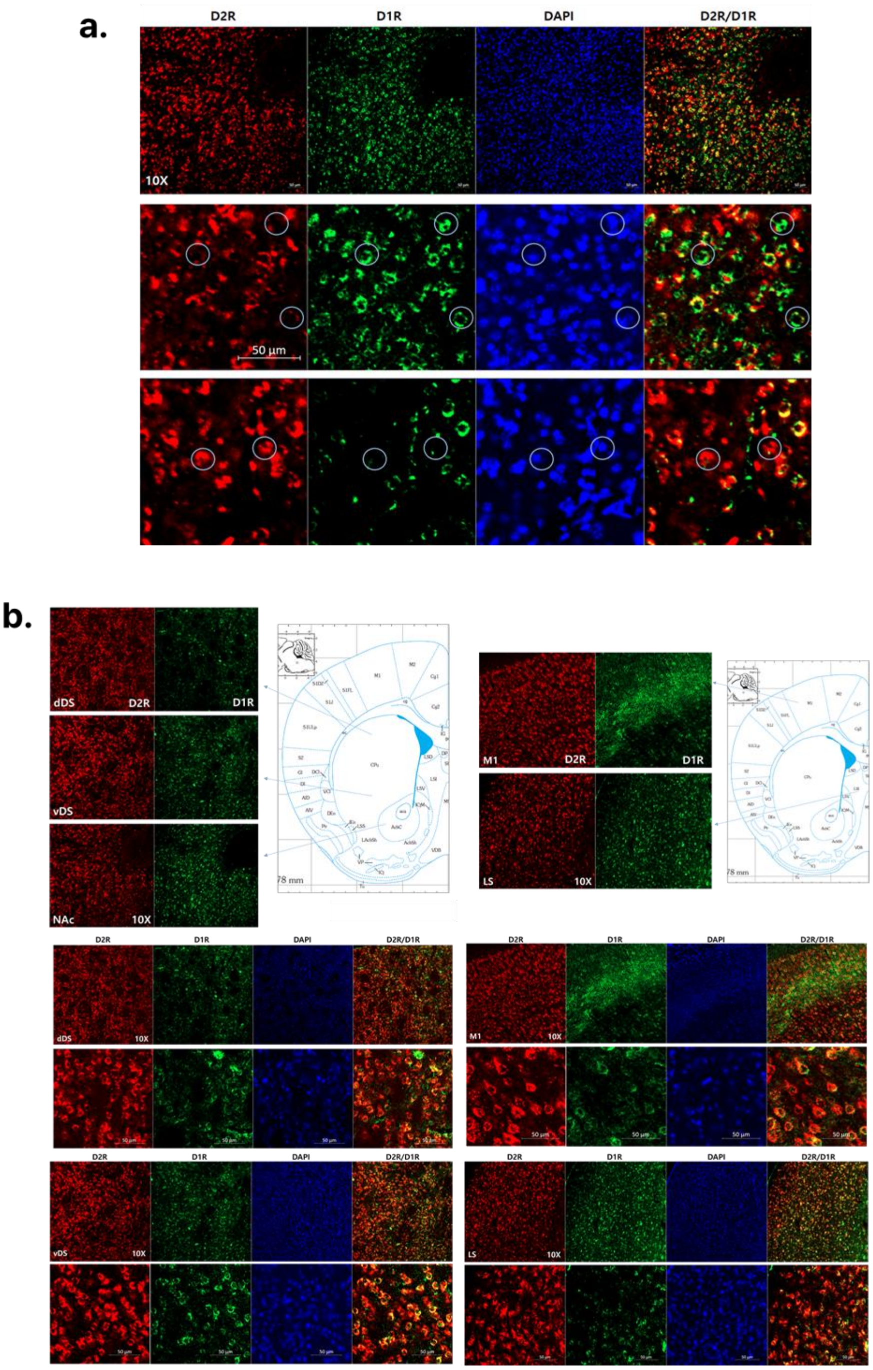

Supplementary Fig. 3 Validation of D1R and D2R antibody specificity and regional expression patterns. D2R (red), D1R (green), DAPI (blue) **a** Representative immunofluorescence images from the nucleus accumbens (NAc) showing non-overlapping labeling of D1R- and D2R-expressing neurons, with a small subset of double-positive cells (circles). **b** Regional validation across predominantly non-overlapping brain areas, including dorsal striatum (dDS, vDS), primary motor cortex (M1), and lateral septum (LS). D1R and D2R signals predominantly localize to distinct neuronal populations, with occasional overlap depending on region. Coordinates and anatomical reference boundaries correspond to *The Mouse Brain in Stereotaxic Coordinates* (Franklin & Paxinos, 2007). Images shown at 10× and 50 µm scale where indicated.

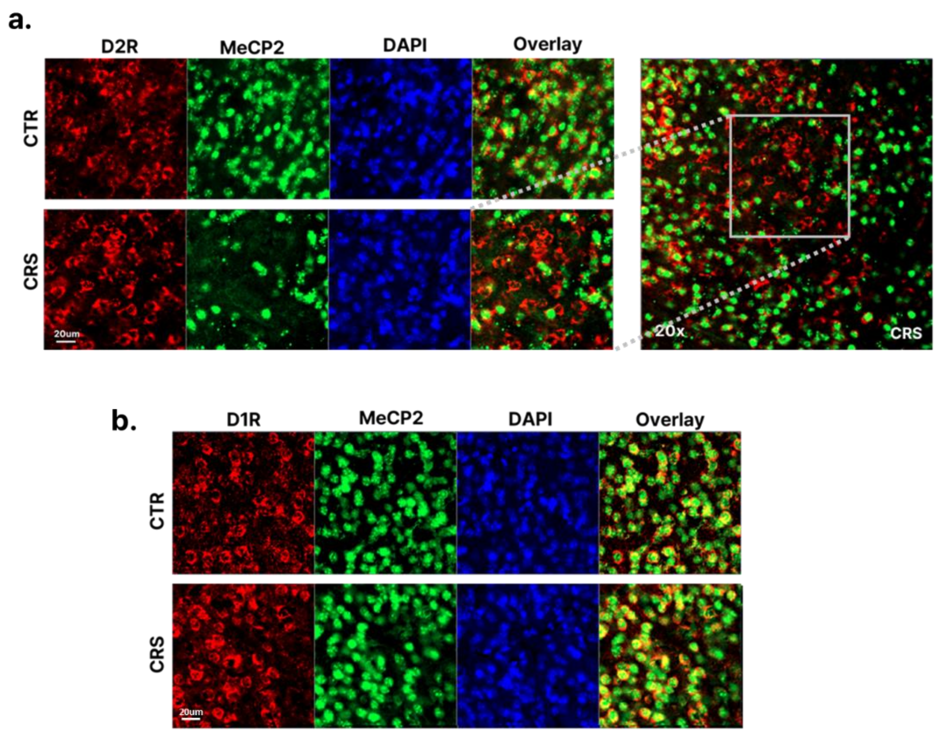

Supplementary Fig. 4 Cell type–specific MeCP2 expression patterns in the NAc following CRS. D2R (red), MeCP2 (green), DAPI (blue). Representative immunofluorescence images showing MeCP2 localization in D2R$^+$ and D1R$^+$ neuronal populations in the nucleus accumbens (NAc) three days after the final CRS exposure. **a** MeCP2 signal appears reduced in D2R-expressing neurons following CRS. **b** MeCP2 expression in D1R-expressing neurons remains comparable to the control condition. Images acquired at 20×. Scale bar: 20 µm.

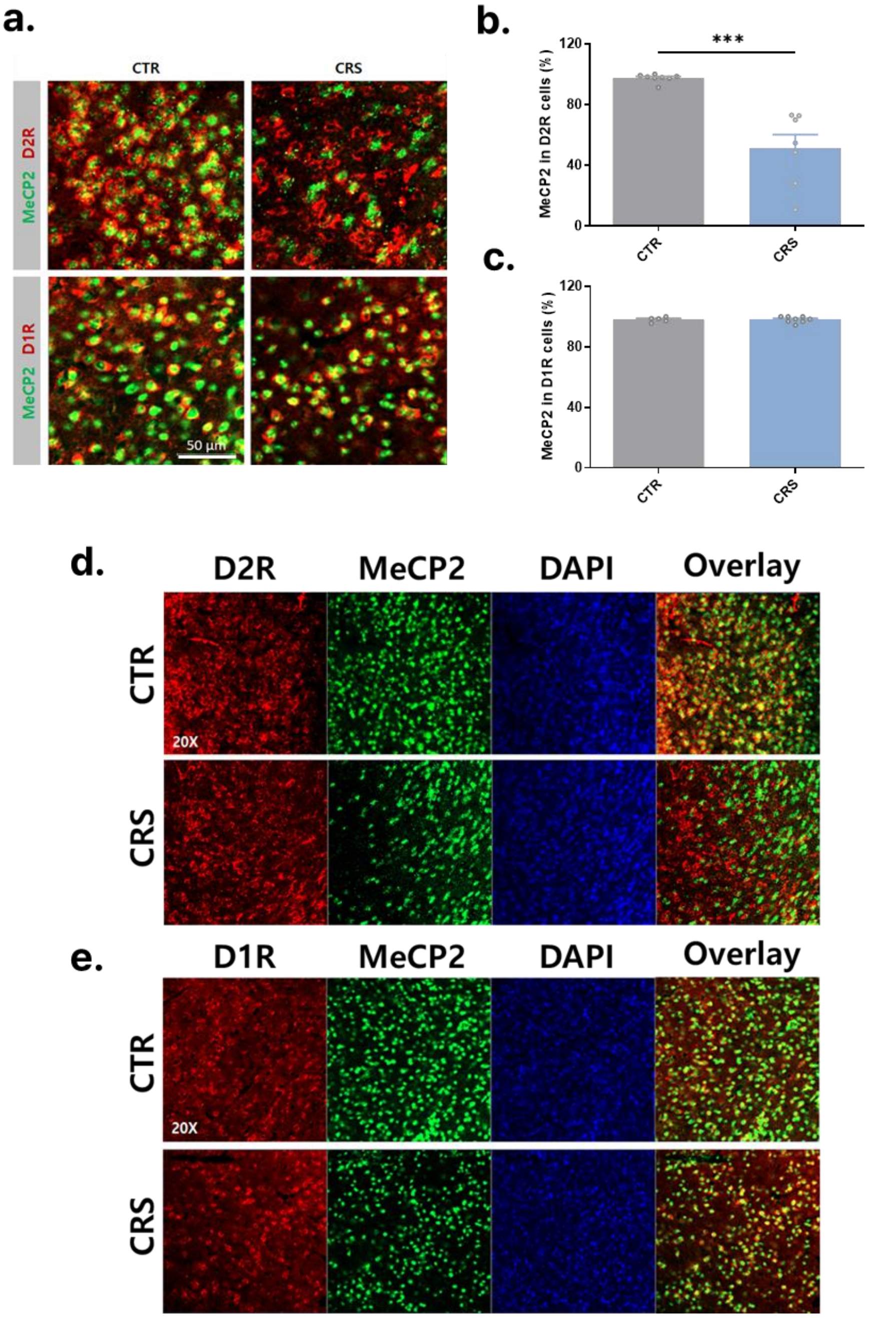

Supplementary Fig. 5 Cell-type–specific reduction of MeCP2 expression in the NAc following CRS (2 h after final stress exposure) **a** Representative immunofluorescence images showing CRS-induced reduction of MeCP2 expression in D2R-expressing neurons, whereas D1R-expressing neurons showed no measurable decrease. Images acquired at 20×; scale bar = 50 µm. **b** Quantification of the percentage of D2R^{+} neurons that were MeCP2^{+} shows a significant reduction in CRS mice (unpaired two-tailed t-test: $t = 5.447$, $p = 0.0001$, $df = 13$; $n = 8$ vs. 7 mice/group). **c** Quantification of MeCP2-positive D1R neurons showing no significant difference between groups ($t = 0.045$, $p = 0.9651$, $df = 11$; $n = 5$ vs. 8 mice/group). **d, e** Additional representative images validating cell-type–specific MeCP2 changes. Data shown as mean ± SEM; ***$p < 0.001$.

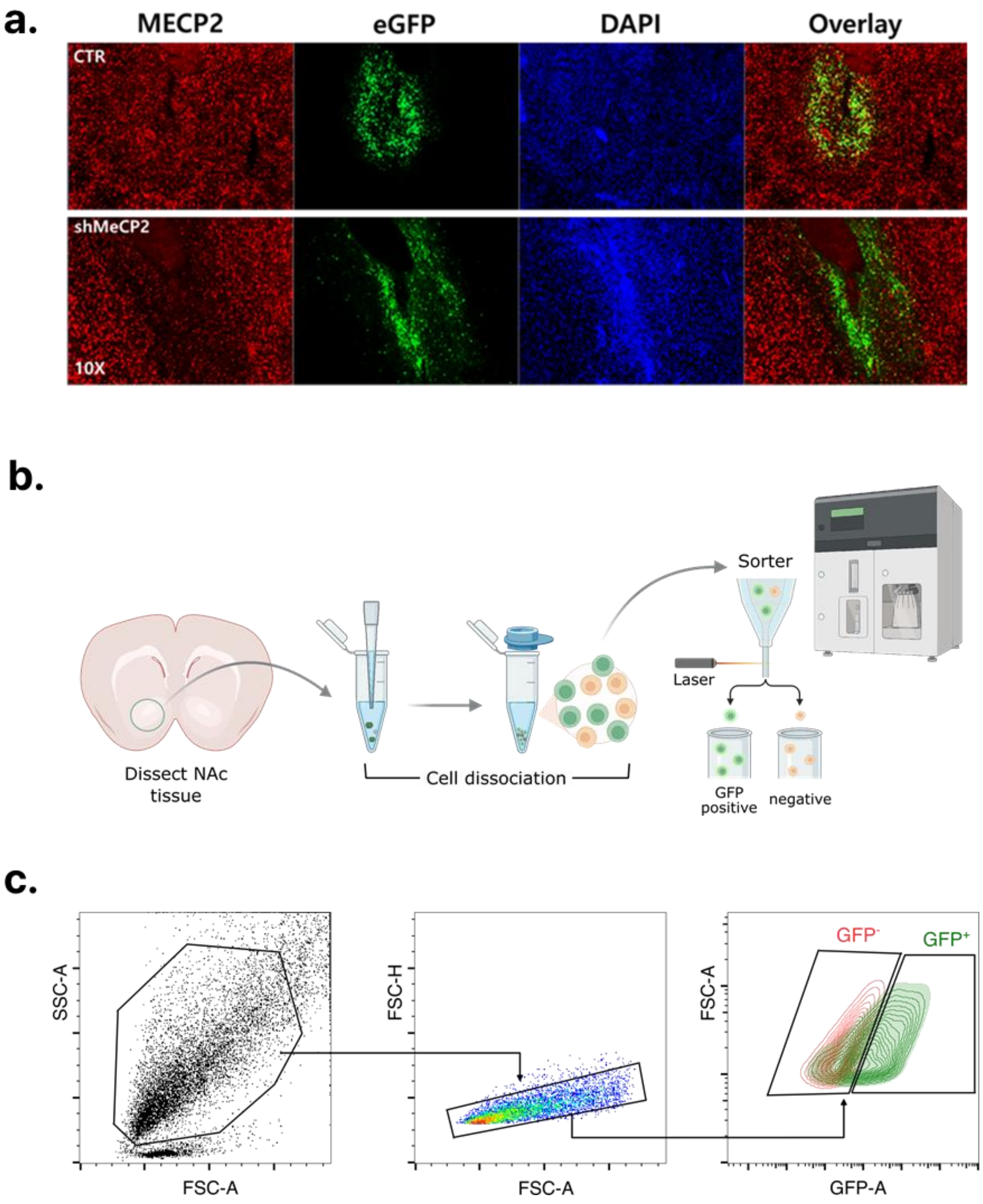

Supplementary Fig. 6 Functional validation of AAV-G-CREon-shMeCP2 and cell-sorting workflow **a** Representative immunofluorescence images showing MeCP2 knockdown in the NAc. MeCP2 (red), eGFP (green), and DAPI (blue) are shown with merged images. Reduced MeCP2 signal was observed preferentially in eGFP-positive cells in the shMeCP2 condition, consistent with successful Cre-dependent viral targeting (10× magnification). **b** Schematic illustration of the workflow for brain tissue dissociation and preparation for fluorescence-activated cell sorting (FACS). **c** Representative FACS plots illustrating the gating strategy used to isolate GFP-positive (GFP^+) and GFP-negative (GFP^-) cell populations based on GFP fluorescence. These sorted populations were used for downstream gene expression analyses shown in Fig. 2f,g.

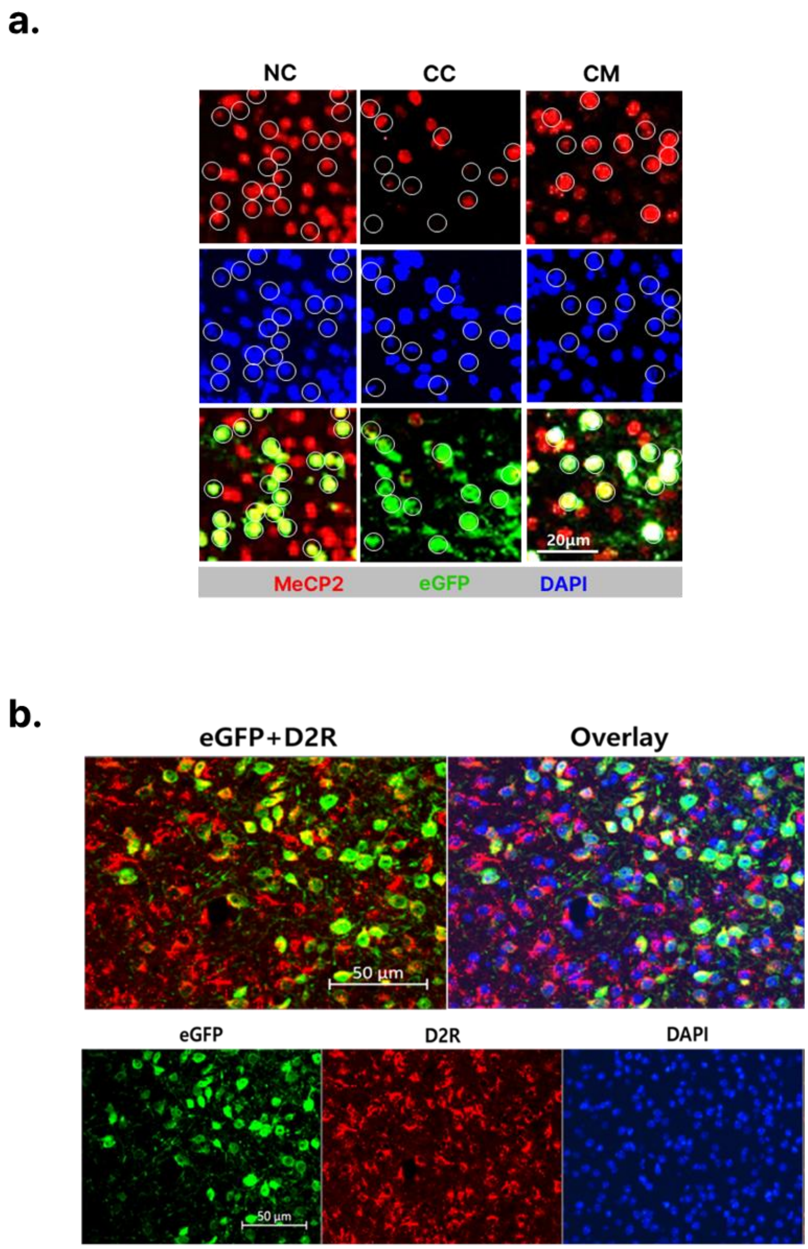

Supplementary Fig. 7 Representative validation of Cre-dependent MeCP2 overexpression and D2R neuron–restricted targeting in the NAc. **a** Representative immunofluorescence images from the three experimental groups (NC, CC, CM) showing MeCP2 (red), eGFP (green), and DAPI (blue) in the NAc. Images were acquired at 20×; scale bar: 50 µm. **b** Representative immunofluorescence images confirming cell-type–restricted viral targeting, showing co-expression of eGFP (green) and D2 receptor (red) in the NAc. Scale bar: 50 µm.

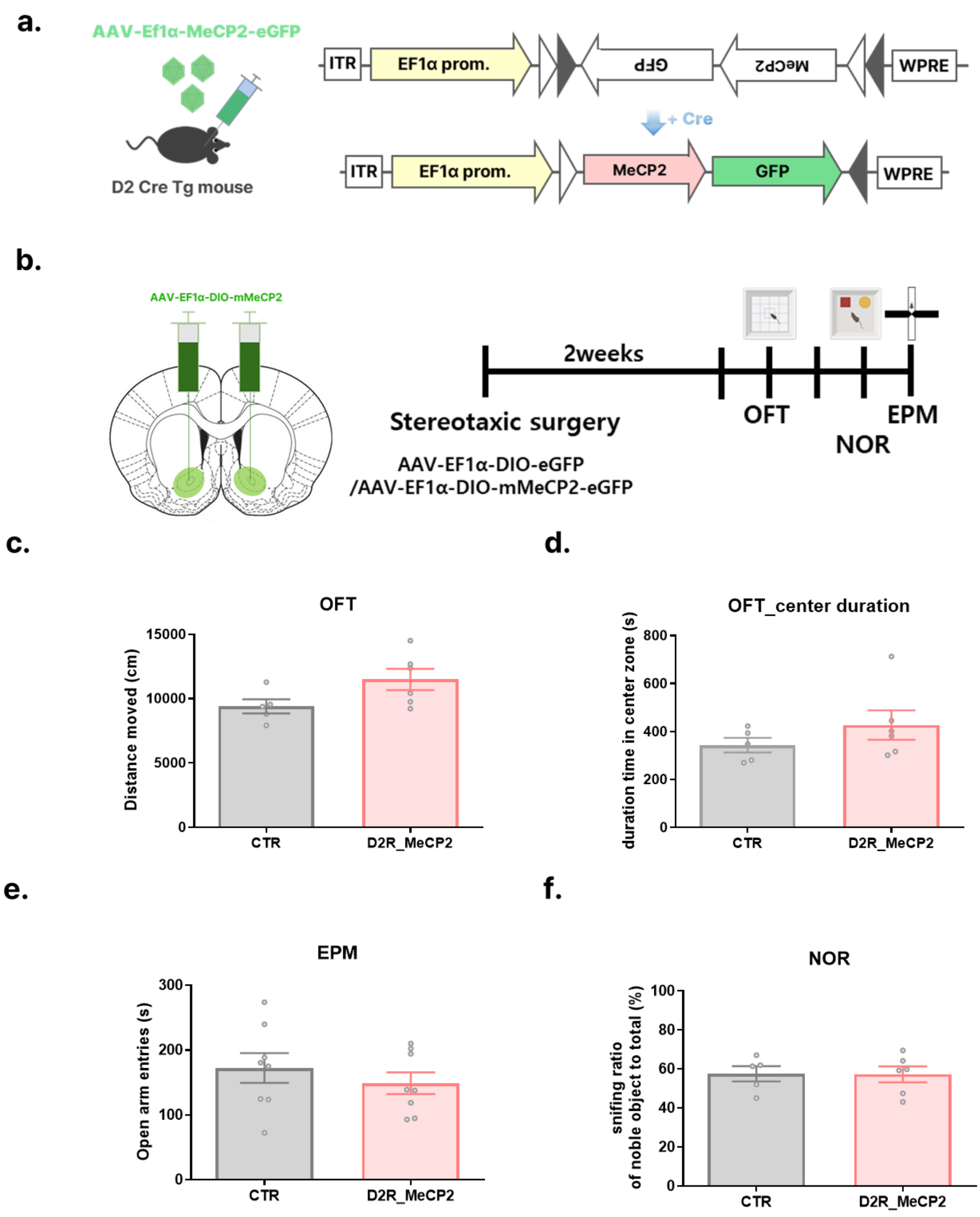

Supplementary Fig. 8 Effect of genetic MeCP2 upregulation in NAc D2R neurons under non-stress conditions **a** Schematic diagram of the AAV construct enabling Cre-dependent MeCP2 overexpression. **b** Experimental timeline for behavioral assessment following targeted MeCP2 overexpression in D2R neurons of the NAc. **c–f** Behavioral performance in normal mice injected with AAV-MeCP2 showed no significant alterations in locomotion, anxiety-like behavior, or cognitive function. **c** Total distance traveled in the open field test (two-tailed t-test, t = 2.013, p = 0.0749, df = 9, n = 5–6/group). **d** Time spent in the center zone of the open field test (t = 1.147, p = 0.2808). **e** Time spent in open arms in the elevated plus maze (t = 0.8254, p = 0.4230, df = 14, n = 8/group). **f** Exploration ratio for the novel object during the novel object recognition test (t = 0.047, p = 0.9633, df = 9, n = 5–6/group). Data are presented as mean ± SEM.

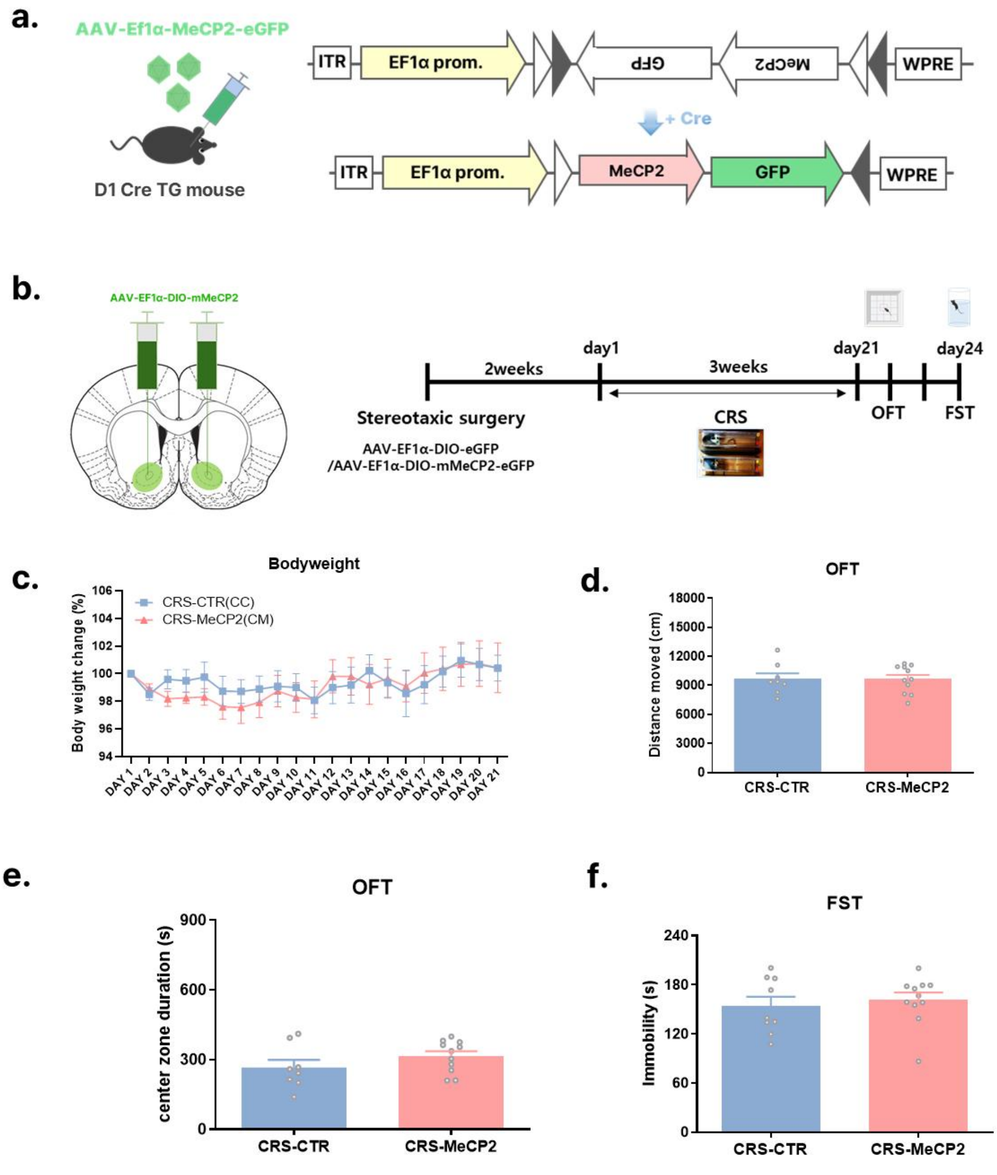

Supplementary Fig. 9 Effect of MeCP2 overexpression in D1R neurons under chronic restraint stress **a** Schematic of the AAV construct used for Cre-dependent MeCP2 overexpression selectively in D1R neurons (D1-Cre mice). **b** Experimental timeline and behavioral testing schedule to evaluate the impact of MeCP2 upregulation in D1R neurons during CRS. **c** Body weight changes across CRS exposure showed no group differences (two-way mixed ANOVA, CC vs. CM: $F(1,17) = 0.041$, $p = 0.8410$). **d–f** Behavioral assessment demonstrated no significant effects of MeCP2 overexpression in D1R neurons on locomotor activity, anxiety-like behavior–related measure, or behavioral despair. **d** Distance traveled in the open field test (two-tailed t-test, $t = 0.026$, $p = 0.9792$, df = 17, n = 8, 11/group). **e** Time spent in the center zone of the open field test ($t = 1.338$, $p = 0.1984$, df = 17). **f** Immobility time in the forced swim test ($t = 0.521$, $p = 0.6084$, df = 18, n = 9–11/group). Data are shown as mean ± SEM.

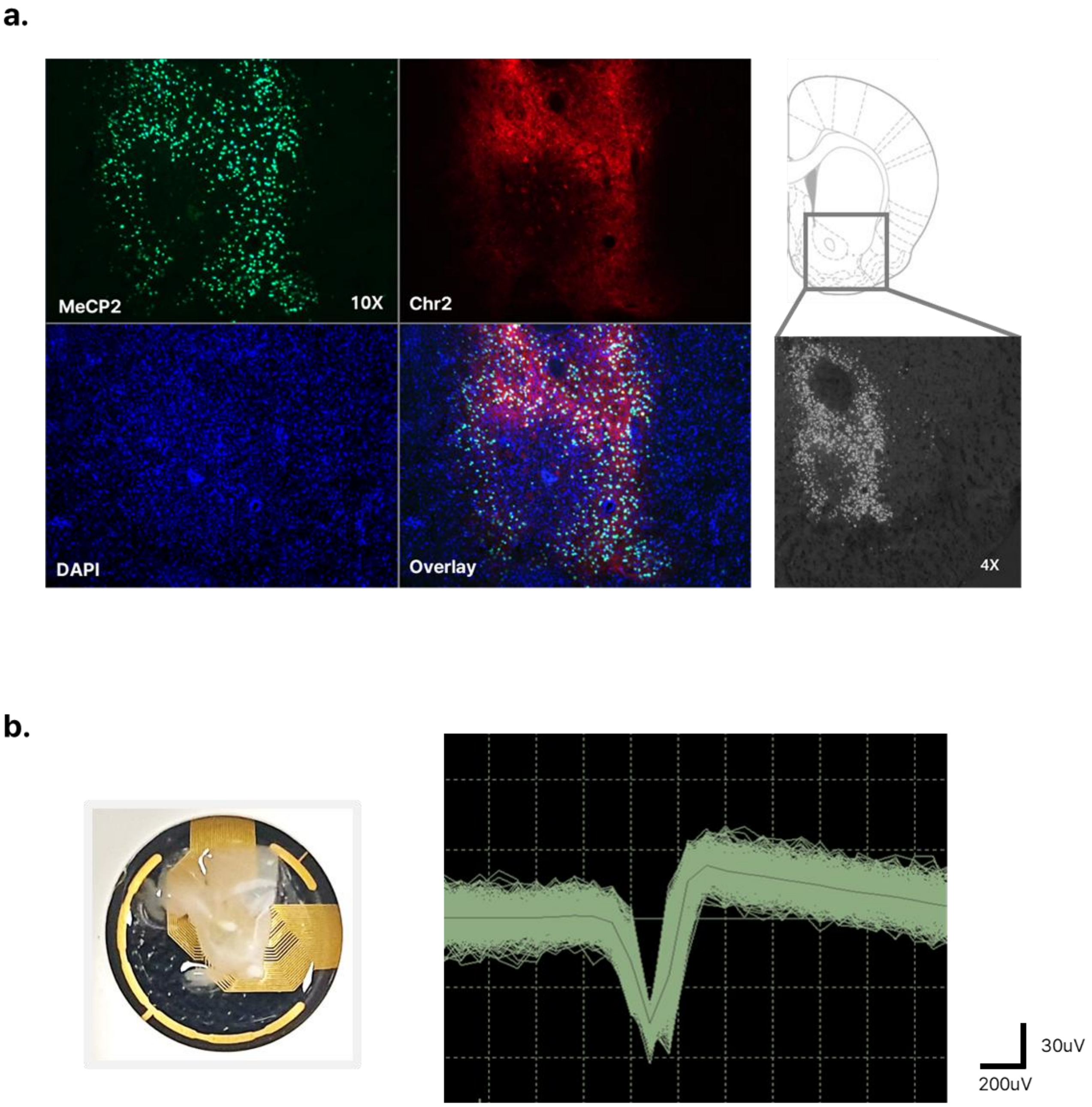

Supplementary Fig. 10 Neural activity recording in the NAc using optogenetics and MEA **a** Representative immunofluorescence images showing coexpression of MeCP2 (green) and ChR2 (red) in the nucleus accumbens. DAPI (blue) marks nuclei (10×). Right: schematic brain atlas reference indicating viral targeting and corresponding monochrome fluorescence image showing the localized viral expression area (4×). **b** Sagittal brain slices containing the NAc were positioned on a 64-channel multielectrode array (MEA) for ex vivo recordings. Left: placement of the brain slice on the MEA platform. Right: representative averaged spike waveform recorded following optogenetic stimulation of the NAc (~280 μm-thick slice).

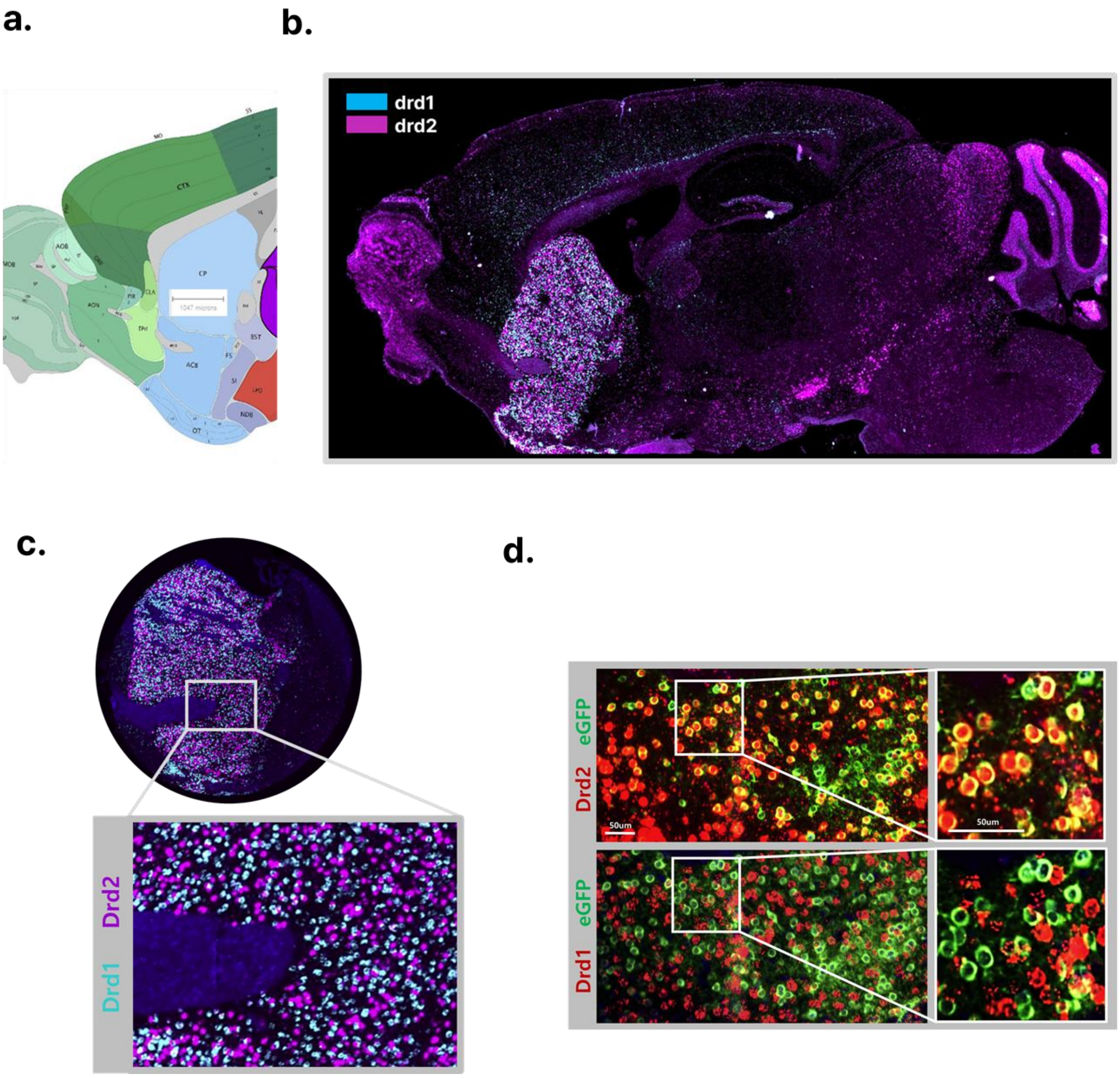

Supplementary Fig. 11 Cell-type identification using FISH for GeoMX DSP region selection **a** Atlas reference of a sagittal mouse brain section including the striatum (adapted from Allen Brain Atlas). **b** Fluorescence in situ hybridization (FISH) images showing spatial expression of *Drd1* (cyan) / *Drd2* (magenta) mRNA across the striatum, confirming high and distinct receptor expression patterns. **c** High-magnification FISH image from the nucleus accumbens demonstrating predominantly segregated of Drd1- and Drd2-expressing neuronal populations. **d** Representative FISH/imaging panels showing co-localization of *eGFP* with *Drd2* and limited overlap with *Drd1*, supporting cell-type–specific targeting of D2R neurons by AAV-GFP.

a.

Target name	Fold changes	-$\log_{10}$ pvalue
Adora2a	21.16	9.280
Cre	16.19	9.741
Penk	15.00	12.216
Gpr6	11.38	11.980
Drd2	10.10	9.353
Grik3	9.58	7.147
P2ry1	6.84	10.177
Gpr52	6.60	6.988
Unc5d	4.53	5.821
Sp9	4.18	4.151
Gnas	3.27	5.101
Ecel1	3.17	1.804
Oprd1	3.15	5.657
Ptprm	3.09	5.071
Cacna2d2	2.99	6.491
Sox2	2.98	5.217
Plxdc1	2.78	6.481
Htr2c	2.59	6.073
Adk	2.58	4.792
Nrgn	2.58	2.384
Crhr1	2.52	4.615
Nt5e	2.49	6.071
Tpbgl	2.40	2.626
Fig4	2.40	4.029
Cbln4	2.34	3.220
Ndnf	2.32	2.826
Lhx8	2.29	2.961
Zfp608	2.29	4.231
Gucy1a1	2.25	3.786
Sema6c	2.21	4.178
Myo5b	2.16	2.942
Chrna4	2.16	2.962
B3gat1	2.11	5.071
Ndst3	2.09	3.696
Cilp	2.02	1.420
Wnt7a	2.02	2.550
Ebf4	2.01	2.468

b.

Target name	Fold changes	-$\log_{10}$ pvalue
Drd1	-9.29	7.047
Pdyn	-8.82	7.503
Tac1	-7.55	4.968
Slc35d3	-7.06	5.742
Chrm4	-6.45	4.724
Foxp2	-4.70	2.911
Sstr4	-3.30	3.804
Dlk1	-3.00	2.585
Gfra1	-2.95	1.824
Lingo2	-2.85	4.458
Cntnap3	-2.55	5.353
Ube2ql1	-2.41	5.167
Asic4	-2.30	3.888
Gnb4	-2.27	5.169
Fxyd7	-2.27	3.436
Arx	-2.25	3.165
Nrxn1	-2.23	5.561
Ltk	-2.04	2.986
Prkcg	-2.01	3.882
Ryr1	-2.00	3.352

c.

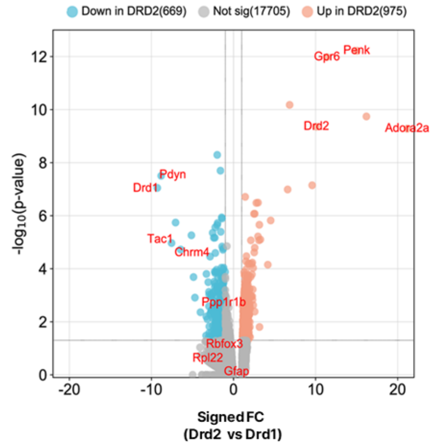

Supplementary Fig. 12 Differential expression profiling between D1R and D2R neurons. **a** Transcripts showing higher expression in D2R neurons compared with D1R neurons [threshold: |FC| > 2 (for reference, $|\log_2 FC| > 1$), nominal $p < 0.05$; |FC| as defined in the main text]. Fold change magnitude (|FC|; D2R vs D1R) and $-\log_{10}$(nominal p-value) are listed for each transcript. **b** Transcripts showing higher expression in D1R neurons compared with D2R neurons [threshold: |FC| > 2, nominal $p < 0.05$; |FC| as defined in the main text]. Here, |FC| denotes fold-change magnitude [|FC| = max(FC, 1/FC)] and direction is indicated by the D2R-enriched (a) versus D1R-enriched (b) lists. **c** Volcano plot illustrating genome-wide transcriptional differences between Drd1- and Drd2-expressing neuronal populations. The x-axis represents signed FC (D2R vs D1R), where the sign indicates direction and the magnitude corresponds to |FC| (vertical cutoffs at |FC| = 2), and the y-axis represents $-\log_{10}$(nominal p-value). p-values are nominal unless otherwise indicated. Transcripts enriched in D2R neurons are shown in red, transcripts enriched in D1R neurons in blue, and non-significant transcripts in gray.

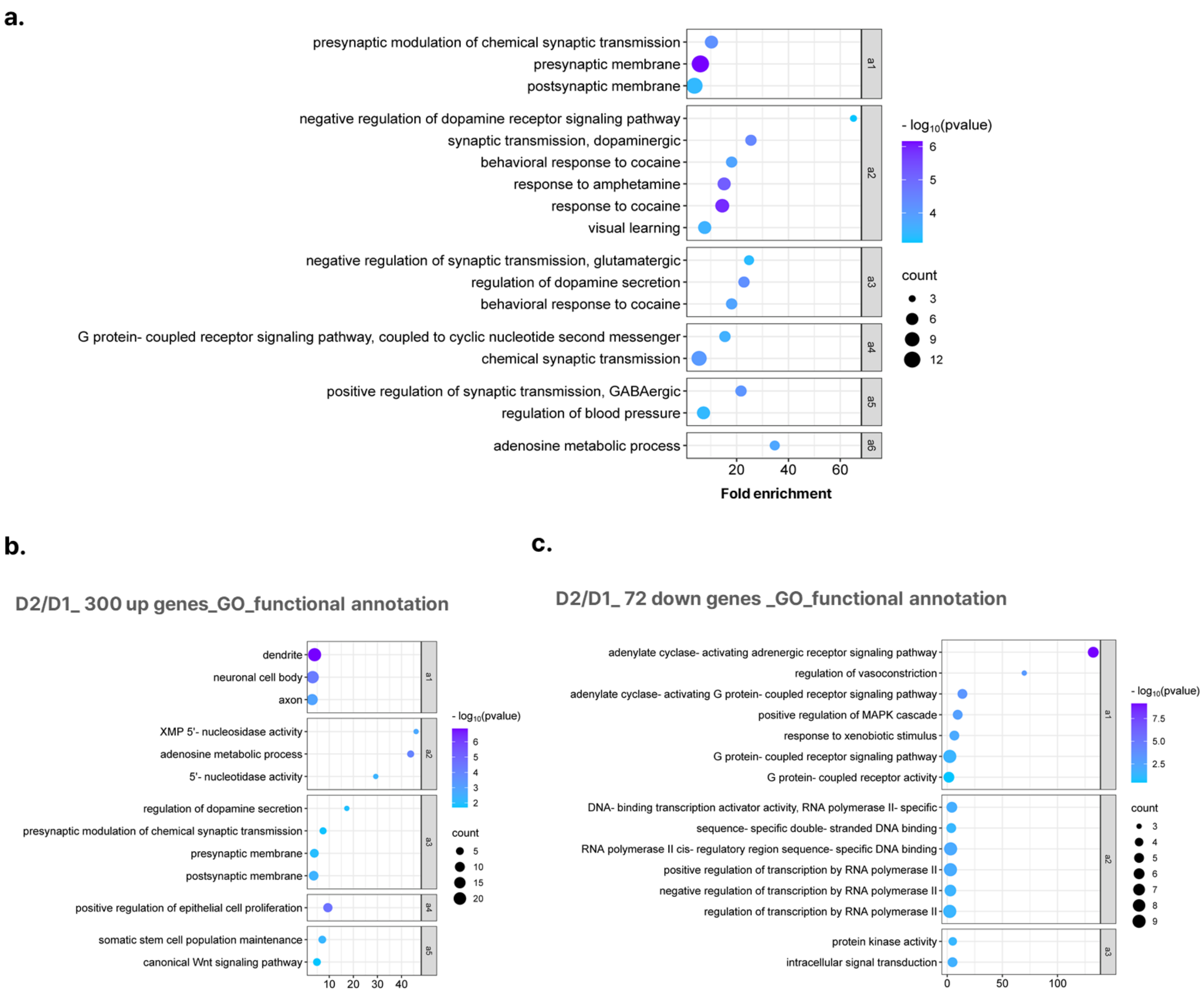

Supplementary Fig. 13 Functional annotation analysis of transcriptional differences between NAc D1R and D2R neurons. **a** Gene Ontology enrichment results based on DAVID annotation clustering applied to 372 differentially expressed genes between D2R and D1R neurons [threshold: |FC| > 1.5 (|$\log_2$FC| > 0.58), nominal $p < 0.05$; |FC| as defined in the main text]. Panels a1–a6 represent annotation clusters with enrichment scores > 1. **b** Functional annotation analysis of 300 genes upregulated in D2R neurons relative to D1R neurons (excluding Cre-dependent reporter genes). The top annotation clusters (b1–b5) meeting the enrichment criteria (enrichment score > 1, $p < 0.05$) are shown. **c** Functional annotation analysis of 72 genes downregulated in D2R neurons. Panels c1–c5 represent the top enriched functional clusters (enrichment score > 1, $p < 0.05$).

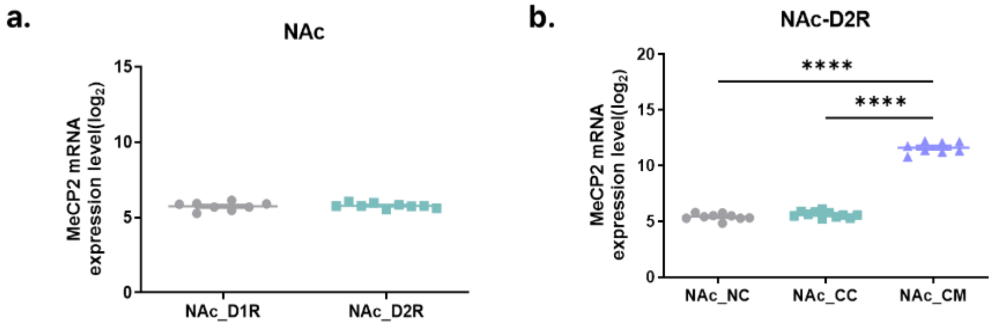

Supplementary Fig. 14 Comparison of *MeCP2* mRNA expression across cell types and experimental groups **a** Comparison of endogenous *MeCP2* mRNA levels between D1R- and D2R-expressing neurons in the NAc. No significant difference was detected (two-tailed t-test, $t = 0.3265$, $p = 0.7486$, $df = 15$; $n = 8$ D1R and 9 D2R samples). **b** *MeCP2* mRNA expression levels in NAc D2R neurons across experimental groups: NC (naive-CTR), CC (CRS-CTR), and CM (CRS-MeCP2). One-way ANOVA revealed a significant effect of group ($F(2,25) = 820.5$, $p < 0.0001$), followed by Holm-Šídák post-hoc tests showing significantly elevated *MeCP2* expression in CM compared with NC and CC (NC vs CC: $p = 0.2149$; NC vs CM: $p < 0.0001$; CC vs CM: $p < 0.0001$). ****$p < 0.0001$. Data are presented as mean ± SEM.

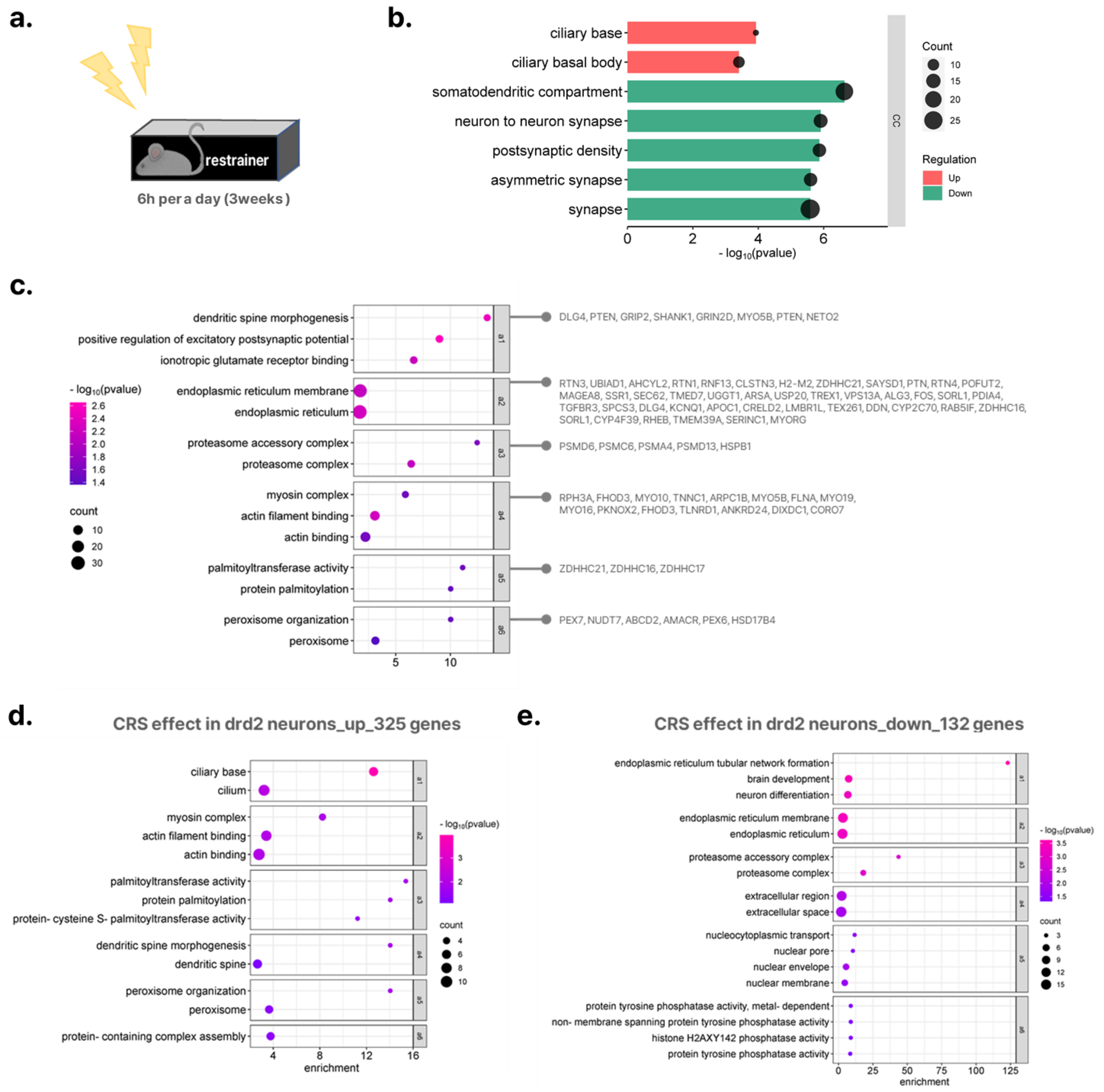

Supplementary Fig. 15 Transcriptomic effects of chronic restraint stress (CRS) in NAc D2R neurons and associated functional enrichment analysis. **a, b** Differentially expressed genes (DEGs) in NAc D2R neurons following CRS exposure. A total of 457 transcripts in NAc D2R neurons were identified as CRS-responsive (CC vs NC) using the predefined threshold of |FC| > 1.5 with nominal $p < 0.05$. **a** Schematic overview of CRS paradigm (6 h/day, 3 weeks). **b** GO cellular component enrichment of CRS-responsive genes. GO term significance is presented as $-\log_{10}$(p-value) as indicated in the plot. Red and green bars denote transcripts with increased or decreased expression, respectively. **c–e** Functional annotation clustering of CRS-responsive genes using DAVID (GO and UniProt databases). Analysis was performed across major annotation domains (molecular function, biological process, and cellular component). Clusters shown represent annotation groups

with enrichment scores > 1 and nominal $p < 0.05$ (DAVID), indicating biologically meaningful functional convergence. **c** Full grouped annotation overview. **d** Functional clusters enriched among transcripts increased by CRS. **e** Functional clusters enriched among transcripts decreased by CRS. The x-axis represents fold enrichment for each annotation category. Dot size indicates gene count per term, and dot color represents statistical significance ($-\log_{10}$(p-value)).

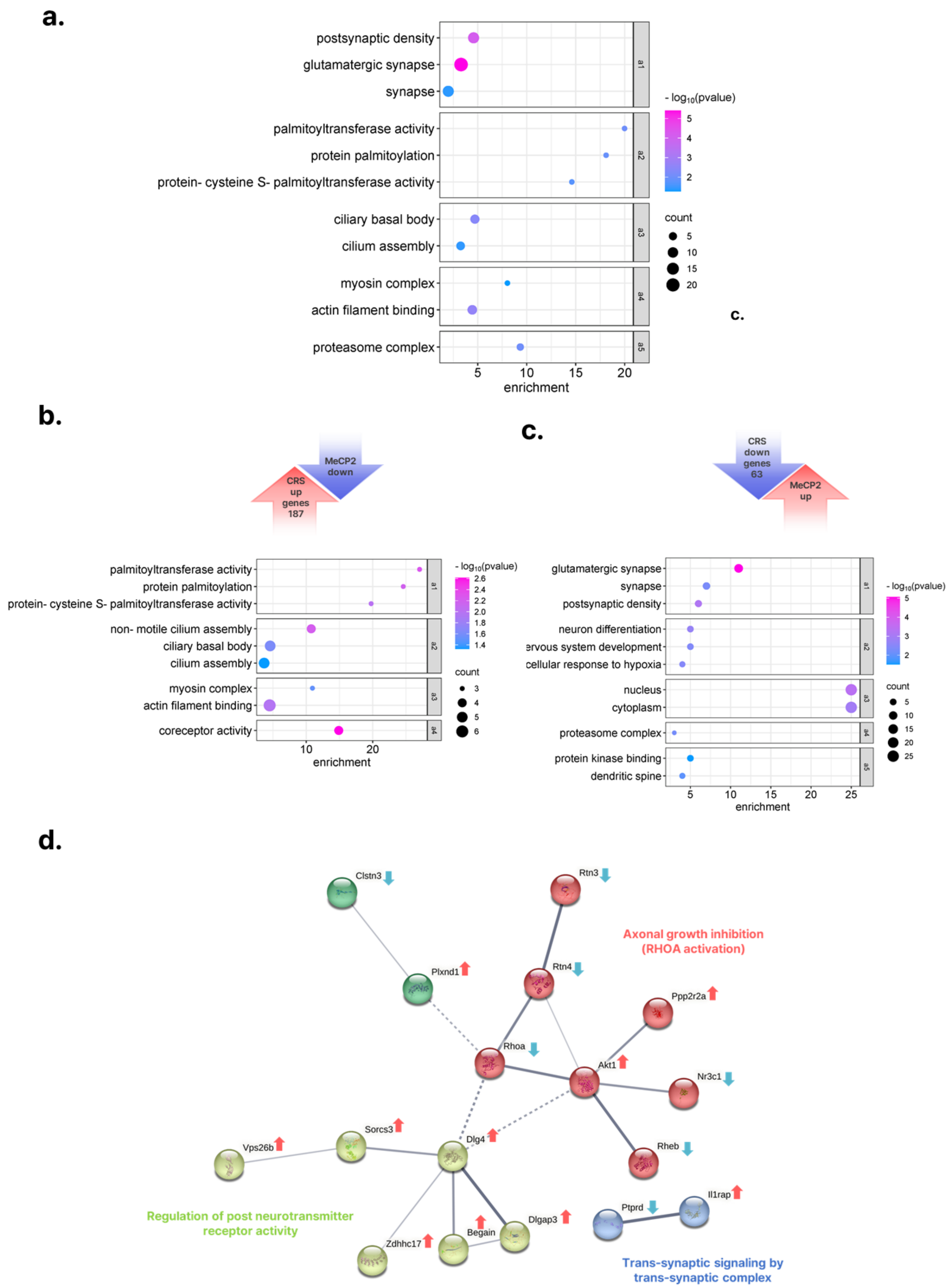
a.
postsynaptic density
glutamatergic synapse
synapse
palmitoyltransferase activity
protein palmitoylation
protein- cysteine S- palmitoyltransferase activity
ciliary basal body
cilium assembly
myosin complex
actin filament binding
proteasome complex
enrichment
- log10(pvalue)
count
b.
MeCP2 down
CRS up genes 187
palmitoyltransferase activity
protein palmitoylation
protein- cysteine S- palmitoyltransferase activity
non- motile cilium assembly
ciliary basal body
cilium assembly
myosin complex
actin filament binding
coreceptor activity
enrichment
c.
CRS down genes 63
MeCP2 up
glutamatergic synapse
synapse
postsynaptic density
neuron differentiation
cellular response to hypoxia
nucleus
cytoplasm
proteasome complex
protein kinase binding
dendritic spine
enrichment
d.
Clstn3
Rtn3
Axonal growth inhibition (RHOA activation)
Plxnd1
Rtn4
Ppp2r2a
Rhoa
Akt1
Nr3c1
Sorcs3
Vps26b
Dlg4
Rheb
Il1rap
Ptprd
Regulation of post neurotransmitter receptor activity
Dlgap3
Begain
Zdhhc17
Trans-synaptic signaling by trans-synaptic complex

Supplementary Fig. 16 Functional characterization of the transcripts whose CRS-associated expression differences were reduced following MeCP2 overexpression (250 genes). A total of 250 CRS-responsive transcripts exhibited reduced group-level differences under MeCP2 overexpression conditions (CC vs. NC: $|FC| > 1.3$, nominal $p < 0.05$; CM vs. NC: $p > 0.10$). This criterion was used to operationally define reduced group-level differences and does not imply statistical equivalence. **a–c** DAVID functional annotation clustering of CRS-responsive transcripts whose expression changes were attenuated by MeCP2 overexpression. The x-axis represents fold enrichment. Dot size indicates the number of genes per term, and dot color represents statistical significance ($-\log_{10}$(p-value)). **a** DAVID functional annotation clustering of the 250 transcripts using GO and UniProt frameworks. Shown are annotation groups with enrichment scores > 1 ($p < 0.05$), indicating meaningful functional convergence. **b, c** Sub-grouped enrichment analysis of transcripts that increased (187 genes, panel b) or decreased (63 genes, panel c) following CRS. Functional enrichment was assessed using the same criteria (enrichment score > 1, $p < 0.05$). **d** STRING-based protein–protein interaction network analysis of 22 genes associated with glutamatergic synapse function, identified from the enriched annotation clusters. Nodes represent proteins, edges denote predicted interactions, and arrows indicate the direction of CRS-associated expression changes (increase: red; decrease: blue). Three major interaction modules were observed, including networks associated with synaptic structure, trans-synaptic signaling, and axonal growth–related pathways.

a.

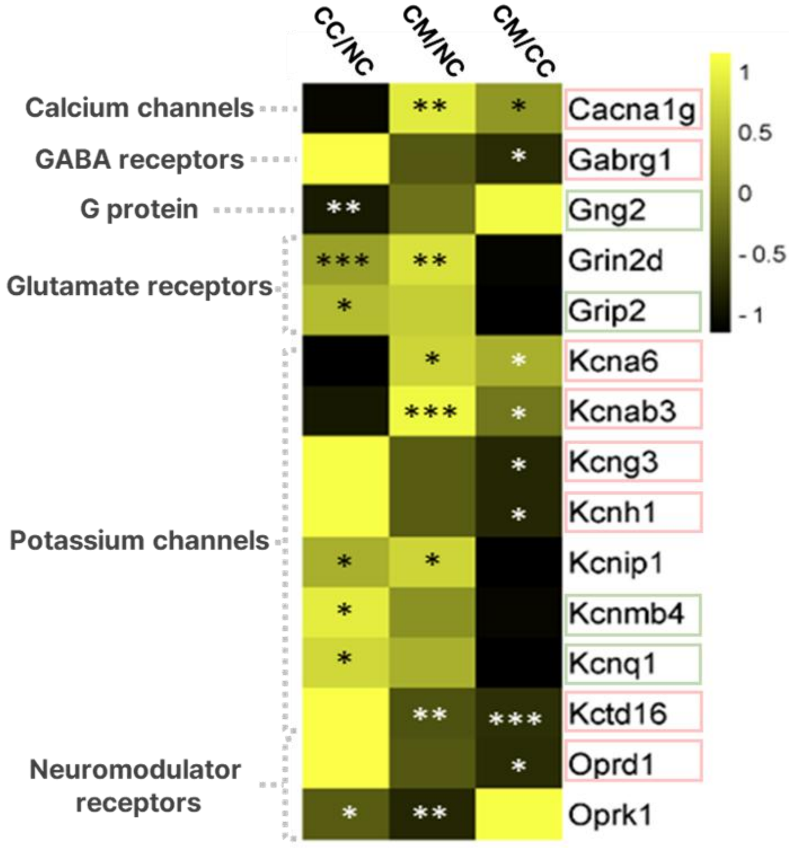

Supplementary Fig. 17 Functional category–based heatmap analysis of CRS-regulated genes in NAc D2R neurons. **a** Heatmaps show Z-score–normalized expression values for CRS-regulated transcripts grouped by predefined functional categories relevant to synaptic signaling, including ion channels, neurotransmitter receptors, GPCRs, and G-protein signaling components. CRS induced widespread dysregulation across these functional systems, whereas MeCP2 upregulation attenuated a subset of these transcriptional changes. Heatmap colors (black to yellow) indicate Z-scores (scale: −1 to 1). Pink boxes denote genes showing a significant MeCP2-associated difference (CM vs CC, nominal $p < 0.05$). Green boxes denote genes meeting the predefined attenuation pattern (CC vs NC, nominal $p < 0.05$; CM vs NC, nominal $p > 0.10$). Asterisks indicate nominal significance levels (*$p < 0.05$, **$p < 0.01$, ***$p < 0.001$). Sample sizes: NC = 8, CC = 11, CM = 8.

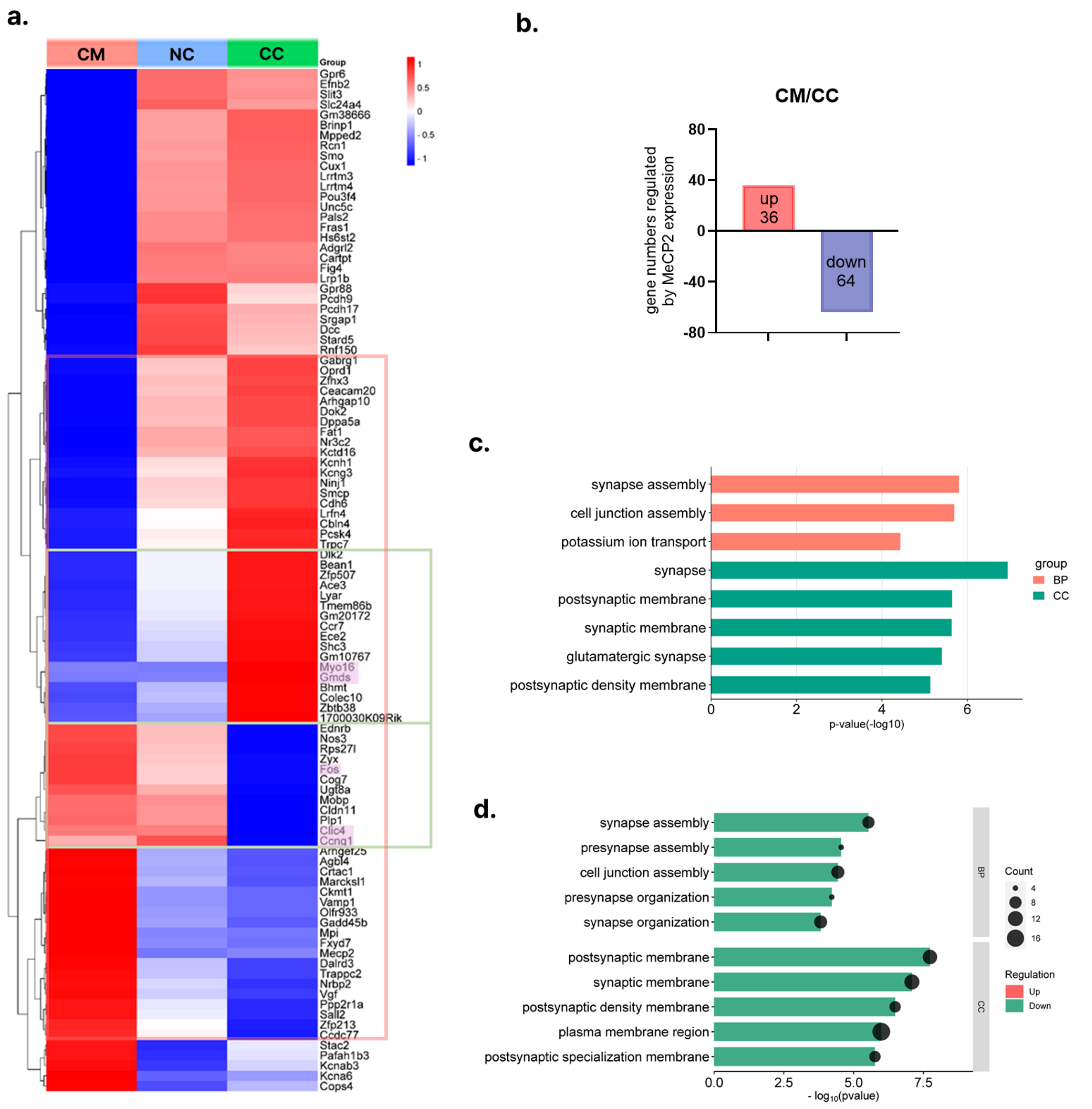

Supplementary Fig. 18 Genetic MeCP2 upregulation alters CRS-affected transcriptional programs in NAc D2R neurons. **a** Heatmap showing normalized expression values for 100 differentially expressed genes between CC and CM groups (36 upregulated and 64 downregulated; CM vs. CC, |FC| > 1.3, nominal p < 0.05). Genes highlighted in pink indicate transcripts showing the strongest reversal toward NC expression levels following MeCP2 upregulation, and green brackets denote clustered patterns of clear expression recovery. **b** Summary of genes upregulated (red) or downregulated (blue) by MeCP2 overexpression relative to the CRS condition. **c** Gene ontology (GO) enrichment analysis (biological process and cellular component categories) for the full set of regulated genes (FDR q < 0.05 for GO terms). **d** GO enrichment analysis performed separately on downregulated genes (green; FDR q < 0.05) and upregulated genes (red; no

significant enrichment detected). Circle size reflects gene count per GO term, and bar length represents significance ($-\log_{10}$(p-value)).

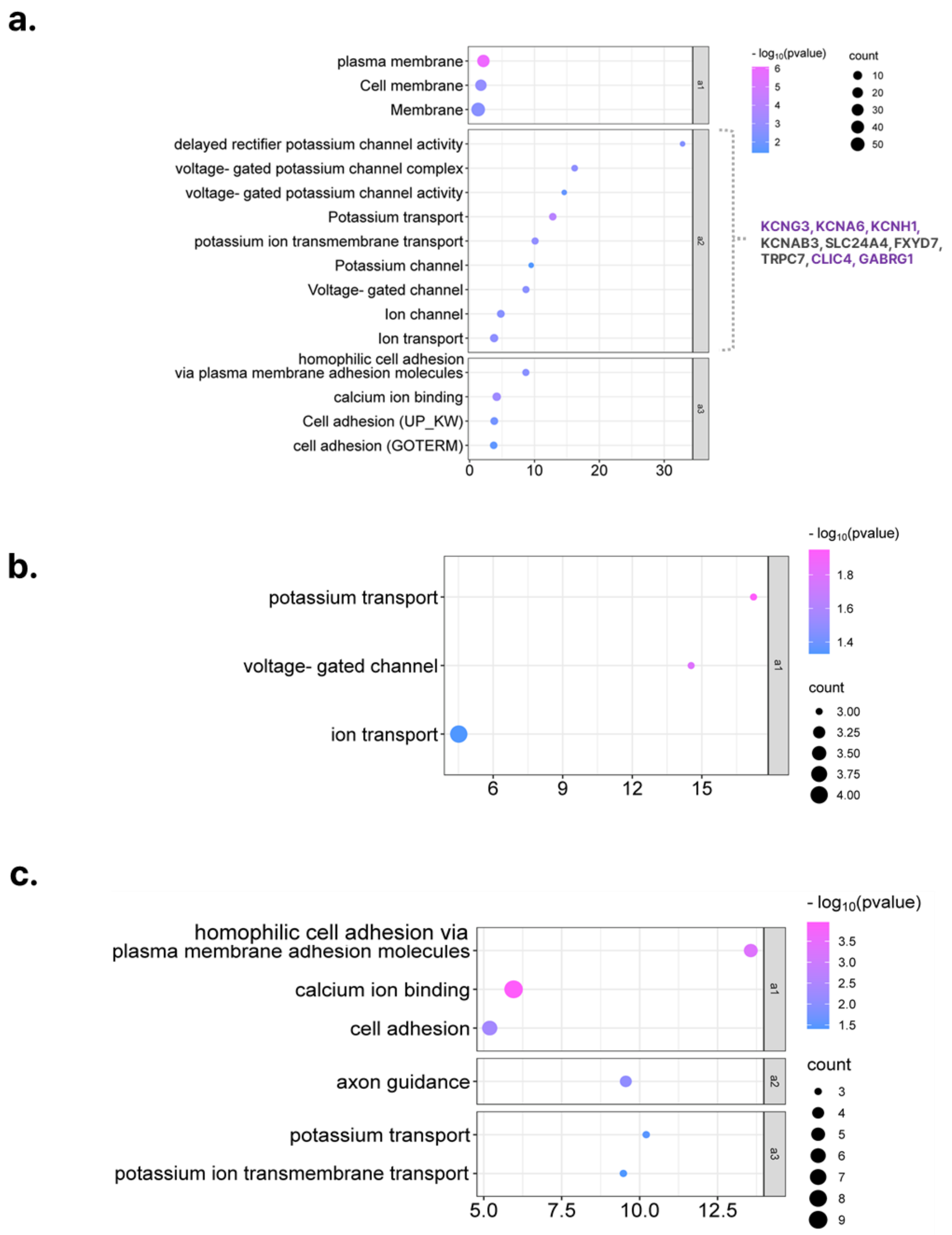

Supplementary Fig. 19 Functional enrichment analysis of MeCP2-responsive genes. A total of 100 genes showing altered expression in CM relative to CC (CM/CC, |FC| > 1.3, nominal $p < 0.05$) were examined using DAVID functional annotation clustering. **a** Summary of annotation clusters identified from the full gene set. **b,c** Separate enrichment analyses for upregulated (b) and downregulated (c) genes. For all analyses, only clusters with enrichment scores > 1 and nominal $p < 0.05$ (DAVID) are displayed. Functional terms include categories related to ion transport, membrane structure, synaptic organization, and adhesion pathways.

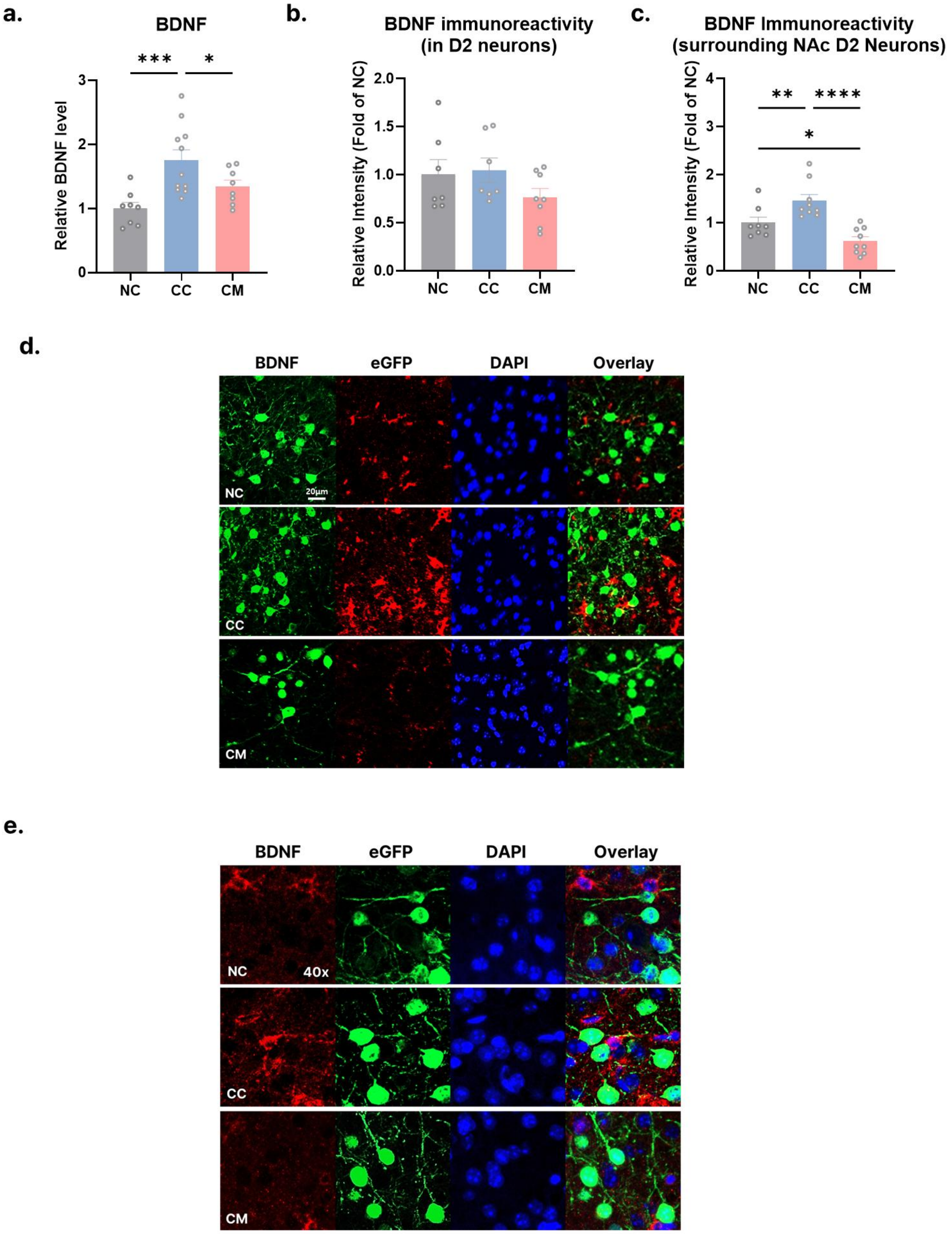

Supplementary Fig. 20 BDNF protein–level alterations associated with CRS and MeCP2 restoration **a** Bulk BDNF protein levels in the NAc measured by ELISA. One-way ANOVA revealed a significant group effect ($F = 8.066$, $p = 0.0021$). Post hoc Fisher's LSD tests showed a significant increase in the CRS group compared

with naïve controls (NC vs. CC, $p = 0.0006$) and a significant attenuation following MeCP2 overexpression (CC vs. CM, $p = 0.0401$), whereas BDNF levels in the CM group did not differ significantly from NC (NC vs. CM, $p = 0.1057$). **b** Quantification of BDNF immunoreactivity within eGFP-labeled NAc D2 neurons. No significant group differences were detected (one-way ANOVA, $F = 1.526$, $p = 0.2430$; Fisher's LSD: NC vs. CC, $p = 0.8064$; NC vs. CM, $p = 0.1922$; CC vs. CM, $p = 0.1241$). **c** Quantification of BDNF immunoreactivity in the neuropil surrounding NAc D2 neurons. One-way ANOVA revealed a robust group effect ($F = 14.61$, $p < 0.0001$ Post hoc Fisher's LSD test indicated increased BDNF immunoreactivity in the CRS group compared with NC (NC vs. CC, $p = 0.0186$), a reduction in the CM group relative to CC (CC vs. CM, $p < 0.0001$), and a modest difference between NC and CM (NC vs. CM, $p = 0.0247$). **d,e** Representative confocal images illustrating BDNF immunoreactivity (red), eGFP-labeled D2 neurons (green), and DAPI (blue) in the NAc across experimental groups. Images in **d** show low-magnification views, and **e** shows higher-magnification examples indicating sparse somatic BDNF signal within eGFP+ D2 neurons **and** prominent BDNF immunoreactivity in the surrounding perisomatic/neuropil region**,** without assigning a specific cellular source for the latter. Scale bars, 20 μm (d) and 40× objective (e). Data are presented as mean ± SEM.

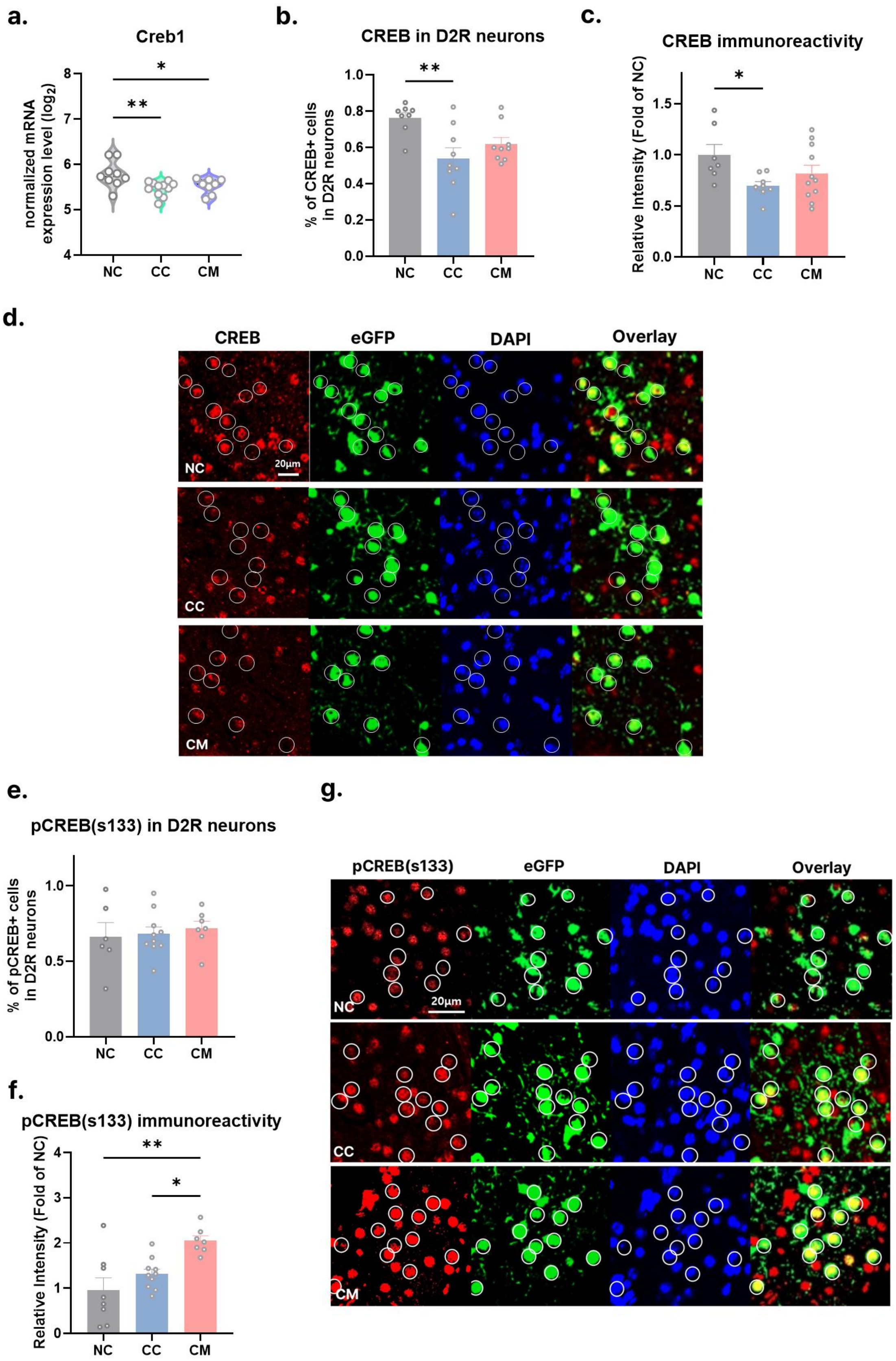
a.
Creb1
normalized mRNA expression level (log_2)
NC
CC
CM
b.
CREB in D2R neurons
% of CREB+ cells in D2R neurons
c.
CREB immunoreactivity
Relative Intensity (Fold of NC)
d.
CREB
eGFP
DAPI
Overlay
20μm
e.
pCREB(s133) in D2R neurons
% of pCREB+ cells in D2R neurons
f.
pCREB(s133) immunoreactivity
Relative Intensity (Fold of NC)
g.
pCREB(s133)
eGFP
DAPI
Overlay
20μm

Supplementary Fig. 21 Exploratory assessment of CREB and phospho-CREB signaling in virally labeled NAc D2R neurons under CRS and MeCP2 restoration. These measurements were collected as complementary signaling correlates accompanying behavioral/physiological attenuation and are not designed to establish mechanistic coupling between CREB and MeCP2, or a defined CREB→BDNF transcriptional pathway, nor do they provide projection-resolved insight. **a** *Creb1* mRNA expression levels in NAc D2R neurons. One-way ANOVA revealed a significant group effect ($F = 5.831$, $p = 0.0083$). Holm–Šídák's multiple comparisons test showed reduced *Creb1* expression in the CRS group compared with naïve controls (NC vs. CC, $p = 0.0093$), and a modest reduction in the CM group relative to NC (NC vs. CM, $p = 0.0374$), while CC and CM did not differ significantly (CC vs. CM, $p = 0.5950$). **b** Proportion of CREB-positive cells among eGFP-labeled NAc D2R neurons. One-way ANOVA indicated a significant group effect ($F = 6.322$, $p = 0.0065$). Post hoc Holm–Šídák tests revealed a significant reduction in the CC group relative to NC (NC vs. CC, $p = 0.0054$), whereas neither NC vs. CM ($p = 0.0658$) nor CC vs. CM ($p = 0.2057$) reached statistical significance. **c** Relative CREB immunoreactivity intensity in eGFP-labeled NAc D2R neurons (normalized to NC). Although the one-way ANOVA did not reach significance ($F = 3.222$, $p = 0.0584$), planned pairwise comparisons indicated reduced CREB immunoreactivity in the CC group relative to NC (two-tailed unpaired t-test, $p = 0.0124$), with no significant difference between NC and CM ($p = 0.1783$). Pairwise results are reported as planned comparisons. **d** Representative confocal images showing CREB immunoreactivity (red), eGFP-labeled D2R neurons (green), and DAPI (blue) across experimental groups. Scale bar, 20 μm. **e** Proportion of phospho-CREB (Ser133)–positive cells among eGFP-labeled NAc D2R neurons. One-way ANOVA revealed no significant group differences ($F = 0.1914$, $p = 0.8273$; Holm–Šídák tests, all $p = 0.9118$). **f** Relative pCREB (Ser133) immunoreactivity intensity in NAc D2R neurons (normalized to NC). One-way ANOVA showed a significant group effect ($F = 8.839$, $p = 0.0015$). Holm–Šídák's multiple comparisons test revealed increased pCREB intensity in the CM group compared with NC (NC vs. CM, $p = 0.0013$) and CC (CC vs. CM, $p = 0.0155$), whereas NC and CC did not differ significantly ($p = 0.1541$). **g** Representative confocal images illustrating pCREB (Ser133) immunoreactivity (red), eGFP-labeled D2R neurons (green), and DAPI (blue) across groups. Scale bar, 20 μm. Data are presented as mean ± SEM. *$p < 0.05$, **$p < 0.01$.

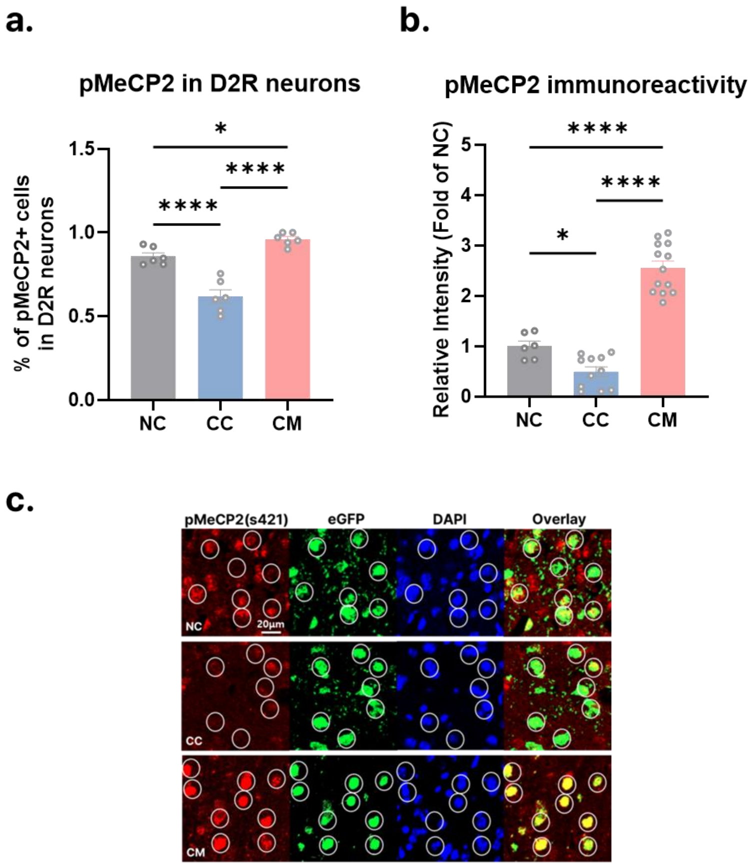

Supplementary Fig. 22 Phosphorylation-dependent regulation of MeCP2 in NAc D2R neurons under CRS and MeCP2 restoration. As an exploratory phospho-state readout, we quantified phospho-MeCP2 (Ser421) immunoreactivity in eGFP-labeled NAc D2R neurons across NC, CC, and CM groups. Because MeCP2 overexpression elevates total MeCP2 protein abundance in the targeted D2R neuronal population, pMeCP2(S421) changes should be interpreted in the context of MeCP2 dosage and are presented as supplementary correlates rather than mechanistic evidence. These measurements are not designed to establish causal signaling mechanisms or projection-resolved effects. **a** Proportion of phospho-MeCP2 (Ser421)–positive cells among eGFP-labeled NAc D2R neurons. One-way ANOVA revealed a significant group effect ($F = 39.77$, $p < 0.0001$). Holm–Šídák's multiple comparisons test indicated a significant reduction in the CRS group relative to naïve controls (NC vs. CC, $p < 0.0001$), a modest but significant difference between NC and CM ($p = 0.0200$), and a strong increase in the CM group compared with CC (CC vs. CM, $p < 0.0001$). **b** Relative phospho-MeCP2 (Ser421) immunoreactivity intensity in NAc D2R neurons (normalized to NC). One-way ANOVA demonstrated a highly significant group effect ($F = 86.55$, $p < 0.0001$). Post hoc Holm–Šídák analysis showed reduced pMeCP2 intensity in the CC group compared with NC ($p = 0.0179$), whereas MeCP2 overexpression markedly increased pMeCP2 intensity relative to both NC (NC vs. CM, $p < 0.0001$) and CC (CC vs. CM, $p < 0.0001$). **c** Representative confocal images showing phospho-MeCP2 (Ser421) immunoreactivity (red), eGFP-labeled D2R neurons (green), and DAPI (blue) across experimental groups. Scale bar, 20 μm. Data are presented as mean ± SEM. *$p < 0.05$, ****$p < 0.0001$.

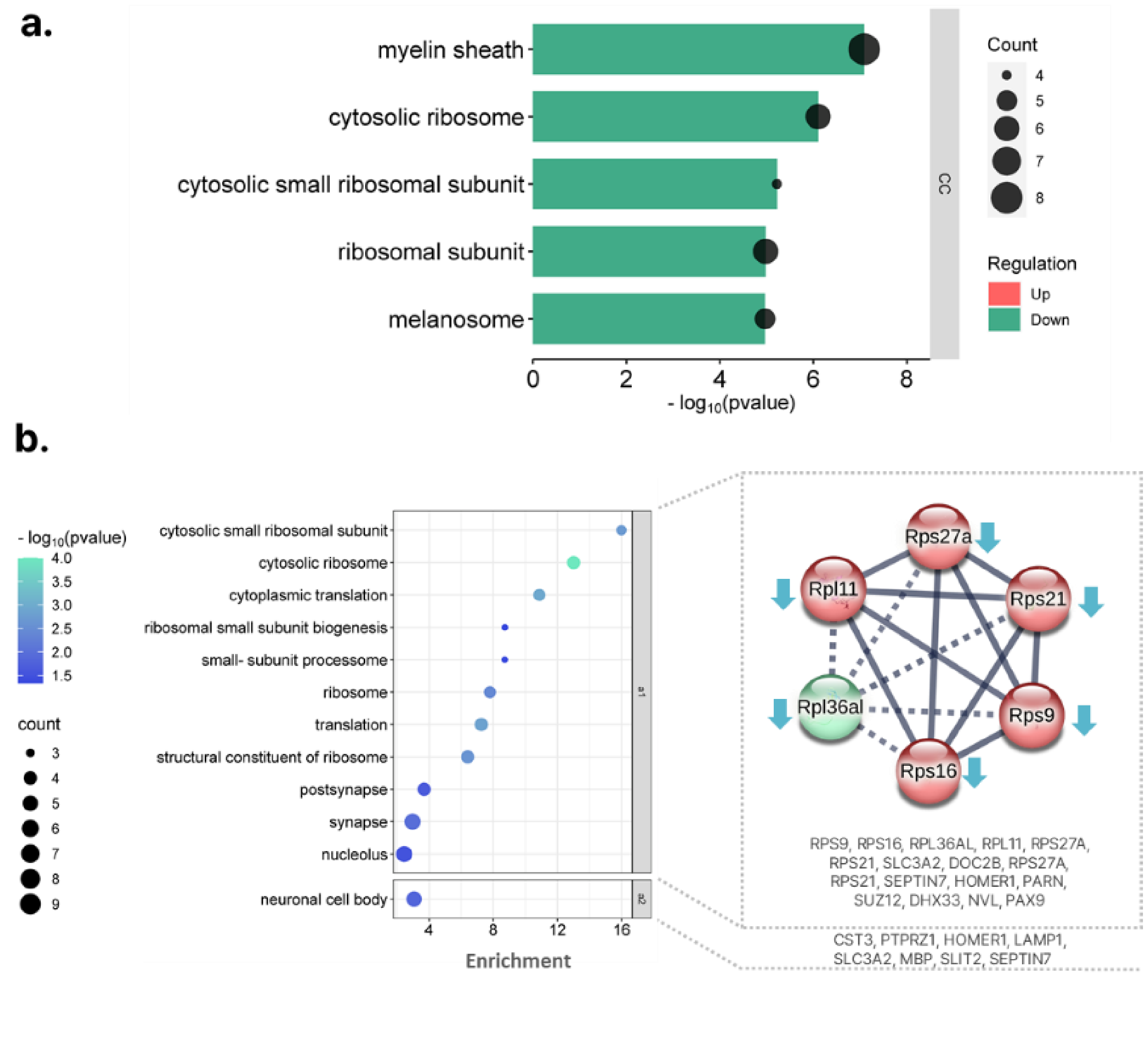

Supplementary Fig. 23 CRS-associated transcriptional changes in the ventral pallidum (VP). A total of 122 transcripts were altered by CRS in the VP (CC vs NC, |FC| > 1.3, nominal $p < 0.05$). **a** GO Cellular Component enrichment analysis of CRS-altered transcripts, performed separately by direction of CRS effect (CC vs NC): Up/Down**.** Significant enrichment was observed only for the CRS-downregulated subset under the stated criteria (FDR $q < 0.05$), whereas no significant terms were detected for the CRS-upregulated subset under the same criteria. **b** DAVID functional annotation clustering results for the CRS-altered transcripts. The x-axis represents fold enrichment. Dot size indicates gene count per term, and dot color represents statistical significance ($-\log_{10}$(p-value)). Right, STRING-based PPI network visualization for genes within the top annotation cluster(s).

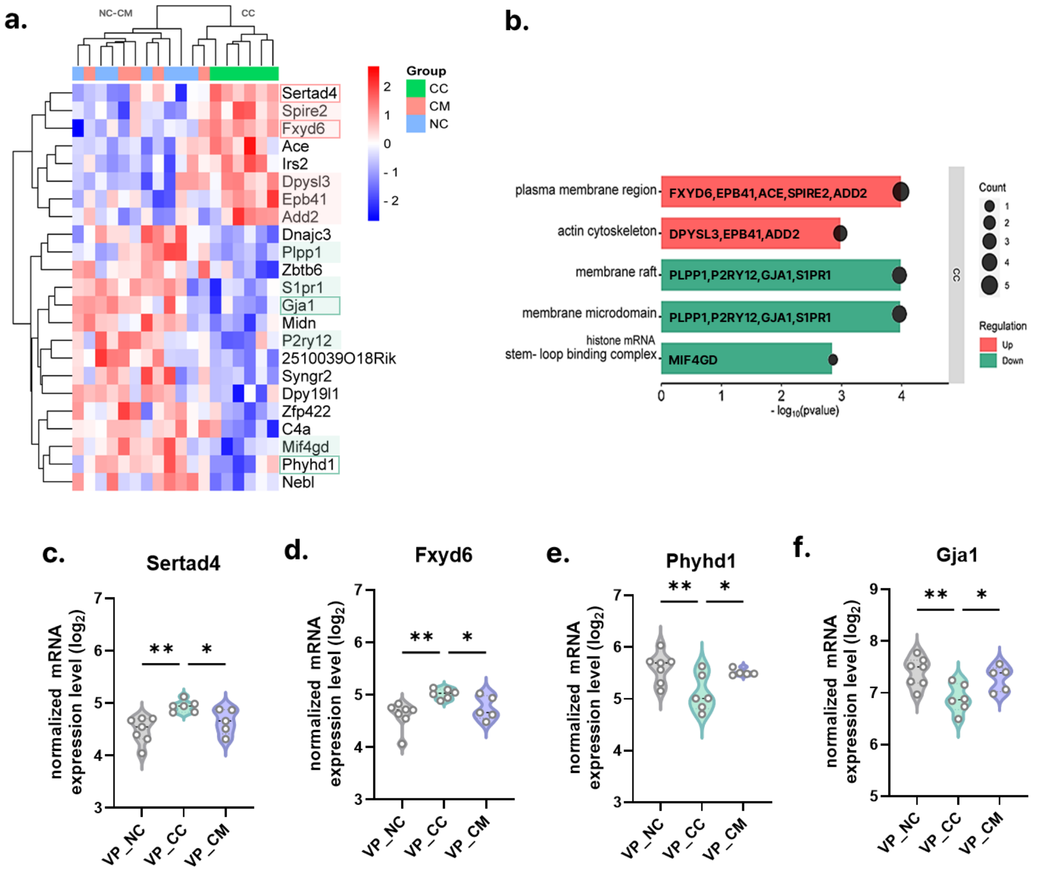

Supplementary Fig. 24 VP transcripts meeting predefined attenuation criteria following MeCP2 upregulation in NAc D2R neurons. Genes shown in this figure were selected based on predefined attenuation criteria **(**CC vs. NC: |FC| > 1.2 and nominal p < 0.05; CM vs. NC: nominal p > 0.10; CM vs. CC: nominal p < 0.05**),** consistent with partial normalization of CRS-associated molecular alterations. **a** Heatmap showing Z-score–normalized expression values for VP transcripts meeting the attenuation criteria, illustrating relative expression patterns across NC, CC, and CM groups **(**Z-score scale: −2 to +2**)**. **b** Gene ontology (GO) enrichment analysis for the Cellular Component category in VP, performed separately for CRS-upregulated (red bars) and CRS-downregulated (green bars) attenuated gene subsets (defined by the direction of the CRS effect in CC vs. NC). Enriched terms are shown with $-\log_{10}$ (p value), and dot size represents gene counts within each category. This analysis highlights membrane- and cytoskeleton-associated components among attenuated VP transcripts, without implying directional or causal regulation. **c**–**f** Violin plots illustrating the four VP transcripts classified as “expression difference minimized” in Fig. 7c (*Sertad4* (c), *Fxyd6* (d), *Phyhd1* (e), and *Gja1* (f)), validated at the expression level. Statistical analysis was performed using one-way ANOVA followed by Fisher’s LSD test. *Sertad4*: NC vs. CC, p = 0.0014; NC vs. CM, p = 0.2048; CC vs. CM, p = 0.0357. *Fxyd6*: NC vs. CC, p = 0.0028; NC vs. CM, p = 0.2728; CC vs. CM, p = 0.0462. *Phyhd1*: NC vs. CC, p = 0.0066; NC vs. CM, p = 0.5937; CC vs. CM, p = 0.0320. *Gja1*: NC vs. CC, p = 0.0041; NC vs. CM, p = 0.4481; CC vs. CM, p = 0.0328. Data are presented as mean ± SEM.

a.

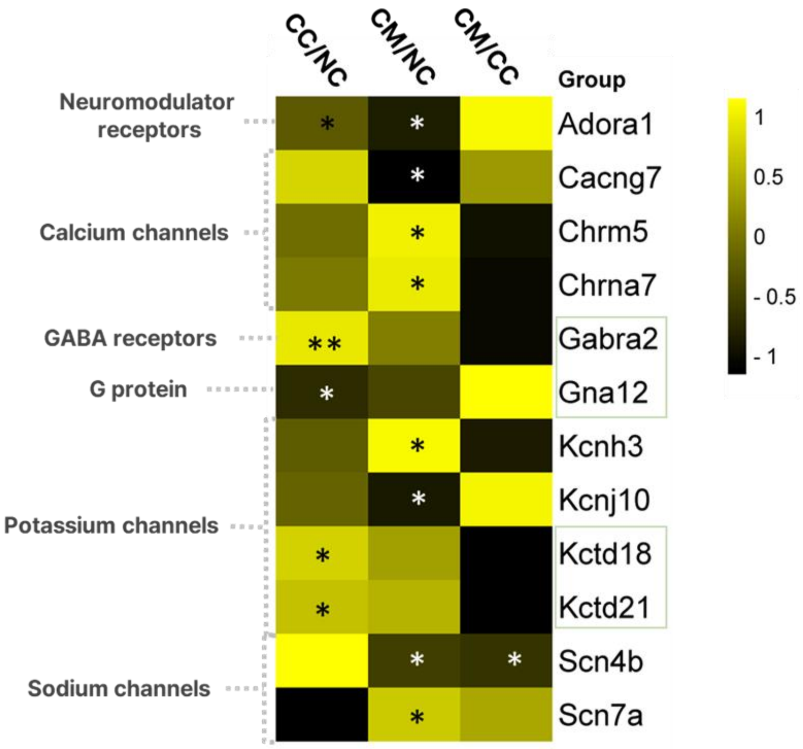

Supplementary Fig. 25 Expression patterns of synaptic- and activity-related transcripts in the ventral pallidum following CRS and MeCP2 upregulation. **a** Heatmap showing expression profiles of VP synaptic- and activity-related genes across experimental groups (NC, CC, CM). Green outlines indicate transcripts that met the predefined attenuation criteria following MeCP2 overexpression (CC vs. NC: nominal $p < 0.05$; CM vs. NC: nominal $p > 0.10$). Data are presented as Z-scored expression values. Asterisks denote statistical significance relative to NC (*$p < 0.05$, **$p < 0.01$).

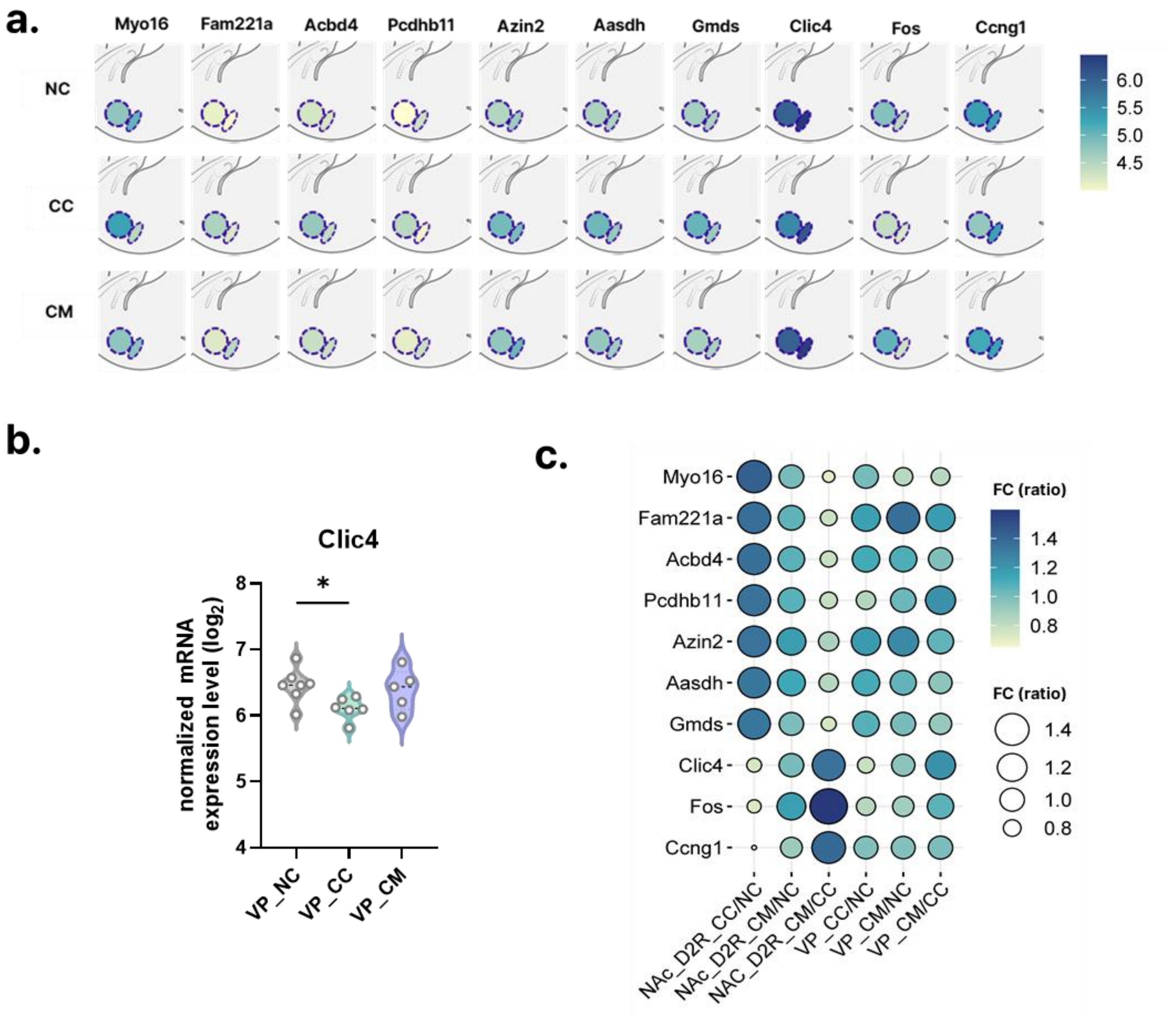

Supplementary Fig. 26 Supplementary Fig. 26 Cross-regional comparison of NAc-selected transcripts and their expression patterns in VP. **a** Spatial expression visualization of the top 10 transcripts selected in NAc D2R ROIs using the predefined attenuation criteria (CC vs NC: |FC| > 1.3 and nominal $p < 0.05$; CM vs NC: nominal $p > 0.30$; CM vs CC: nominal $p < 0.05$), shown across NAc D2R ROIs and VP ROIs (NC, CC, CM groups). Values represent normalized log_2 expression intensities. The colored label indicates the only transcript (Clic4) showing a parallel direction of change between NAc and VP. **b** Group-wise comparison of *Clic4* expression levels, illustrating a CRS-associated change and attenuation following MeCP2 overexpression (one-way ANOVA with post-hoc test; *$p < 0.05$). **c** Bubble plot summarizing cross-region changes for the same NAc-selected top 10 transcripts. The transcripts are shown in VP ROIs to test whether analogous expression changes are observed; however, VP did not exhibit a consistent corresponding pattern across these NAc-selected transcripts. For visualization, FC values are displayed as ratios (FC < 1 indicates decreased expression and FC > 1 indicates increased expression), and both bubble color and bubble size encode FC (ratio).
